# Immune-related adverse events associated with programmed cell death protein-1 and programmed cell death ligand 1 inhibitors for non-small cell lung cancer: a PRISMA systematic review and meta-analysis

**DOI:** 10.1186/s12885-019-5701-6

**Published:** 2019-06-10

**Authors:** Xiaoying Sun, Raheleh Roudi, Ting Dai, Shangya Chen, Bin Fan, Hongjin Li, Yaqiong Zhou, Min Zhou, Bo Zhu, Chengqian Yin, Bin Li, Xin Li

**Affiliations:** 1 Department of Dermatology, Yueyang Hospital of Integrated Traditional Chinese and Western Medicine, Shanghai University of Traditional Chinese Medicine, Shanghai, 200437 China; 2 Institute of Dermatology, Shanghai Academy of Traditional Chinese Medicine, Shanghai, 201203 China; 3 Oncopathology Research Center, Iran University of Medical Sciences, Tehran, 14496-14530 Iran; 4 Department of Toxicology, Shandong Academy of Occupational Health and Occupational Medicine, Shandong Academy of Medical Science, Jinan, 250062 Shandong China; 5 Department of Pharmacology & Experimental Therapeutics, Boston University School of Medicine, Boston, MA 02118 USA; 6 Department of Dermatology, Shaanxi Traditional Chinese Medicine Hospital, Xi’an, 710003 China

**Keywords:** Non-small cell lung cancer, Oncology, Programmed cell death protein-1, Programmed cell death ligand 1, Inhibitor, Immune-related adverse event, Meta-analysis, Systematic review

## Abstract

**Background:**

Programmed cell death protein-1 (PD-1) and programmed cell death ligand 1 (PD-L1) inhibitors have remarkable clinical efficacy in the treatment of non-small cell lung cancer (NSCLC); however, the breakdown of immune escape causes a variety of immune-related adverse events (irAEs). With the increasing use of PD-1/PD-L1 inhibitors alone or in combination with other therapies, awareness and management of irAEs have become more important. We aimed to assess the incidence and nature of irAEs associated with PD-1 and PD-L1 inhibitors for NSCLC.

**Methods:**

Articles from the MEDLINE, EMBASE, and Cochrane databases were searched through December 2017. The incidence of overall and organ-specific irAEs was investigated in all clinical trials with nivolumab, pembrolizumab, atezolimumab, durvalumab, and avelumab as single agents for treatment of NSCLC. We calculated the pooled incidence using R software with package Meta.

**Results:**

Sixteen trials were included in the meta-analysis: 10 trials with PD-1 inhibitors (3734 patients) and 6 trials with PD-L1 inhibitors (2474 patients). The overall incidence of irAEs was 22% (95% confidence interval [CI], 17–28) for all grades and 4% (95% CI, 2–6) for high-grade irAEs. The frequency of irAEs varied based on drug type and organ, and patients treated with PD-1 inhibitors had an increased rate of any grade and high-grade irAEs compared with patients who received PD-L1 inhibitors. Organ-specific irAEs were most frequently observed in, in decreasing order, the endocrine system, skin, pulmonary tract, and gastrointestinal tract. The total number of patients whose death was attributed to irAEs was 14 (0.34%), and most (79%) of these patients died because of pneumonitis. The median time to the onset of irAEs after the initiation of treatment was 10 weeks (interquartile range, 6–19.5 weeks) and varied depending on the organ system involved.

**Conclusions:**

The specificity of irAEs was closely associated with the mechanism of PD-1/PD-L1 antibodies involved in restarting anticancer immune attacks. Comprehensive understanding, timely detection, and effective management could improve the compliance of patients and guide the interruption of treatment.

**Electronic supplementary material:**

The online version of this article (10.1186/s12885-019-5701-6) contains supplementary material, which is available to authorized users.

## Background

Programmed cell death protein-1 (PD-1) is an important immunologic checkpoint inhibitor (ICI) that was discovered after cytotoxic T-lymphocyte-associated antigen-4 (CTLA-4). In 2002, a study using cloned antibodies in a mouse model showed that local immunosuppression can be abolished by blocking the binding of PD-1 and programmed cell death ligand 1 (PD-L1) [1]. This strategy established the basis for using PD-1/PD-L1 monoclonal antibodies to treat tumors. Since that time, the full leverage of the immune system’s potential has opened a new era of cancer treatment.

**Fig. 1:**
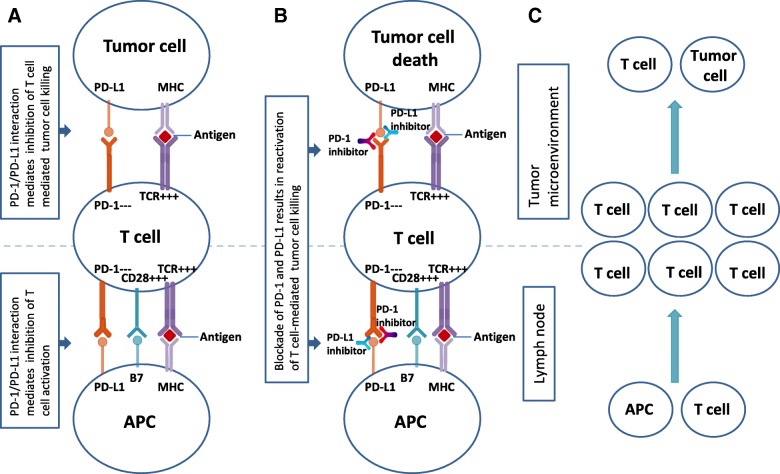
Mechanism of action of PD-1 and PD-L1 inhibitors (a) PD-1/PD-L1 binding inhibits T cell killing of tumor cells. b Blocking PD-L1 or PD-1 allows T cell killing. c Overview of the mechanism: APC T cell interaction T cell activation (i.e., cytokine secretion, lysis, proliferation, migration to tumor) Tumor microenvironment.

Inhibitors of PD-1 and PD-L1 act as ICIs by relaunching T cell-mediated tumor cell death programs (Fig. 1). These inhibitors have shown promising clinical efficacy in the treatment of non-small cell lung cancer (NSCLC), which remains a leading cause of cancer-related mortality [2]. PD-1 inhibitors pembrolizumab and nivolumab as well as the PD-L1 inhibitors atezolizumab, avelumab, and durvalumab have all been approved in succession by the Food and Drug Administration (FDA) for treating patients with metastatic NSCLC. Moreover, pembrolizumab was recently approved for first-line treatment of metastatic NSCLC (i.e., high PD-L1 expression, ≥50%; no epidermal growth factor receptor; or anaplastic lymphoma kinase mutation). In addition, pembrolizumab has been approved for adult and childhood cancer patients for the treatment of unresectable or metastatic solid tumors with the molecular features of high microsatellite instability or mismatch repair deficiency. The use of the same treatment for different diseases signifies a deeper and more comprehensive understanding of cancer and represents an important milestone in precision medicine.

Many clinical trials have been conducted to assess the efficacy and safety of PD-1/PD-L1 inhibitors used in combination with oncolytic virotherapy [3], anti-CTLA-4 antibodies [4, 5], targeted therapy [6], chemotherapy [7], or other means [8]. The increase in combined applications has made it difficult to evaluate safety. For example, grade 3–4 treatment-related adverse events (trAEs) were reported in 37, 33, and 48% of patients in two cohorts of one study [4] and in another study [5], respectively, involving treatment with nivolumab plus ipilimumab. It should be noted that these studies did not focus on irAEs, and it is not possible to distinguish which drug of a combination causes trAEs. Such safety assessment does not provide a unified reference for clinicians. Therefore, it is essential to conduct a meta-analysis and systematic review to evaluate the irAEs of PD-1 and PD-L1 inhibitors alone in the treatment of NSCLC.

Inhibitors of PD-1 and PD-L1 interfere with normal mechanisms of immune tolerance while inhibiting tumor immune escape. The increase in immune activation caused by these inhibitors in normal tissues may be responsible for various types of significant irAEs, which include endocrine, skin, pulmonary, gastrointestinal, hepatic, renal, neurologic, cardiac, and hematologic autoimmune diseases. IrAEs can negatively influence a patient’s quality of life and interrupt oncology treatment; therefore, sufficient knowledge, on-time monitoring, and appropriate management of these events are important. Although two reports have reviewed the safety of PD-1 and PD-L1 inhibitors in the treatment of malignancies, the results were not entirely consistent [9, 10], and to our knowledge, no systematic reviews or meta-analyses of irAEs associated with PD-1/PD-L1 inhibitors for NSCLC using the Common Terminology Criteria as the outcome metric have been published in the literature. Thus, we conducted a systematic review and meta-analysis of the qualifying literature aiming to assess the incidence and nature of irAEs resulting from the use of anti-PD-1 or anti-PD-L1 antibodies to treat NSCLC.

## Methods

Developed using the stepwise approach to systematic reviews described by Kelley and Kelley [11], the protocol of our systematic review and meta-analysis has been registered in the International Prospective Register of Systematic Reviews (PROSPERO) (No. CRD42016045886) and has been previously published [12].

### Data sources and searches

A systematic literature search for relevant articles published in any language through December 2017 was conducted using EMBASE, MEDLINE via PubMed, and the Cochrane Central Register of Controlled Trials. Two investigators (XYS and SYC) together determined the final search strategy. The detailed search strategy for PubMed was provided in Additional file 1: Table S1. Searches were repeated immediately before the final analysis to identify additional studies for inclusion, and manual searches were also conducted from references of related literature, both of which were done by XYS. Articles published as full texts were optimal to extract data related to irAEs in detail and to allow a quality assessment of the trials included in the meta-analysis. Therefore, unpublished studies were not searched or included. EndNote X7 software was used to store references.

### Eligibility criteria and study selection

We included randomized controlled trials (RCTs), single-arm trials, and case reports that reported irAEs in patients with a diagnosis of NSCLC who were receiving anti-PD-1 antibodies (e.g., nivolumab or pembrolizumab) or anti-PD-L1 antibodies (e.g., atezolizumab or durvalumab). The included patients could have received previous oncologic therapy, but patients were excluded if they had received anti-PD-1 antibodies or anti-PD-L1 antibodies in combination with other treatments such as chemotherapy or other immunotherapies. XYS and SYC screened the titles and abstracts of the search output to assess whether the studies met the inclusion criteria, as defined by the protocol. Then, they independently read the full text of all potentially eligible studies for further discrimination. Discrepancies between the two authors regarding study inclusion were resolved via consensus with the assistance of the senior authors (HJL and XL).

### Outcomes

Incidence assessment was based on the number of global and organ-specific irAEs (i.e., endocrine, gastrointestinal, hepatic, pulmonary, renal, and skin diseases). IrAE severity was recorded as grade 1–5 based on version 3 or 4 of the Common Terminology Criteria for Adverse Events of the National Cancer Institute (Bethesda, MD, USA). Grades 3 through 5 were considered high-grade irAEs.

### Data extraction

Two authors (XYS and SYC) independently extracted and recorded the data using Excel 2007. The data were recorded on a predesigned extraction list. Full texts were obtained through databases or by contacting the corresponding authors. Discrepancies regarding data records between the two authors were resolved via consensus with the assistance of a senior author (XL).

Clinical trials were used to assess the incidence of irAEs. Author(s), clinical trial information, study design, enrollment size, type and dose of monoclonal antibodies, version of the Common Terminology Criteria for Adverse Events, frequency of irAEs and organ-specific irAEs, and the median time to onset were captured for the systematic review and meta-analysis.

Case reports were used to describe the diversity of irAEs qualitatively. Patient characteristics, previous oncologic treatment, cancer outcome (i.e., oncologic response or progressive disease), the nature of each irAE, as well as irAE onset, treatment, and outcome were all recorded.

### Quality assessment

The Cochrane Collaboration “risk of bias” tool was used to assess the risk of bias and to evaluate the quality of RCTs that were included in the systematic review and meta-analysis. Additionally, this tool was also used to determine selection bias, performance bias, detection bias, attrition bias, reporting bias, and other biases [13]. The quality of non-RCTs was assessed by the Newcastle–Ottawa Scale (NOS) [14]. Two authors (XYS and TD) independently conducted this quality assessment, and agreement was reached via consensus with the assistance of a senior author (XL).

### Data synthesis and analysis

The primary objective of this study was to determine the incidence of irAEs for each treatment group (i.e., anti-PD-1 group and anti-PD-L1 group). The effect size was a single proportion of incidence from each study because, some of the included studies were single-armed. Statistical heterogeneity and inconsistency between the selected studies were assessed using the Q statistic and *I*^2^ statistic, respectively. The *I*^2^ cutoffs used to determine inconsistency were very low (< 25%), low (25 to < 50%), moderate (50 to < 75%), and large (> 75%). For each analysis, the 95% confidence interval (CI) of the weighted average was calculated. If heterogeneity was not rejected by the Q-test, the original random-effects model was used because our analysis involved different populations and the random-effects model incorporates interstudy heterogeneity into the calculation [15, 16]. Before the analyses, rate consolidation was conducted using five methods (untransformed, log transformation, logit transformation, arcsine transformation, and Freeman–Tukey double arcsine transformation), and the method that yielded results closest to a normal distribution was selected. All analyses were conducted using the R package Meta and function Metaprop (R version 3.4.4 [2018-3-15]; R Foundation, Vienna, Austria), which is better able to achieve single-rate meta-analysis than the method described in the published protocol for the R package Meta. When the number of event counts was 0, the classic value of 0.5 was used instead. The incidences and their 95% CIs are presented as forest plots. Subgroup analyses according to the type (anti-PD-1 or anti-PD-L1) and brand of antibody drugs were performed to avoid heterogeneity. Small-study effects were assessed through a potentially more robust qualitative (Doi plot) and quantitative (LFK index) approach with MetaXL (MetaXL version 5.3; EpiGear International, Noosa, Queensland, Australia) [17]. Influence analysis was conducted by deleting each study from the model once to observe how the omission influenced our overall findings.

## Results

### Literature search

**Fig. 2:**
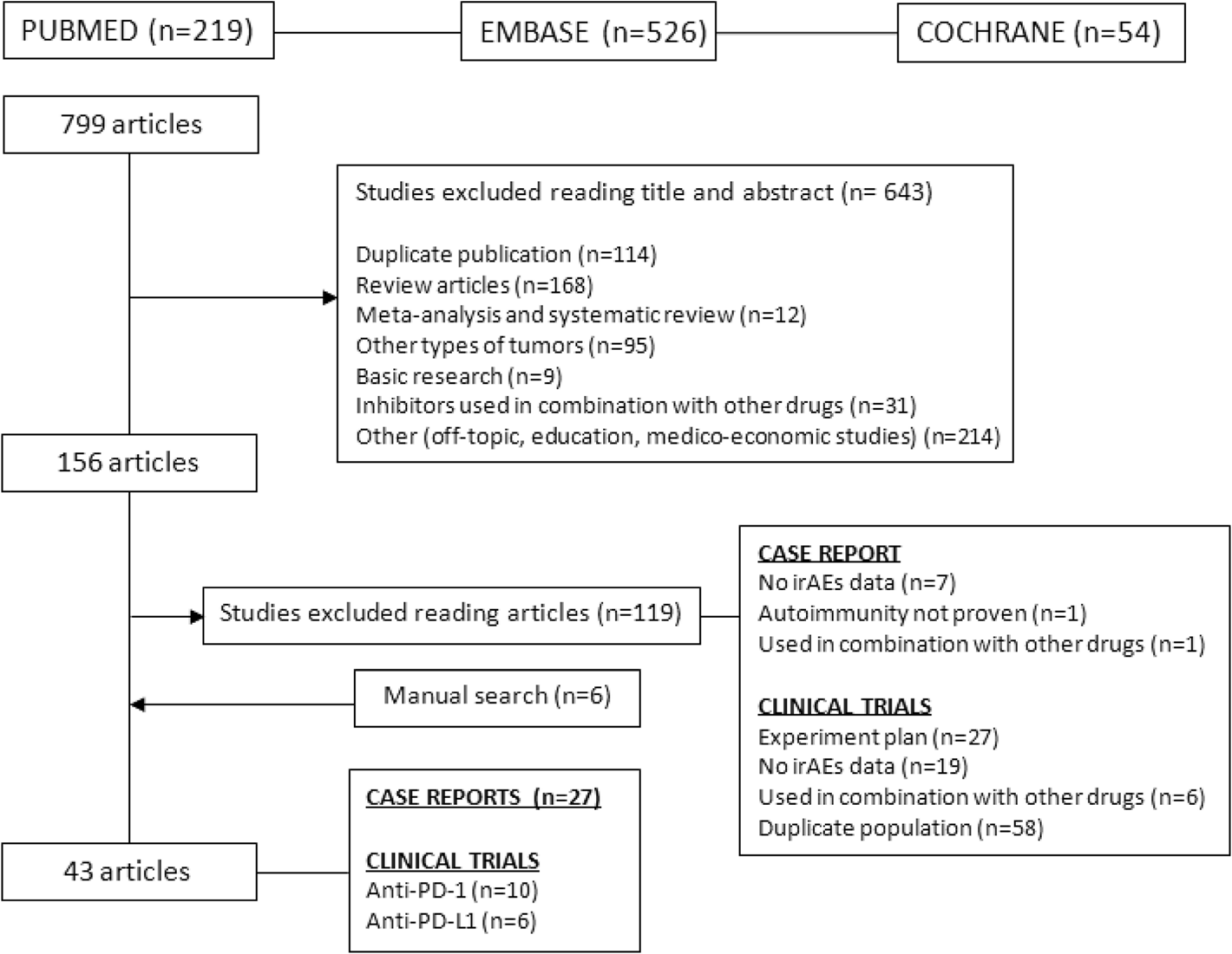
Study flow chart for identification and selection of included studies

Seven hundred ninety-nine articles were identified by searching the literature databases, and six additional articles were retrieved by manual searches. The titles and abstracts were then read for these 805 articles, and 643 articles were excluded because they were duplicate articles, included non-NSCLC tumors, or reported the use of the inhibitors in combination with other drugs or were review articles, basic research articles, or off topic. One hundred sixty-two articles were ultimately fully reviewed; of these, 43 studies were considered relevant for the present study (16 clinical trials and 27 case reports, Fig. 2). Per PRISMA guidelines, a reference list of all excluded studies, except for those that were duplicates, is provided with the reason(s) for exclusion before each reference according to Fig. 2 (Additional file 2).

The meta-analysis thus included 16 clinical trials in which patients were treated, based on the labeling of the products (e.g., nivolumab at 1 mg/kg, 3 mg/kg, or 10 mg/kg every 2 weeks; pembrolizumab at 2 mg/kg or 10 mg/kg every 3 weeks; durvalumab at 10 mg/kg every 2 weeks; atezolizumab at 1200 mg every 3 weeks; avelumab at 10 mg/kg every 3 weeks).

### Incidence of irAEs: data from clinical trials

#### General characteristics

Sixteen clinical trials were included in the meta-analysis for the current study. Only six articles recorded the total and organ-specific irAEs. The remaining 10 articles described only organ-specific irAEs. Because several irAEs can occur in the same patient, the sum of the incidence of organ-specific irAEs does not represent the overall irAE incidence. Therefore, 2029 patients from six clinical trials (three trials concentrated on anti-PD-1 treatment, and three trials focused on anti-PD-L1 treatment) were included in the meta-analysis to assess the global incidence of irAEs [18–23]. In addition, 6208 patients from 16 clinical trials were included to assess the incidence of organ-specific irAEs (Table 1) [18–33].

**Table 1:** Characteristics of studies included for the meta-analysis

Trial	Design	Design details	Enrollment size (no.)	Drug	Dose (mg/kg)	CTC for AE version
Antonia (2017) [4]	RCT	double-blind, multicenter, phase III	475	Durvalumab	10, q2w	4
Borghaei (2015) [5]	RCT	open-label, multicenter, phase III	287	Nivolumab	3, q2w	4
Brahmer (2015) [6]	RCT	open-label, multicenter, phase III	131	Nivolumab	3, q2w	4
Carbone (2017) [7]	RCT	open-label, multicenter, phase III	267	Nivolumab	3, q2w	4
Fehrenbacher (2016) [8]	RCT	open-label, multicenter, phase II	142	Atezolizumab	1200 mg, q3w	4
Garassino (2018) [9]	Single-arm	open-label, multicenter, phase II	444	Durvalumab	10, q2w	4
Garon (2015) [10]	Single-arm	open-label, multicenter, phase Ib	495	Pembrolizumab	2, q3w; 10, q3w; 10, q2w	4
Gettinger (2016) [11]	Single-arm	open-label, multicohort, phase I	52	Nivolumab	3, q2w	4
Gettinger (2015) [12]	RT	dose-escalation cohort expansion, phase I	129	Nivolumab	1,3,10,q2w	3
Herbst (2016) [13]	RCT	open-label, multicenter, phase II/III	682	Pembrolizumab	2, 10, q3w	4
Peters (2017) [14]	Single-arm	open-label, multicenter, phase II	659	Atezolizumab	1200 mg, q3w	4
Reck (2016) [15]	RCT	open-label, multicenter, phase III	154	Pembrolizumab	200 mg q3w	4
Rittmeyer (2017) [16]	RCT	open-label, multicenter, phase III	609	Atezolizumab	1200 mg q3w	4
Rizvi (2015) [17]	Single-arm	open-label, multicenter, phase II	117	Nivolumab	3, q2w	4
Gulley (2017) [18]	Single-arm	open-label, multicenter, phase II	184	Avelumab	10, q2w	4
Waterhouse (2018) [24]	Single-arm	open-label, community-based, phase IIIb/IV	1420	Nivolumab	3, q2w	4

CTC for version, Common Terminology Criteria for Adverse Events version; RCT, Research clinical trial; n/a, not available

Most (15/16) studies were not blind. Nine of the studies were randomized, of which eight were controlled, and seven of the studies were single-armed. Fourteen studies were multicentered. The median follow-up duration for all clinical trials was 13 months (interquartile range [IQR], 10–15 months). It is noteworthy that patients with a history of autoimmune disease met the exclusion criteria in all studies.

#### Global incidence of irAEs

**Fig. 3:**
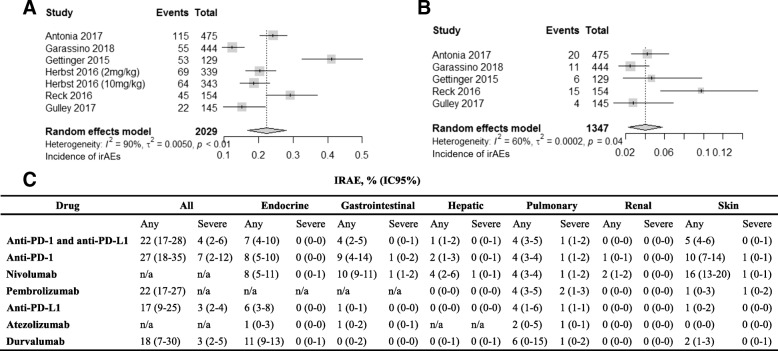
Incidence of global immune-related adverse events (irAEs) associated with anti-PD-1 and anti-PD-L1 antibodies: all grade (a) and severe grade (b). Organ-specific irAEs associated with anti-PD-1 and anti-PD-L1 antibodies (c)

The overall incidence of irAEs reported with anti-PD-1 and anti-PD-L1 treatment was 22% (95% CI, 17–28; *I*^2^, 90%) for all grades and 4% (95% CI, 2–6; *I*^2^, 60%) for high grades (Fig. 3a and b). The incidence of all-grade irAEs varied depending on drug type, from 27% (95% CI, 18–35; *I*^2^, 88%) for patients treated with PD-1 to 17% (95% CI, 9–25; *I*^2^, 91%) for patients receiving PD-L1. This drug effect was analogously confirmed in high-grade irAEs, which showed incidences ranging from 7% (95% CI, 2–12; *I*^2^, 65%) for PD-1 to 3% (95% CI, 2–4; *I*^2^, 10%) for PD-L1 (Additional file 3: Figures S1 and S2). No trials investigated the incidence of any grade irAEs in patients treated with atezolizumab. Further, only two clinical trials each assessed the incidence of irAEs in patients treated with pembrolizumab [21, 22] or durvalumab [18, 19]; one trial each assessed the incidence of any grade irAEs in patients treated with nivolumab [20] or avelumab [23], and one trial assessed the incidence of high-grade irAEs in patients treated with pembrolizumab [22]. Therefore, these studies were not assessed in this meta-analysis (Additional file 3: Figures S3 and S4).

#### Incidence of organ-specific irAEs

Organ-specific irAEs were observed with the highest incidence in the endocrine system, skin, pulmonary tract, and gastrointestinal tract, which were affected in 7% (95% CI, 4–10), 5% (95% CI, 4–6), 4% (95% CI, 3–5), and 4% (95% CI, 2–5) of cases, respectively. Hepatic organs were affected in only 1% (95% CI, 1–2) of cases, and other events, such as nephrologic, neurologic, cardiologic, and hematologic diseases, were rare (< 1%).

Nearly all skin, endocrine, gastrointestinal, hepatic, and renal irAEs were low-grade. High-grade irAEs represented 1% of pulmonary events (95% CI, 1–2; Fig. 3c and Additional file 3: Figures S5 to S43).

Patients treated with PD-1 inhibitors tended to show a higher incidence of organ-specific irAEs compared with those treated with PD-L1 inhibitors, especially in the gastrointestinal tract [9% (95% CI, 4–14%) vs. 1% (95% CI, 0–1%)] and skin [10% (95% CI, 7–14%) vs. 1% (95% CI, 0–2%)], although the rates of high-grade irAEs were equivalent in the two groups (Fig. 3c; Additional file 3: Figures S14 to S19 and Figures S40 to S46).

#### Incidence of death related to irAEs

Thirteen clinical trials reported the incidence of death attributed to irAEs. In these studies, death occurred in 14 (0.34%) patients. Most deaths (79%) were related to pneumonitis.

### Nature of irAEs: data from case reports and retrospective studies

#### General characteristics

Our research identified 35 patients from 27 case reports with at least one irAE [34–60]. Three of these patients presented with several irAEs. The general characteristics of the patients are shown in Additional file 4: Table S2. Thirty-two patients received anti-PD-1 treatment. Of these, 74% received nivolumab treatment. Just one patient received anti-PD-L1 treatment. Seventeen (54%) patients failed at least one course of chemotherapy before receiving anti-PD-1/anti-PD-L1 antibodies. The irAEs manifested within a median of 10 (IQR, 6–19.5) weeks. Ten patients continued to receive immunotherapy after irAE diagnosis. The patients received a median treatment of 6 (IQR, 2.25–11.25) cycles.

#### Nature of irAEs

The most common organ-specific irAEs described in the 27 case reports of 35 patients occurred in the endocrine system. Eleven (31%) cases were recorded and occurred, on average, within 8.5 weeks of anti-PD-1/anti-PD-L1 treatment (Additional file 4: Table S2 and Table S3).

Adrenal crisis and diabetes were rare, and one case study of diabetes reported that blood glucose levels were elevated when the blood samples were tested before treatment [36]. The irAEs reported in 6 of 11 clinical trials resolved when treated by steroids and other symptomatic drugs. Two cases of diabetes were also controlled with insulin therapy. One report described life-threatening adrenal crisis following nivolumab treatment [45].

Pneumonitis was the most frequent pulmonary adverse effect and is of particular relevance to NSCLC. It was reported in up to 4% of all-grade irAEs and 1.5% of high-grade irAEs in clinical trials [18–33]. Pneumonitis tended to occur early, at a median time of 4.5 weeks (IQR, 2.75–6.25) in four patients described in three case reports, all of whom were treated with nivolumab (Additional file 4: Table S3) [37, 40, 42]. The median duration of immune treatment was 4.5 (IQR, 2.75–6.25) cycles, implying that immunotherapy did not stop immediately when pneumonitis occurred. Most of the initial symptoms were mild and presented as progressive dyspnea, dry cough, and fever. These symptoms were readily confused with lung cancer, which delayed the diagnosis. In addition to clinical symptoms, the noted characteristics included typical changes in ground glass opacity, reticular opacity, and consolidation distributed in the periphery on CT.

Cutaneous irAEs are mostly described as rash and pruritus. These are commonly reported in clinical trials, second only to endocrine irAEs, and tend to be mild. Of the three case reports of skin irAEs, two patients developed grade 3 skin adverse reactions: psoriasis occurred at week 6 in one patient, while bullous pemphigoid occurred at week 8 in the other patient (Additional file 4: Table S3). Suspension of immunotherapy was the principal treatment. The combination of topical or intravenous steroids with other therapies such as phototherapy also promoted disease relief.

Nephrologic irAEs were rare. Most (80.0%; 4/5) cases were diagnosed as acute interstitial nephritis by evidence of kidney biopsy. Immune therapy was withdrawn, and prednisone therapy was initiated from 60 mg/day and tapered over 1 month. The patients showed a prompt return to baseline kidney function [44].

Colitis was the most frequent gastrointestinal irAE in clinical trials. However, possibly because of mostly mild symptoms, we only retrieved one case report describing pancreatitis [53], which presented after two cycles of nivolumab with typical anorexia, vomiting, and back pain. There were no abnormalities on CT or magnetic resonance cholangiopancreatography. Prednisone was administered up to 4 mg/kg/day, and amylase and lipase levels eventually returned to the normal range.

#### Other irAEs

Neurologic irAEs reported in the literature included one case of cerebral vasculitis/encephalitis [39] and two cases of myasthenia gravis [46, 51] (Additional file 4: Table S3). The case of cerebral vasculitis/encephalitis resolved through treatment with surgery and steroids. Although immune therapy was discontinued, and steroids, plasmapheresis, and other adapted treatments were combined, one patient with myasthenia gravis died, and one had persistent myasthenia gravis symptoms.

Moreover, we retrieved two case reports describing pericardial effusion [55, 60] and two case reports describing myocarditis [50, 52], one of which was fatal. The patient with myocarditis presented with acute right heart failure and may have died of lethal cardiac arrhythmia 1 day after hospitalization. At autopsy, a T cell-rich myocardial infiltrate was identified, but an infectious cause was not identified [50]. In addition, authors in various studies reported one case each of agranulocytosis [56], warm-autoimmune hemolytic anemia [57], and immune thrombocytopenia [58]. After administering methylprednisolone or dexamethasone and other symptomatic treatments, three cases of hematologic irAEs were resolved.

#### Quality assessment

Most studies were open label, single-arm trials and therefore had a high risk of selection bias, performance bias, and detection bias. This problem is mostly unavoidable in clinical studies of oncology owing to ethical considerations. Several researchers used a blinded, independent, central review to assess tumor primary endpoints but not adverse events [18, 22, 26, 30]. All included RCTs had low risks of reporting bias, attrition bias, and other biases, and all non-RCTs had a high or medium quality according to NOS for quality assessment. Further details about the quality assessment are available in Additional file 5: Table S4 and Table S5. The LFK index values for quantitative assessment of the small-study effects were more than 1 in the analysis of incidence of both all-grade (LFK: 1.19) and severe-grade (LFK: 1.17) irAEs with anti-PD-1 and anti-PD-L1, which indicated that positive publication bias existed (Additional file 6: Figure S45).

## Discussion

The application of immunotherapy, especially PD-1/PD-L1 inhibitors, has provided unprecedented efficacy gains in NSCLC treatment. With the promotion of single and joint application, two existing problems are becoming increasingly evident: unpredictable efficacy and inevitable irAEs. To the best of our knowledge, our study is the first meta-analysis and systematic review of irAEs following treatment of NSCLC with anti-PD-1 or anti-PD-L1. The most significant finding of the current study was that the incidence of any grade irAEs with anti-PD-1 treatment was higher than that with anti-PD-L1 treatment, and the trend was consistent with the incidence of high-grade irAEs. This finding was different from or even the opposite of the results of a previous systematic review that compared PD-1 inhibitors with PD-L1 inhibitors (any grade, 16% vs. 11%; high grade, 3% vs. 5%) [61]. This may be explained by the increased number of included articles and the more explicit definition of irAEs.

Organ-specific irAEs occurred most frequently in the endocrine organs and skin, followed by the gastrointestinal tract and the pulmonary tract. Liver-related and kidney-related adverse reactions were rare. Adverse events affecting the heart, blood, and nerves have also been reported. The most frequent endocrine adverse effect was thyroid dysfunction, which included six cases of hypothyroidism, one case of hyperthyroidism, and one case of thyroiditis. This irAE is usually detected 3–4 weeks after drug intervention, with a trend of quick onset of hyperthyroidism and short lag time to the development of hypothyroidism [49]. The exact pathophysiology of PD-1-associated thyroid dysfunction remains unclear. However, unlike the slow progression of typical Hashimoto’s thyroiditis, the rapid course of PD-1-associated thyroid dysfunction suggests inflammatory destruction and mechanisms similar to painless thyroiditis.

Symptoms such as fatigue, weight loss, cold intolerance, and palpitations are common in oncology patients and have a high possibility of being missed or misdiagnosed. Delays in diagnosis and management may lead to worsening symptoms and further complications. Therefore, specialists should be vigilant and rigorously monitor confusing clinical symptoms and changes in laboratory parameters to promptly identify and manage irAEs. For example, thyroid changes are common after treatment with PD-1 inhibitors and require active laboratory monitoring. TSH levels should thus be measured before initiating anti-PD-1/PD-L1 treatment and every 4–6 weeks during treatment because nivolumab is administered intravenously every 2 weeks and pembrolizumab is administered intravenously every 3 weeks [24]. In addition, we recommend that Common Terminology Criteria self-reported by oncologists be used to supplement physician evaluation [62].

Tissue pneumonia should be scrutinized to distinguish between immune-related and non-immune-related disease. Asepticity of the bronchial and alveolar samples, CD8+ lymphocyte-based alveolitis, and the presence of lung immune-reactive processes are important references for the diagnosis of immune-mediated organizing pneumonitis [63]. Fortunately, most irAEs are highly sensitive to corticosteroids, but recurrence due to premature tapering of steroids and possibly the long tissue half-life of nivolumab is common [37]. One study on colon irAEs reported that steroid sensitivity can be predicted by the colonic mucosal concentration of tumor necrosis factor alpha [64]. Additionally, Stroud et al. [65] used the interleukin 6 receptor antagonist tocilizumab to effectively treat steroid-refractory irAEs. However, randomized trials are required to more rigorously clarify the relative efficacy and safety of these drugs.

It should be noted that case reports were included in this review to qualitatively complement the quantitative conclusions of the companion meta-analysis, and the ability to draw statistical conclusions from case studies is limited because only novel or rare irAEs tend to be reported in the literature. Despite this limitation, the current body of case studies does show that several serious irAEs have occurred in patients treated with anti-PD-1/PD-L1 agents. However, immune-related deaths are rare (0.34%), which is consistent with a review of ICIs that reported that less than 1% of all patients had fatal events associated with ICI [66]. Nonetheless, the incidence of death due to irAEs in patients with NSCLC was twice the incidence reported in a previous study of malignancy (0.17%) [9].

The leading cause of death in the studies we reviewed was pneumonitis, presumably because organ-specific irAEs are related to tumor type. However, systematic reviews of other tumor types will be required to verify this finding.

An increasing number of lung cancer patients receive first- or second-line immunotherapy; therefore, early identification and management of irAEs is critical. Several guidelines and reports on patient care have thus been released. The European Society for Medical Oncology Guidelines Committee has developed clinical practice guidelines for the use of five PD-1/PD-L1 inhibitors alone, the use of ipilimumab alone, and the use of combined ipilimumab and nivolumab [67]. Further, a consensus statement from the Melanoma Nursing Initiative on managing adverse events offers a positive, comprehensive nursing approach that gives clinicians resources to guide clinical care for patients who develop irAEs while receiving anti-PD-1/PD-L1 therapy [68]. Additionally, the Clinical Committee of the Endocrine Society has approved the acute treatment guideline developed by Higham et al. [69] for treating endocrine irAEs with checkpoint inhibitors. Finally, one report each has shared experiences in managing cutaneous irAEs [70] and neurologic irAEs [71].

We did not examine differences in the incidence of irAEs at different treatment doses because most reported data only focus on trAEs, which do not exactly match the definition of irAEs. However, the only RCT that has evaluated the efficacy of pembrolizumab in patients with previously treated PD-L1-positive NSCLC and advanced NSCLC reported that adverse events of special interest based on immune etiology occurred in 69 of 339 patients (20%) in the 2 mg/kg group and 64 of 343 patients (19%) in the 10 mg/kg group [21]. A meta-analysis of anti-PD-1/PD-L1 treatment for malignancy also reported that the development of irAEs was unrelated to the dose of PD-1/PD-L1 inhibitors [9]. Another clinical trial compared the effect of different infusion times on the incidence of irAEs in patients with previously-treated advanced NSCLC and found that the overall frequency of organ-specific irAEs was higher in the 30-min infusion group than in the 60-min infusion group; however, most events were mild, and the frequency of high-grade irAEs was similarly low in both groups [33].

Our analysis showed that the incidence of irAEs is higher with PD-1 inhibitors than with PD-L1 inhibitors. PD-L1 inhibitors prevent PD-L1 from binding to PD-1, but they do not prevent programmed cell death ligand 2 (PD-L2) from binding to PD-1, which may allow potential immune-related toxicity attributed to PD-L2 blockade to be avoided [72].

Another meta-analysis compared the efficacy of PD-1 and PD-L1 inhibitors in treating NSCLC and concluded that the tumor response rate was higher in the anti-PD-1 group than in the anti-PD-L1 group [10]. The mechanism of tumor response is that PD-1 inhibitors prevent the binding of protein PD-1 on the surface of activated T cells to PD-L1 and PD-L2 on the surface of tumor cells, thus restoring the function of T cells in the immune system. PD-1 and PD-L1 are distributed not only on T cells and tumor cells but also on various immune cells such as B cells and macrophages. Thus, toxicity to normal organs is difficult to avoid. In addition, recent research has shown that PD-L1-positive extracellular vesicles participate in systemic resistance to anti-tumor immunity when they are secreted by melanoma cells into the tumor microenvironment and circulation [73]. This finding expanded a previously unrecognized PD-1/PD-L1 interaction mechanism and, to some extent, explained the correlation between high risk rate and high response rate. This association is a reminder that specialists should seek a balance between gains in efficacy and frequency/severity of adverse reactions.

Indeed, it should be noted that the role of PD-1/PD-L1 inhibitors and agonists in predicting response rates and improving efficacy also affects the incidence of irAEs. For example, the main factors affecting the pembrolizumab response rate fall into two categories: those associated with tumor neoepitope burden, such as high tumor mutational burden, and those related to T cell-inflamed tumor microenvironment, such as PD-L1 [74]. A combination of these predictive factors thus helps to guide drug selection and to predict irAEs. It has been shown that the number of genetic mutations in patients [75], the presence of CMTM6 molecules [76], CD28/B7 status [77], and intestinal microbes [78] may be associated with new antigens on the surface of cancer cells, PD-L1 half-life, T cell activity, and T cell recruitment, respectively. However, considering the high cost of tumor mutational burden genome analysis and the widespread application limits, novel markers that can be directly detected by blood tests are needed, and the threshold for these markers needs to be determined by standardized experiments. Furthermore, it may be necessary to discover new organ-specific indicators in order to predict individual differences in organ-specific irAEs. In summary, dynamic monitoring of the whole process of drug penetration based on clear indicators and a more thorough understanding of molecular mechanisms are needed to establish an irAE-prediction system.

CTLA-4 inhibitors are a class of ICIs that were discovered before PD-1/PD-L1 inhibitors. The overall incidence of irAEs with these ICIs has been reported to be 72% for all-grade malignant tumors and 24% for high-grade malignant tumors [79]. This incidence was significantly higher than that from treatment with PD-1/PD-L1 inhibitors, reflecting the fact that these drugs have different mechanisms of initiating anticancer immune attacks. Anti-PD-1 and anti-CTLA-4 treatments both induce the expansion of specific subsets of tumor-infiltrating exhausted-like T cells, and anti-CTLA-4 additionally engages ICOS+ Th1-like CD4 T cells [80]. The fact that these treatments affect different T cell subsets explains why these therapies are more effective when combined than when used alone. Indeed, the histopathology of irAEs reflects these immunologic characteristics. For example, in PD-1 inhibitor-induced colitis, CD8+ T cells exist in the lamina propria and epithelium, whereas the same T cells exist in the lamina propria in CTLA-4 inhibitor-induced colitis [64]. The FDA has approved the use of PD-1/PD-L1 inhibitors in combination with CTLA-4 antibodies for the treatment of NSCLC, which represents an opportunity and a challenge for specialists to more thoroughly understand the irAEs induced by the two ICIs in combination.

The irAEs reported after the deadline of the literature search tended to be high-grade and fatal and include hypophysitis [81], type I diabetes [82], and renal tubular acidosis [83]. Renal tubular acidosis was reported for the first time as an irAE associated with nivolumab. There is a notable case report of a patient who experienced successive secondary adrenal insufficiency, thrombocytopenia, and colitis, which were attributed to durvalumab [81]. In addition, it was recently shown that influenza vaccination in lung cancer patients can reduce the risk of complications but might increase the frequency of irAEs [84].

Our research had the following strengths. Firstly, our study is the first meta-analysis and systematic evaluation of irAEs after treatment of NSCLC with anti-PD-1 or anti-PD-L1 and reconciled previous contradictory results. Secondly, the use of the Common Terminology Criteria as an outcome metric for irAEs is clinically relevant and readily interpretable by practicing oncologists. Furthermore, the utilization of case reports in studies of this nature is relatively novel, and a comprehensive review of the treatment and prognosis status of irAEs compensated for the weakness related to publication bias to some extent. In addition, the integration of the Cochrane risk of bias tool allowed us to highlight that clinical studies of immunotherapy are often not randomized studies, but single-arm trials. Finally, the imputation of event counts of zero with 0.5 allowed trials that did not observe any adverse events to be included in the meta-analysis and contribute valuable data. These advantages increased the relevance and improved the quality of our results, and strengthened the validity of the conclusions.

On the other hand, several limitations should be noted. First of all, only 16 studies that specifically reported irAEs were included in this report, and the remaining 10 studies only reported trAEs or did not further clarify the incidence of any grade and high-grade irAEs. This low proportion may be due to the need for a clear definition of irAE and the corresponding difficulty in diagnosing irAEs. Indeed, certain adverse events, such as colitis, can occur because of a non-immune drug response. Therefore, there is an urgent need to publish a standardized method that specifies quantifiable criteria for irAEs and non-irAEs. The irAEs described in this study include those that were directly described in the included clinical trials as well as select adverse events and adverse events of special interest based on a prespecified list of terms from the Medical Dictionary for Regulatory Activities [85]. Secondly, this analysis was based on articles that were published and indexed in one of the databases or registries that were searched. It is certainly plausible that publication of trials with unfavorable adverse event profiles, which may be more common and more likely to have adverse consequences for patients, was never pursued by the industry sponsor. Thirdly, the included clinical studies described single-arm trials, which are justified by the humanistic approach to care required in oncology research. This limitation does not allow the use of odds ratios to assess the risk of adverse reactions. Moreover, three PD-L1 inhibitors were each approved for listing in the last 2 years; therefore, there are fewer published clinical trials that focus on these drugs. The incidence of irAEs for PD-L1 inhibitors thus needs to be further evaluated by future updated studies. Finally, given that this study involved an aggregate data meta-analysis, the potential for ecological fallacy existed.

## Conclusion

Wider applications of an increasing number of new irAEs are being recognized. A thorough understanding of the pathogenesis and pathologic features of these adverse reactions can help to clarify the definition of irAEs and to establish a predictive system to reduce morbidity. At present, management guidelines for irAEs are gradually being established. Timely and effective treatment of irAEs is necessary to improve patient compliance and guide decision-making for interruption of immunotherapy. More in-depth clinical studies are required to identify biomarkers that are useful predictors of both treatment efficacy and adverse effects and thereby allow specialists to determine the optimal balance for effective oncology treatment.

## Additional files

Additional file 1: **Table S1.** Search strategy for PubMed. (DOCX 18 kb)

Additional file 2: Reference list of all excluded studies. (DOCX 81 kb)

Additional file 3: **Figure S1.** to **Figure S2.** Global irAEs (all grade and severe grade) associated with anti-PD-1 and anti-PD-L1 drugs. **Figure S3.** to **Figure S4.** Global irAEs (all grade and severe grade) associated with pembrolizumab and durvalumab. **Figure S5 to Figure S43.** Organ-specific (i.e., skin, endocrine, gastrointestinal, hepatic, and renal diseases) irAEs (all grade and high grade) associated with anti-PD-1 and anti-PD-L1, anti-PD-1, nivolumab, pembrolizumab, anti-PD-L1, atezolizumab, and durvalumab at all dosages. **Figure S44.** Death related to irAEs. (DOCX 9781 kb)

Additional file 4: **Table S2.** General characteristics of patients receiving anti-PD-1/anti-PD-L1 antibodies, as described in case reports (*n* = 35). **Table S3.** Organ-specific immune-related adverse events. (DOCX 32 kb)

Additional file 5: **Table S4.** The Cochrane Collaboration’s tool for assessing risk of bias of RCTs. **Table S5.** Newcastle–Ottawa Scale (NOS) for quality assessment of non-RCTs. (DOCX 43 kb)

Additional file 6: **Figure S45.** Qualitative and quantitative assessment of small-study effects on incidence of global irAEs with anti-PD-1 and anti-PD-L1. (DOCX 93 kb)

## Abbreviations

CI: confidence interval
CTLA-4: cytotoxic T-lymphocyte-associated antigen-4
FDA: Food and Drug Administration
ICI: immunologic checkpoint inhibitor
irAE: immune-related adverse event
NOS: Newcastle-Ottawa Scale
NSCLC: non-small cell lung cancer
PD-1: programmed cell death protein-1
PD-L1: programmed death ligand 1
RCT: randomized controlled trial
trAE: treatment-related adverse event
TSH: thyroid stimulating hormone
