# Supplementary material for: Immune-related adverse events associated with programmed cell death protein-1 and programmed cell death ligand 1 inhibitors for non-small cell lung cancer: a PRISMA systematic review and meta-analysis
Source: BMC Cancer. 2019 Jun 10;19:558. doi: 10.1186/s12885-019-5701-6 (PMC6558759; doi:10.1186/s12885-019-5701-6)
Supplement: Supplementary file 1 — Table S1. Search strategy for PubMed. (DOCX 18 kb) [file 12885_2019_5701_MOESM1_ESM.docx]

**Supplementary Table 1 Search strategy for PubMed**

| Query | Search term |
| --- | --- |
| #1 | Safety OR security OR side effects OR adverse events OR adverse effects |
| #2 | “Carcinoma, nonsmall-cell lung” (Medical Subject Heading) OR nonsmall cell lung cancer OR NSCLC |
| #3 | Anti-PD-1 OR anti-PD-L1 OR pembrolizumab OR nivolumab OR atezolizumab OR avelumab OR durvalumab OR lambrolizumab OR visilizumab OR BMS936558 OR BMS935559 OR AMP-224 OR AMP-514 OR Keytruda OR MK-3475 OR MDX-1106 OR ONO-4538 OR Opdivo OR MEDI-4736 OR MPDL3280A |
| #4 | #1 AND #2 AND #3 |
