# Supplementary material for: Immune-related adverse events associated with programmed cell death protein-1 and programmed cell death ligand 1 inhibitors for non-small cell lung cancer: a PRISMA systematic review and meta-analysis
Source: BMC Cancer. 2019 Jun 10;19:558. doi: 10.1186/s12885-019-5701-6 (PMC6558759; doi:10.1186/s12885-019-5701-6)
Supplement: Supplementary file 2 — Reference list of all excluded studies. (DOCX 81 kb) [file 12885_2019_5701_MOESM2_ESM.docx]

**Additional File 2 - Reference list of all excluded studies**

**Studies excluded reading title and abstract except for those that were duplicates (n= 529)**

**Review articles: 168**

1. Socinski MA: Advances in Immuno-Oncology: Immune Checkpoint Inhibitors in Non-Small Cell Lung Cancer - Introduction. Seminars in oncology 2015, 42:S1-S2.

2. Yao S, Zhu Y, Chen L: Advances in targeting cell surface signalling molecules for immune modulation. Nature Reviews Drug Discovery 2013, 12(2):130-146.

3. Sznol M, Chen L: Antagonist antibodies to PD-1 and B7-H1 (PD-L1) in the treatment of advanced human cancer. Clinical Cancer Research 2013, 19(5):1021-1034.

4. Lipson EJ, Forde PM, Hammers HJ, Emens LA, Taube JM, Topalian SL: Antagonists of PD-1 and PD-L1 in Cancer Treatment. Seminars in oncology 2015, 42(4):587-600.

5. Reichert JM: Antibodies to watch in 2014: Mid-year update. mAbs 2014, 6(4):799-802.

6. Reichert JM: Antibodies to watch in 2015. mAbs 2015, 7(1):1-8.

7. Homet Moreno B, Ribas A: Anti-programmed cell death protein-1/ligand-1 therapy in different cancers. British Journal of Cancer 2015, 112(9):1421-1427.

8. Pall G: ASCO update 2015: lung cancer. Memo - Magazine of European Medical Oncology 2015, 8(4):213-215.

9. Amos SM, Duong CPM, Westwood JA, Ritchie DS, Junghans RP, Darcy PK, Kershaw MH: Autoimmunity associated with immunotherapy of cancer. Blood 2011, 118(3):499-509.

10. Gettinger S, Herbst RS: B7-H1/PD-1 blockade therapy in non-small cell lung cancer: Current status and future direction. Cancer Journal (United States) 2014, 20(4):281-289.

11. Hall RD, Gray JE, Chiappori AA: Beyond the standard of care: A review of novel immunotherapy trials for the treatment of lung cancer. Cancer Control 2013, 20(1):22-31.

12. Manson G, Norwood J, Marabelle A, Kohrt H, Houot R: Biomarkers associated with checkpoint inhibitors. Annals of Oncology 2016, 27(7):1199-1206.

13. Flies DB, Sandler BJ, Sznol M, Chen L: Blockade of the B7-H1/PD-1 pathway for cancer immunotherapy. Yale Journal of Biology and Medicine 2011, 84(4):409-421.

14. Alatrash G, Jakher H, Stafford PD, Mittendorf EA: Cancer immunotherapies, their safety and toxicity. Expert Opinion on Drug Safety 2013, 12(5):631-645.

15. Jacob JA: Cancer immunotherapy researchers focus on refining checkpoint blockade therapies. JAMA - Journal of the American Medical Association 2015, 314(20):2117-2119.

16. Anagnostou VK, Brahmer JR: Cancer immunotherapy: A future paradigm shift in the treatment of non-small cell lung cancer. Clinical Cancer Research 2015, 21(5):976-984.

17. Cancer immunotherapy: enthusiasm and reality aligning at last. The Lancet Oncology 2015, 16(5):475.

18. Postel-Vinay S, Aspeslagh S, Lanoy E, Robert C, Soria JC, Marabelle A: Challenges of phase 1 clinical trials evaluating immune checkpoint-targeted antibodies. Annals of Oncology 2016, 27(2):214-224.

19. Wong KM, Capasso A, Eckhardt SG: The changing landscape of phase I trials in oncology. Nature Reviews Clinical Oncology 2016, 13(2):106-117.

20. Kyi C, Postow MA: Checkpoint blocking antibodies in cancer immunotherapy. FEBS Letters 2014, 588(2):368-376.

21. Gedye C, van der Westhuizen A, John T: Checkpoint immunotherapy for cancer: Superior survival, unaccustomed toxicities. Internal Medicine Journal 2015, 45(7):696-701.

22. Alexander W: The checkpoint immunotherapy revolution: What started as a trickle has become a flood, despite some daunting adverse effects, new drugs, indications, and combinations continue to emerge. P and T 2016, 41(3):185-191.

23. Melero I, Grimaldi AM, Perez-Gracia JL, Ascierto PA: Clinical development of immunostimulatory monoclonal antibodies and opportunities for combination. Clinical Cancer Research 2013, 19(5):997-1008.

24. Kline J, Gajewski TF: Clinical development of mAbs to block the PD1 pathway as an immunotherapy for cancer. Current Opinion in Investigational Drugs 2010, 11(12):1354-1359.

25. Garon EB: Current Perspectives in Immunotherapy for Non-Small Cell Lung Cancer. Seminars in oncology 2015, 42:S11-S18.

26. Tsao AS: Current Readings: Window-of-Opportunity Trials for Thoracic Malignancies. Seminars in Thoracic and Cardiovascular Surgery 2014, 26(4):323-330.

27. Goss G, Spaans J: Current state of the art. Journal of Thoracic Oncology 2015, 10(9):S79-S80.

28. Benhuri B, Belum VR, Lacouture M: Dermatologic adverse events during treatment with anti-PD1 inhibitors. Journal of the American Academy of Dermatology 2016, 74(5):AB116.

29. Roach C, Zhang N, Corigliano E, Jansson M, Toland G, Ponto G, Dolled-Filhart M, Emancipator K, Stanforth D, Kulangara K: Development of a Companion Diagnostic PD-L1 Immunohistochemistry Assay for Pembrolizumab Therapy in Non-Small-cell Lung Cancer. Applied Immunohistochemistry and Molecular Morphology 2016, 24(6):392-397.

30. Berman D, Korman A, Peck R, Feltquate D, Lonberg N, Canetta R: The development of immunomodulatory monoclonal antibodies as a new therapeutic modality for cancer: The Bristol-Myers Squibb experience. Pharmacology and Therapeutics 2015, 148:132-153.

31. Eigentler TK, Hassel JC, Berking C, Aberle J, Bachmann O, Grünwald V, Kähler KC, Loquai C, Reinmuth N, Steins M et al: Diagnosis, monitoring and management of immune-related adverse drug reactions of anti-PD-1 antibody therapy. Cancer Treatment Reviews 2016, 45:7-18.

32. Sabanathan D, Kong B, Byrne N, Wegener V, Eslick G, Nagrial A, Hui R, Kefford R, Gurney H, Carlino MS: Differences in the patterns of anti-PD1 (PD1) toxicity between tumor streams. Asia-Pacific Journal of Clinical Oncology 2016, 12:68.

33. Martinalbo J, Bowen D, Camarero J, Chapelin M, Démolis P, Foggi P, Jonsson B, Llinares J, Moreau A, O'Connor D et al: Early market access of cancer drugs in the EU. Annals of Oncology 2016, 27(1):96-105.

34. Tartari F, Santoni M, Burattini L, Mazzanti P, Onofri A, Berardi R: Economic sustainability of anti-PD-1 agents nivolumab and pembrolizumab in cancer patients: Recent insights and future challenges. Cancer Treatment Reviews 2016, 48:20-24.

35. Menis J, Litière S, Tryfonidis K, Golfinopoulos V: The European Organization for Research and Treatment of Cancer perspective on designing clinical trials with immune therapeutics. Annals of translational medicine 2016, 4(14).

36. Khan H, Gucalp R, Shapira I: Evolving concepts: Immunity in oncology from targets to treatments. Journal of Oncology 2015, 2015.

37. Landi L, Cappuzzo F: Experience with erlotinib in the treatment of non-small cell lung cancer. Therapeutic advances in respiratory disease 2015, 9(4):146-163.

38. Jones E, Mikropoulos C, Ahmed M: The future of immunotherapy in the treatment of lung cancer. Lung Cancer Management 2015, 4(2):57-73.

39. Lizee G, Overwijk WW, Radvanyi L, Gao J, Sharma P, Hwu P: Harnessing the power of the immune system to target cancer. In., vol. 64; 2013: 71-90.

40. Reiss KA, Forde PM, Brahmer JR: Harnessing the power of the immune system via blockade of PD-1 and PD-L1: A promising new anticancer strategy. Immunotherapy 2014, 6(4):459-475.

41. Califano R, Kerr K, Morgan RD, Russo GL, Garassino M, Morgillo F, Rossi A: Immune Checkpoint Blockade: A New Era for Non-Small Cell Lung Cancer. Current oncology reports 2016, 18(9).

42. Helissey C, Champiat S, Soria JC: Immune checkpoint inhibitors in advanced nonsmall cell lung cancer. Current opinion in oncology 2015, 27(2):108-117.

43. Kourie HR, Klastersky J: Immune checkpoint inhibitors side effects and management. Immunotherapy 2016, 8(7):799-807.

44. Sharma P, Allison JP: Immune checkpoint targeting in cancer therapy: Toward combination strategies with curative potential. Cell 2015, 161(2):205-214.

45. V an Vugt M, De Greef R, Freshwater T, Mangin E, V an Aarle F, Kondic A: Immunogenicity of pembrolizumab (pembro) in patients (pts) with advanced melanoma (MEL) and non-small cell lung cancer (NSCLC): Pooled results from KEYNOTE-001, 002, 006, and 010. Journal of Clinical Oncology 2016, 34.

46. Harvey RD: Immunologic and clinical effects of targeting PD-1 in lung cancer. Clinical Pharmacology and Therapeutics 2014, 96(2):214-223.

47. Reck M, Paz-Ares L: Immunologic checkpoint blockade in lung cancer. Seminars in oncology 2015, 42(3):402-417.

48. Momtaz P, Postow MA: Immunologic checkpoints in cancer therapy: Focus on the programmed death-1 (PD-1) receptor pathway. Pharmacogenomics and Personalized Medicine 2014, 7:357-365.

49. Marciniec M, Nowak A, Filip A: Immunomodulatory antibodies in cancer therapy. Nowotwory 2015, 65(1):42-47.

50. Honeychurch J, Cheadle EJ, Dovedi SJ, Illidge TM: Immuno-regulatory antibodies for the treatment of cancer. Expert opinion on biological therapy 2015, 15(6):787-801.

51. Dempke WCM, Sellmann L, Fenchel K, Edvardsen K: Immunotherapies for NSCLC: Are we cutting the gordian helix? Anticancer Research 2015, 35(11):5745-5757.

52. Morrissey K, Yuraszeck T, Li CC, Zhang Y, Kasichayanula S: Immunotherapy and Novel Combinations in Oncology: Current Landscape, Challenges, and Opportunities. Clinical and Translational Science 2016, 9(2):89-104.

53. Peters S: Immunotherapy for lung cancer. Annals of Oncology 2015, 26:vii7.

54. Reinmuth N, Reck M: Immunotherapy for Lung Cancer. Oncology Research and Treatment 2016, 39(6):360-368.

55. Mostafa AA, Morris DG: Immunotherapy for lung cancer: Has it finally arrived? Frontiers in Oncology 2014, 4(OCT).

56. Declerck S, Vansteenkiste J: Immunotherapy for lung cancer: Ongoing clinical trials. Future Oncology 2014, 10(1):91-105.

57. Freeman S: Immunotherapy induces striking responses in NSCLC. Oncology Report 2013(OCT):12.

58. Whitehurst M, Chiappori A: Immunotherapy treatments for small-cell lung cancer: Past, present and future. Lung Cancer Management 2013, 2(6):517-525.

59. Rolfo C, Sortino G, Smits E, Passiglia F, Bronte G, Castiglia M, Russo A, Santos ES, Janssens A, Pauwels P et al: Immunotherapy: Is a minor god yet in the pantheon of treatments for lung cancer? Expert review of anticancer therapy 2014, 14(10):1173-1187.

60. Choudhury N, Nakamura Y: Importance of immunopharmacogenomics in cancer treatment: Patient selection and monitoring for immune checkpoint antibodies. Cancer Science 2016, 107(2):107-115.

61. Shu CA, Rizvi NA: Into the clinic with nivolumab and pembrolizumab. The oncologist 2016, 21(5):527-528.

62. Rijavec E, Genova C, Barletta G, Burrafato G, Biello F, Dal Bello MG, Coco S, Truini A, Alama A, Boccardo F et al: Ipilimumab in non-small cell lung cancer and small-cell lung cancer: New knowledge on a new therapeutic strategy. Expert opinion on biological therapy 2014, 14(7):1007-1017.

63. Romero D: Lung cancer: Nivolumab-an effective second-line treatment for NSCLC. Nature Reviews Clinical Oncology 2015, 12(12):685.

64. Pugliese SB, Neal JW, Kwong BY: Management of Dermatologic Complications of Lung Cancer Therapies. Current treatment options in oncology 2015, 16(10).

65. Dadu R, Zobniw C, Diab A: Managing adverse events with immune checkpoint agents. Cancer Journal (United States) 2016, 22(2):121-129.

66. Topalian SL, Taube JM, Anders RA, Pardoll DM: Mechanism-driven biomarkers to guide immune checkpoint blockade in cancer therapy. Nature Reviews Cancer 2016, 16(5):275-287.

67. Scarpace SL: Metastatic squamous cell non-small-cell lung cancer (NSCLC): Disrupting the drug treatment paradigm with immunotherapies. Drugs in Context 2015, 4.

68. Li Y, Li F, Jiang F, Lv X, Zhang R, Lu A, Zhang G: A mini-review for cancer immunotherapy: Molecular understanding of PD-1/ PD-L1 pathway & translational blockade of immune checkpoints. International Journal of Molecular Sciences 2016, 17(7).

69. Gangadhar TC, Vonderheide RH: Mitigating the toxic effects of anticancer immunotherapy. Nature Reviews Clinical Oncology 2014, 11(2):91-99.

70. Ahamadi M, Li C, Elassaiss-Schaap J, De Greef R, Kondic A: Model-based pooled analysis of exposure and safety of pembrolizumab with advanced melanoma and non-small cell lung carcinoma (NSCLC). Journal of Pharmacokinetics and Pharmacodynamics 2015, 42(1):S66.

71. Shtivelman E, Hensing T, Simon GR, Dennis PA, Otterson GA, Bueno R, Salgia R: Molecular pathways and therapeutic targets in lung cancer. Oncotarget 2014, 5(6):1392-1433.

72. Carnio S, Novello S, Bironzo P, Scagliotti GV: Moving from histological subtyping to molecular characterization: New treatment opportunities in advanced non-small-cell lung cancer. Expert review of anticancer therapy 2014, 14(12):1495-1513.

73. Franks HA, Wang Q, Patel PM: New anticancer immunotherapies. Anticancer Research 2012, 32(7):2439-2454.

74. Zago G, Muller M, Van Den Heuvel M, Baas P: New targeted treatments for non-small-cell lung cancer- Role of nivolumab. Biologics: Targets and Therapy 2016, 10:103-117.

75. Antoniu S, Ulmeanu R: Nivolumab for advanced non-small cell lung cancer: An immunologically-mediated tumor checkout. Annals of translational medicine 2016, 4(10).

76. Wendling P: Nivolumab transforms practice for advanced, refractory nonsquamous NSCLC. Oncology Report 2015, 11(6):22-23.

77. Wang L, Zhang ZY: Nivolumab: a new PD-1 inhibitor. Chinese Journal of New Drugs 2016, 25(9):961-963.

78. Moreira Da Silva R: Nivolumab: Anti-PD-1 monoclonal antibody cancer immunotherapy. Drugs of the Future 2014, 39(1):15-24.

79. Perez-Gracia JL, Labiano S, Rodriguez-Ruiz ME, Sanmamed MF, Melero I: Orchestrating immune check-point blockade for cancer immunotherapy in combinations. Current Opinion in Immunology 2014, 27(1):89-97.

80. McDermott DF, Atkins MB: PD-1 as a potential target in cancer therapy. Cancer Medicine 2013, 2(5):662-673.

81. Dolan DE, Gupta S: PD-1 pathway inhibitors: Changing the landscape of cancer immunotherapy. Cancer Control 2014, 21(3):231-237.

82. Ferris R: PD-1 targeting in cancer immunotherapy. Cancer 2013, 119(23):E1-E3.

83. Jing W, Li M, Zhang Y, Teng F, Han A, Kong L, Zhu H: PD-1/PD-l1 blockades in non-small-cell lung cancer therapy. OncoTargets and therapy 2016, 9:489-502.

84. Sunshine J, Taube JM: PD-1/PD-L1 inhibitors. Current Opinion in Pharmacology 2015, 23:32-38.

85. Brahmer JR: PD-1-targeted immunotherapy: Recent clinical findings. Clinical Advances in Hematology and Oncology 2012, 10(10):674-675.

86. Ibrahim R, Stewart R, Shalabi A: PD-L1 Blockade for Cancer Treatment: MEDI4736. Seminars in oncology 2015, 42(3):474-483.

87. Dales MJM: PD-L1 blocker shrinks tumors with modest adverse events. Oncology Report 2013(JUN):13.

88. Fusi A, Festino L, Botti G, Masucci G, Melero I, Lorigan P, Ascierto PA: PD-L1 expression as a potential predictive biomarker. The Lancet Oncology 2015, 16(13):1285-1287.

89. Dang TO, Ogunniyi A, Barbee MS, Drilon A: Pembrolizumab for the treatment of PD-L1 positive advanced or metastatic non-small cell lung cancer. Expert review of anticancer therapy 2016, 16(1):13-20.

90. Postel-vinay S, Soria JC: Phase I trials in oncology: A new era has started. Annals of Oncology 2015, 26(1):7-9.

91. Comer B: Pipeline report. Drug Topics 2013, 157(2).

92. Herbst RS, Soria JC, Kowanetz M, Fine GD, Hamid O, Gordon MS, Sosman JA, McDermott DF, Powderly JD, Gettinger SN et al: Predictive correlates of response to the anti-PD-L1 antibody MPDL3280A in cancer patients. Nature 2014, 515(7528):563-567.

93. Schalper KA, Venur VA, Velcheti V: Programmed death-1/programmed death-1 ligand axis as a therapeutic target in oncology: Current insights. Journal of Receptor, Ligand and Channel Research 2015, 8:1-7.

94. Zielinski C, Knapp S, Mascaux C, Hirsch F: Rationale for targeting the immune system through checkpoint molecule blockade in the treatment of non-small-cell lung cancer. Annals of Oncology 2013, 24(5):1170-1179.

95. Genova C, Rijavec E, Grossi F: Recent advances in squamous non-small cell lung cancer: Evidence beyond predictive biomarkers. Expert review of anticancer therapy 2016, 16(1):1-4.

96. Yang LL, Wu YL: Recent advances of immunotherapy in lung cancer: Anti-programmed cell death-1/programmed death ligand-1 antibodies. Lung Cancer Management 2014, 3(2):175-190.

97. Butler T, Maravent S, Boisselle J, Valdes J, Fellner C: A review of 2014 cancer drug approvals,with a look at 2015 and beyond. P and T 2015, 40(3):191-205.

98. Santabarbara G, Maione P, Rossi A, Palazzolo G, Gridelli C: The role of pembrolizumab in the treatment of advanced non-small cell lung cancer. Annals of translational medicine 2016, 4(11).

99. Prowell TM, Theoret MR, Pazdur R: Seamless oncology-drug development. New England Journal of Medicine 2016, 374(21):2001-2003.

100. Kanai O, Fujita K, Okamura M, Nakatani K, Mio T: Severe exacerbation or manifestation of primary disease related to nivolumab in non-small-cell lung cancer patients with poor performance status or brain metastases. Annals of Oncology 2016, 27(7):1354-1356.

101. Seetharamu N: The state of the art in non-small cell lung cancer immunotherapy. Seminars in Thoracic and Cardiovascular Surgery 2014, 26(1):26-35.

102. Cohen JV, Kluger HM: Systemic immunotherapy for the treatment of brain metastases. Frontiers in Oncology 2016, 6(MAR).

103. Murray N: Systemic therapy of extensive stage small cell lung cancer (SCLC): Contrasting therapeutic principles for SCLC and non-small cell lung cancer (NSCLC) in 2015. Journal of Thoracic Oncology 2015, 10(9):S164-S165.

104. Ott PA, Stephen Hodi F: T cell modulation: Anti-PD-1 antibodies for the treatment of cancer. In., vol. 87; 2015: 231-244.

105. Afghahi A, Sledge GW: Targeted Therapy for Cancer in the Genomic Era. Cancer Journal (United States) 2015, 21(4):294-298.

106. Rangachari D, Brahmer JR: Targeting the immune system in the treatment of non-small-cell lung cancer. Current treatment options in oncology 2013, 14(4):580-594.

107. Naidoo J, Page DB, Li BT, Connell LC, Schindler K, Lacouture ME, Postow MA, Wolchok JD: Toxicities of the anti-PD-1 and anti-PD-L1 immune checkpoint antibodies. Annals of Oncology 2015, 26(12):2375-2391.

108. Van Elsas A, Van Eenennaam H, Haanen JB: Translating pembrolizumab to clinical practice: Speak immunology and learn fast! Clinical Cancer Research 2015, 21(19):4251-4253.

109. Zhou C, Jiang T: Treatment of advanced SCLC including second line. Journal of Thoracic Oncology 2015, 10(9):S146.

110. Brahmer J: Treatment targeting PD1/PDL1 and toxicity. Journal of Thoracic Oncology 2013, 8:S53-S54.

111. Aranda F, Vacchelli E, Eggermont A, Galon J, Fridman WH, Zitvogel L, Kroemer G, Galluzzi L: Trial Watch: Immunostimulatory monoclonal antibodies in cancer therapy. OncoImmunology 2014, 3(2).

112. Vacchelli E, Eggermont A, Galon J, Sautès-Fridman C, Zitvogel L, Kroemer G, Galluzzi L: Trial watch: Monoclonal antibodies in cancer therapy. OncoImmunology 2013, 2(1).

113. Stinchcombe TE: Unmet needs in squamous cell carcinoma of the lung: potential role for immunotherapy. Medical Oncology 2014, 31(5):1-9.

114. Reck M: What future opportunities may immuno-oncology provide for improving the treatment of patients with lung cancer? Annals of Oncology 2012, 23(SUPPL.8):viii28-viii34.

115. Ascierto PA, Marincola FM: What have we learned from cancer immunotherapy in the last 3 years? Journal of Translational Medicine 2014, 12(1).

116. Ascierto PA: What we learned from immunotherapy in previous years. Molecular Cancer Therapeutics 2015, 14(12).

117. Comer B: What's in the pipeline for 2014? Drug Topics 2014(FEB).

118. Voena C, Chiarle R: Advances in cancer immunology and cancer immunotherapy. Discovery medicine 2016, 21(114):125-133.

119. Sgambato A, Casaluce F, Sacco PC, Palazzolo G, Maione P, Rossi A, Ciardiello F, Gridelli C: Anti PD-1 and PDL-1 Immunotherapy in the Treatment of Advanced Non- Small Cell Lung Cancer (NSCLC): A Review on Toxicity Profile and its Management. Current drug safety 2016, 11(1):62-68.

120. Tanaka Y, Okamura H: [Anti-PD-1 antibody: basics and clinical application]. Gan to kagaku ryoho Cancer & chemotherapy 2013, 40(9):1145-1149.

121. Hamid O, Carvajal RD: Anti-programmed death-1 and anti-programmed death-ligand 1 antibodies in cancer therapy. Expert opinion on biological therapy 2013, 13(6):847-861.

122. Heinzerling L, Ott PA, Hodi FS, Husain AN, Tajmir-Riahi A, Tawbi H, Pauschinger M, Gajewski TF, Lipson EJ, Luke JJ: Cardiotoxicity associated with CTLA4 and PD1 blocking immunotherapy. Journal for immunotherapy of cancer 2016, 4:50.

123. Lu J, Lee-Gabel L, Nadeau MC, Ferencz TM, Soefje SA: Clinical evaluation of compounds targeting PD-1/PD-L1 pathway for cancer immunotherapy. Journal of oncology pharmacy practice : official publication of the International Society of Oncology Pharmacy Practitioners 2015, 21(6):451-467.

124. Barbee MS, Ogunniyi A, Horvat TZ, Dang TO: Current status and future directions of the immune checkpoint inhibitors ipilimumab, pembrolizumab, and nivolumab in oncology. The Annals of pharmacotherapy 2015, 49(8):907-937.

125. Ma W, Gilligan BM, Yuan J, Li T: Current status and perspectives in translational biomarker research for PD-1/PD-L1 immune checkpoint blockade therapy. Journal of hematology & oncology 2016, 9(1):47.

126. Langer CJ: Emerging immunotherapies in the treatment of non-small cell lung cancer (NSCLC): the role of immune checkpoint inhibitors. American journal of clinical oncology 2015, 38(4):422-430.

127. Gridelli C, Besse B, Brahmer JR, Crino L, Felip E, de Marinis F: The Evolving Role of Nivolumab in Non-Small-Cell Lung Cancer for Second-Line Treatment: A New Cornerstone for Our Treatment Algorithms. Results From an International Experts Panel Meeting of the Italian Association of Thoracic Oncology. Clinical lung cancer 2016, 17(3):161-168.

128. Chow LQ: Exploring novel immune-related toxicities and endpoints with immune-checkpoint inhibitors in non-small cell lung cancer. American Society of Clinical Oncology educational book / ASCO American Society of Clinical Oncology Meeting 2013.

129. Somasundaram A, Socinski MA, Villaruz LC: Immune checkpoint blockade in lung cancer. Discovery medicine 2016, 22(119):55-65.

130. Villadolid J, Amin A: Immune checkpoint inhibitors in clinical practice: update on management of immune-related toxicities. Translational lung cancer research 2015, 4(5):560-575.

131. Seetharamu N, Budman DR, Sullivan KM: Immune checkpoint inhibitors in lung cancer: past, present and future. Future oncology (London, England) 2016, 12(9):1151-1163.

132. Elias R, Morales J, Rehman Y, Khurshid H: Immune Checkpoint Inhibitors in Older Adults. Current oncology reports 2016, 18(8):47.

133. La-Beck NM, Jean GW, Huynh C, Alzghari SK, Lowe DB: Immune Checkpoint Inhibitors: New Insights and Current Place in Cancer Therapy. Pharmacotherapy 2015, 35(10):963-976.

134. Miyauchi E, Inoue A: [Immune Checkpoint Therapy for Non-Small-Cell Lung Cancer]. Gan to kagaku ryoho Cancer & chemotherapy 2016, 43(6):666-671.

135. Pochesci A, Passaro A, Catania C, Noberasco C, Del Signore E, Spitaleri G, De Marinis F: [Immunotherapy in non-small cell lung cancer: evolution of knowledge and clinical advances]. Recenti progressi in medicina 2016, 107(4):186-192.

136. Guilleminault L, Carmier D, Heuze-Vourc'h N, Diot P, Pichon E: [Immunotherapy in non-small cell lung cancer: inhibition of PD1/PDL1 pathway]. Revue de pneumologie clinique 2015, 71(1):44-56.

137. Kobold S, Duewell P, Schnurr M, Subklewe M, Rothenfusser S, Endres S: Immunotherapy in Tumors. Deutsches Arzteblatt international 2015, 112(48):809-815.

138. Dudnik E, Yust-Katz S, Nechushtan H, Goldstein DA, Zer A, Flex D, Siegal T, Peled N: Intracranial response to nivolumab in NSCLC patients with untreated or progressing CNS metastases. Lung cancer (Amsterdam, Netherlands) 2016, 98:114-117.

139. Bakirhan K, Sharma J, Perez-Soler R, Cheng H: Medical Treatment in Elderly Patients with Non-Small Cell Lung Cancer. Current treatment options in oncology 2016, 17(3):13.

140. O'Kane GM, Labbe C, Doherty MK, Young K, Albaba H, Leighl NB: Monitoring and Management of Immune-Related Adverse Events Associated With Programmed Cell Death Protein-1 Axis Inhibitors in Lung Cancer. The oncologist 2016.

141. Davies M: New modalities of cancer treatment for NSCLC: focus on immunotherapy. Cancer management and research 2014, 6:63-75.

142. Gunturi A, McDermott DF: Nivolumab for the treatment of cancer. Expert opinion on investigational drugs 2015, 24(2):253-260.

143. Guibert N, Mazieres J: Nivolumab for treating non-small cell lung cancer. Expert opinion on biological therapy 2015, 15(12):1789-1797.

144. Lim JS, Soo RA: Nivolumab in the treatment of metastatic squamous non-small cell lung cancer: a review of the evidence. Therapeutic advances in respiratory disease 2016.

145. Flippot R, Fallet V, Besse B, Massard C, Wislez M, Vignot S: [Nivolumab, a new hope in non-small cell lung cancer]. Bulletin du cancer 2015, 102(12):1046-1052.

146. Keating GM: Nivolumab: A Review in Advanced Nonsquamous Non-Small Cell Lung Cancer. Drugs 2016, 76(9):969-978.

147. Keating GM: Nivolumab: a review in advanced squamous non-small cell lung cancer. Drugs 2015, 75(16):1925-1934.

148. Brahmer JR, Hammers H, Lipson EJ: Nivolumab: targeting PD-1 to bolster antitumor immunity. Future oncology (London, England) 2015, 11(9):1307-1326.

149. Medina PJ, Adams VR: PD-1 Pathway Inhibitors: Immuno-Oncology Agents for Restoring Antitumor Immune Responses. Pharmacotherapy 2016, 36(3):317-334.

150. Adam J, Planchard D, Marabelle A, Soria JC, Scoazec JY, Lantuejoul S: [PD-L1 expression: An emerging biomarker in non-small cell lung cancer]. Annales de pathologie 2016, 36(1):94-102.

151. Kwok G, Yau TC, Chiu JW, Tse E, Kwong YL: Pembrolizumab (Keytruda). Human vaccines & immunotherapeutics 2016:0.

152. McDermott J, Jimeno A: Pembrolizumab: PD-1 inhibition as a therapeutic strategy in cancer. Drugs of today (Barcelona, Spain : 1998) 2015, 51(1):7-20.

153. Melosky B, Chu Q, Juergens R, Leighl N, McLeod D, Hirsh V: Pointed Progress in Second-Line Advanced Non-Small-Cell Lung Cancer: The Rapidly Evolving Field of Checkpoint Inhibition. Journal of clinical oncology : official journal of the American Society of Clinical Oncology 2016, 34(14):1676-1688.

154. Weber JS: Practical management of immune-related adverse events from immune checkpoint protein antibodies for the oncologist. American Society of Clinical Oncology educational book / ASCO American Society of Clinical Oncology Meeting 2012:174-177.

155. Ang YL, Lim JS, Soo RA: Profile of nivolumab in the treatment of metastatic squamous non-small-cell lung cancer. OncoTargets and therapy 2016, 9:3187-3195.

156. Programmed Death-1 Inhibition in Cancer With a Focus on Non-Small Cell Lung Cancer: Rationale, Nursing Implications, and Patient Management Strategies. Clinical journal of oncology nursing 2016, 20(3):319-326.

157. Brahmer JR, Tykodi SS, Chow LQ, Hwu WJ, Topalian SL, Hwu P, Drake CG, Camacho LH, Kauh J, Odunsi K et al: Safety and activity of anti-PD-L1 antibody in patients with advanced cancer. The New England journal of medicine 2012, 366(26):2455-2465.

158. Tanvetyanon T, Creelan BC, Antonia SJ: The safety and efficacy of nivolumab in advanced (metastatic) non-small cell lung cancer. Expert review of anticancer therapy 2016, 16(9):903-910.

159. Topalian SL, Hodi FS, Brahmer JR, Gettinger SN, Smith DC, McDermott DF, Powderly JD, Carvajal RD, Sosman JA, Atkins MB et al: Safety, activity, and immune correlates of anti-PD-1 antibody in cancer. The New England journal of medicine 2012, 366(26):2443-2454.

160. Kourie HR, Klastersky JA: Side-effects of checkpoint inhibitor-based combination therapy. Current opinion in oncology 2016, 28(4):306-313.

161. Zimmerman MP, Mehr SR: Targeted programmed cell death in lung cancer treatment. The American journal of managed care 2014, 20(5 Spec No.):E3.

162. Feld E, Horn L: Targeting PD-L1 for non-small-cell lung cancer. Immunotherapy 2016, 8(6):747-758.

163. Moro-Sibilot D, Ferrer L, Levra MG, Toffart AC: TREATMENT OF NSCLC WITH NIVOLUMAB. Bulletin du cancer 2016, 103(6 Suppl 1):S12-15.

164. Wong AC, Ma B: An update on the pharmacodynamics, pharmacokinetics, safety and clinical efficacy of nivolumab in the treatment of solid cancers. Expert opinion on drug metabolism & toxicology 2016:1-7.

165. Shimanovsky A, Dasanu CA: Updates on immunotherapy in non-small cell lung cancer. Expert opinion on biological therapy 2014, 14(4):411-418.

166. Nivolumab (Opdivo) for metastatic melanoma and metastatic NSCLC. In: Medical letter on drugs and therapeutics. vol. 57; 2015: 85-87.

167. Giri A, Walia SS, Gajra A: Clinical Trials Investigating Immune Checkpoint Inhibitors in Non-Small-Cell Lung Cancer. Reviews on recent clinical trials 2016.

168. Rounds A, Kolesar J: Nivolumab for second-line treatment of metastatic squamous non-small-cell lung cancer. American journal of health-system pharmacy : AJHP : official journal of the American Society of Health-System Pharmacists 2015, 72(21):1851-1855.

**Meta analysis and systematic review: 12**

1. Des Guetz G, Landre T, Nicolas P, Vergnenegre A, Chouaid C: Anti PD-1 (nivolumab, pembrolizumab) or anti PD-L1 (atezolizumab) versus docetaxel for previously treated patients with advanced NSCLC: A meta-analysis. Journal of Clinical Oncology 2016, 34.

2. Nishijima TF, Shachar SS, Muss HB: Comparison of treatment-related fatigue and tolerability between PD-1 immune checkpoint inhibitors and chemotherapy: A meta-analysis. Journal of Clinical Oncology 2016, 34.

3. Pillai RN, Behera M, Owonikoko TK, Kamphorst AO, Pakkala S, Belani CP, Khuri FR, Ahmed R, Ramalingam SS: Evaluation of toxicity profile of PD-1 versus PD-L1 inhibitors in non-small cell lung cancer (NSCLC). Journal of Clinical Oncology 2016, 34.

4. Peng L, Zhao Q, Ye X, Wang Y, Mou H: Incidence and risk of pneumonitis associated with nivolumab and pembrolizumab in patients with cancer: A meta-analysis of clinical trials. Journal of Clinical Oncology 2016, 34.

5. Passiglia F, Bronte G, Rizzo S, Galvano A, Sortino G, Musso E, Listì A, Barraco N, Castiglia M, Calò V et al: PD-L1 expression as predictive biomarker in patients with NSCLC: A pooled analysis. Journal of Thoracic Oncology 2015, 10(9):S232-S233.

6. Aguiar Jr P, Santoro I, Tadokoro H, Filardi BA, Lopes GL, Picon FS, Noia Barreto CM, Muniz PC, Borges AM, Oliveira P et al: Polled analysis of nivolumab for the treatment of advanced non-small cell lung cancer and the role of PD-L1 as a biomarker. European Journal of Cancer 2015, 51:S624.

7. Aguiar PN, Santoro IL, Tadokoro H, De Lima Lopes G, Filardi BA, Oliveira P, Castelo-Branco P, Mountzios G, De Mello RA: A pooled analysis of nivolumab for the treatment of advanced non-small-cell lung cancer and the role of PD-L1 as a predictive biomarker. Immunotherapy 2016, 8(9):1011-1019.

8. Abdel-Rahman O, ElHalawani H, Fouad M: Risk of cutaneous toxicities in patients with solid tumors treated with immune checkpoint inhibitors: A meta-analysis. Future Oncology 2015, 11(17):2471-2484.

9. Abdel-Rahman O, Fouad M: Risk of pneumonitis in cancer patients treated with immune checkpoint inhibitors: A meta-analysis. Therapeutic advances in respiratory disease 2016, 10(3):183-193.

10. Zhou GW, Xiong Y, Chen S, Xia F, Li Q, Hu J: Anti-PD-1/PD-L1 antibody therapy for pretreated advanced nonsmall-cell lung cancer: A meta-analysis of randomized clinical trials. Medicine 2016, 95(35):e4611.

11. Jia M, Feng W, Kang S, Zhang Y, Shen J, He J, Jiang L, Wang W, Guo Z, Peng G et al: Evaluation of the efficacy and safety of anti-PD-1 and anti-PD-L1 antibody in the treatment of non-small cell lung cancer (NSCLC): a meta-analysis. Journal of thoracic disease 2015, 7(3):455-461.

12. Passiglia F, Bronte G, Bazan V, Natoli C, Rizzo S, Galvano A, Listi A, Cicero G, Rolfo C, Santini D et al: PD-L1 expression as predictive biomarker in patients with NSCLC: a pooled analysis. Oncotarget 2016, 7(15):19738-19747.

**Other tumors: 95**

1. Bickel A, Koneth I, Enzler-Tschudy A, Neuweiler J, Flatz L, Fruh M: Pembrolizumab-associated minimal change disease in a patient with malignant pleural mesothelioma. BMC cancer 2016, 16:656.

2. Bosch-Barrera J, Holguin F, Baldo X, Rubio M, Porta R, Fuentes R, Teixido C, Ramirez JL, Ferran N, Sebastian F et al: Neoadjuvant Chemoradiotherapy Treatment for a Classic Biphasic Pulmonary Blastoma with High PD-L1 Expression. Anticancer research 2015, 35(9):4871-4875.

3. Clavijo-Salomon MA, Ramos RN, Crippa A, Pizzo CR, Bergami-Santos PC, Barbuto JA: Monocyte-derived dendritic cells reflect the immune functional status of a chromophobe renal cell carcinoma patient: could it be a general phenomenon? Cancer immunology, immunotherapy : CII 2015, 64(2):161-171.

4. Cohen PR, Kato S, Goodman AM, Ikeda S, Kurzrock R: Appearance of New Cutaneous Superficial Basal Cell Carcinomas during Successful Nivolumab Treatment of Refractory Metastatic Disease: Implications for Immunotherapy in Early Versus Late Disease. International journal of molecular sciences 2017, 18(8).

5. Crescenzi A, Taffon C, Donati M, Guarino MP, Valeri S, Coppola R: PD-L1/PD-1 check-point in gastric carcinoma with lymphoid stroma case report with immunochemical study. Medicine 2017, 96(7):e5730.

6. Czink E, Kloor M, Goeppert B, Frohling S, Uhrig S, Weber TF, Meinel J, Sutter C, Weiss KH, Schirmacher P et al: Successful immune checkpoint blockade in a patient with advanced stage microsatellite-unstable biliary tract cancer. Cold Spring Harbor molecular case studies 2017, 3(5).

7. Davick JJ, Wick MR, Gru AA: Development of a biclonal cutaneous T-cell lymphoproliferative process during treatment with immune checkpoint inhibitors for metastatic melanoma. Melanoma research 2017, 27(4):383-386.

8. de Velasco G, Krajewski KM, Albiges L, Awad MM, Bellmunt J, Hodi FS, Choueiri TK: Radiologic Heterogeneity in Responses to Anti-PD-1/PD-L1 Therapy in Metastatic Renal Cell Carcinoma. Cancer immunology research 2016, 4(1):12-17.

9. Diem S, Keller F, Ruesch R, Maillard SA, Speiser DE, Dummer R, Siano M, Urner-Bloch U, Goldinger SM, Flatz L: Pembrolizumab-triggered Uveitis: An Additional Surrogate Marker for Responders in Melanoma Immunotherapy? Journal of immunotherapy (Hagerstown, Md : 1997) 2016, 39(9):379-382.

10. Escandon J, Peacock S, Trabolsi A, Thomas DB, Layka A, Lutzky J: Interstitial nephritis in melanoma patients secondary to PD-1 checkpoint inhibitor. Journal for immunotherapy of cancer 2017, 5:3.

11. Foran AE, Nadel HR, Lee AF, Savage KJ, Deyell RJ: Nivolumab in the Treatment of Refractory Pediatric Hodgkin Lymphoma. Journal of pediatric hematology/oncology 2017, 39(5):e263-e266.

12. Freites-Martinez A, Kwong BY, Rieger KE, Coit DG, Colevas AD, Lacouture ME: Eruptive Keratoacanthomas Associated With Pembrolizumab Therapy. JAMA dermatology 2017, 153(7):694-697.

13. Fujimura T, Furudate S, Kakizaki A, Kambayashi Y, Haga T, Hashimoto A, Aiba S: Contact immunotherapy enhances the therapeutic effects of nivolumab in treating in-transit melanoma: Two cases reports. The Journal of dermatology 2016, 43(6):686-689.

14. Galsky MD, Domingo-Domenech J, Sfakianos JP, Ferket BS: Definitive Management of Primary Bladder Tumors in the Context of Metastatic Disease: Who, How, When, and Why? Journal of clinical oncology : official journal of the American Society of Clinical Oncology 2016, 34(29):3495-3498.

15. Gambichler T, Strutzmann S, Tannapfel A, Susok L: Paraneoplastic acral vascular syndrome in a patient with metastatic melanoma under immune checkpoint blockade. BMC cancer 2017, 17(1):327.

16. Gao J, Behdad A, Ji P, Wolniak KL, Frankfurt O, Chen YH: EBV-negative aggressive NK-cell leukemia/lymphoma: a clinical and pathological study from a single institution. Modern pathology : an official journal of the United States and Canadian Academy of Pathology, Inc 2017, 30(8):1100-1115.

17. Gauci ML, Laly P, Vidal-Trecan T, Baroudjian B, Gottlieb J, Madjlessi-Ezra N, Da Meda L, Madelaine-Chambrin I, Bagot M, Basset-Seguin N et al: Autoimmune diabetes induced by PD-1 inhibitor-retrospective analysis and pathogenesis: a case report and literature review. Cancer immunology, immunotherapy : CII 2017, 66(11):1399-1410.

18. George S, Miao D, Demetri GD, Adeegbe D, Rodig SJ, Shukla S, Lipschitz M, Amin-Mansour A, Raut CP, Carter SL et al: Loss of PTEN Is Associated with Resistance to Anti-PD-1 Checkpoint Blockade Therapy in Metastatic Uterine Leiomyosarcoma. Immunity 2017, 46(2):197-204.

19. Gong J, Wang C, Lee PP, Chu P, Fakih M: Response to PD-1 Blockade in Microsatellite Stable Metastatic Colorectal Cancer Harboring a POLE Mutation. Journal of the National Comprehensive Cancer Network : JNCCN 2017, 15(2):142-147.

20. Hanna KS: A Rare Case of Pembrolizumab-Induced Uveitis in a Patient with Metastatic Melanoma. Pharmacotherapy 2016, 36(11):e183-e188.

21. Harlin H, Kuna TV, Peterson AC, Meng Y, Gajewski TF: Tumor progression despite massive influx of activated CD8(+) T cells in a patient with malignant melanoma ascites. Cancer immunology, immunotherapy : CII 2006, 55(10):1185-1197.

22. Hickmott L, De La Pena H, Turner H, Ahmed F, Protheroe A, Grossman A, Gupta A: Anti-PD-L1 atezolizumab-Induced Autoimmune Diabetes: a Case Report and Review of the Literature. Targeted oncology 2017, 12(2):235-241.

23. Hidalgo-Lopez JE, Kanagal-Shamanna R, Quesada AE, Thakral B, Hu Z, Mitsuhashi T, Yabe M, Garcia-Manero G, Bueso-Ramos CE: Progress in Myelodysplastic Syndromes: Clinicopathologic Correlations and Immune Checkpoints. Clinical lymphoma, myeloma & leukemia 2017, 17s:S16-s25.

24. Johnson DB, Wallender EK, Cohen DN, Likhari SS, Zwerner JP, Powers JG, Shinn L, Kelley MC, Joseph RW, Sosman JA: Severe cutaneous and neurologic toxicity in melanoma patients during vemurafenib administration following anti-PD-1 therapy. Cancer immunology research 2013, 1(6):373-377.

25. Kohnke T, Krupka C, Tischer J, Knosel T, Subklewe M: Increase of PD-L1 expressing B-precursor ALL cells in a patient resistant to the CD19/CD3-bispecific T cell engager antibody blinatumomab. Journal of hematology & oncology 2015, 8:111.

26. Kulasinghe A, Perry C, Kenny L, Warkiani ME, Nelson C, Punyadeera C: PD-L1 expressing circulating tumour cells in head and neck cancers. BMC cancer 2017, 17(1):333.

27. Kumar V, Dave V, Harris J, Huang Y: Response of advanced stage recurrent lymphoepithelioma-like carcinoma to nivolumab. Immunotherapy 2017, 9(12):955-961.

28. Lu CS, Liu JH: Pneumonitis in cancer patients receiving anti-PD-1 and radiotherapies: Three case reports. Medicine 2017, 96(1):e5747.

29. Martini DJ, Lalani AA, Bosse D, Steinharter JA, Harshman LC, Hodi FS, Ott PA, Choueiri TK: Response to single agent PD-1 inhibitor after progression on previous PD-1/PD-L1 inhibitors: a case series. Journal for immunotherapy of cancer 2017, 5(1):66.

30. McCaughan GJ, Fulham MJ, Mahar A, Soper J, Hong AM, Stalley PD, Tattersall MH, Bhadri VA: Programmed cell death-1 blockade in recurrent disseminated Ewing sarcoma. Journal of hematology & oncology 2016, 9(1):48.

31. Nagasaka M, Zaki M, Kim H, Raza SN, Yoo G, Lin HS, Sukari A: PD1/PD-L1 inhibition as a potential radiosensitizer in head and neck squamous cell carcinoma: a case report. Journal for immunotherapy of cancer 2016, 4:83.

32. Nayak L, Iwamoto FM, LaCasce A, Mukundan S, Roemer MGM, Chapuy B, Armand P, Rodig SJ, Shipp MA: PD-1 blockade with nivolumab in relapsed/refractory primary central nervous system and testicular lymphoma. Blood 2017, 129(23):3071-3073.

33. Pollack SM, Lu H, Gnjatic S, Somaiah N, O'Malley RB, Jones RL, Hsu FJ, Ter Meulen J: First-in-Human Treatment With a Dendritic Cell-targeting Lentiviral Vector-expressing NY-ESO-1, LV305, Induces Deep, Durable Response in Refractory Metastatic Synovial Sarcoma Patient. Journal of immunotherapy (Hagerstown, Md : 1997) 2017, 40(8):302-306.

34. Putra J, Anderson TA, Roayaie S, Maeda M, Thung SN: Metastatic lymphoepithelioma-like hepatocellular carcinoma: a potential diagnostic pitfall and demonstration of PD-L1 expression. Annals of hepatology 2017, 16(1):157-159.

35. Shah S, Ward JE, Bao R, Hall CR, Brockstein BE, Luke JJ: Clinical Response of a Patient to Anti-PD-1 Immunotherapy and the Immune Landscape of Testicular Germ Cell Tumors. Cancer immunology research 2016, 4(11):903-909.

36. Simonelli M, Di Tommaso L, Baretti M, Santoro A: Pathological characterization of nivolumab-related liver injury in a patient with glioblastoma. Immunotherapy 2016, 8(12):1363-1369.

37. Sindhu S, Gimber LH, Cranmer L, McBride A, Kraft AS: Angiosarcoma treated successfully with anti-PD-1 therapy - a case report. Journal for immunotherapy of cancer 2017, 5(1):58.

38. Sodji Q, Klein K, Sravan K, Parikh J: Predictive role of PD-L1 expression in the response of renal Medullary carcinoma to PD-1 inhibition. Journal for immunotherapy of cancer 2017, 5(1):62.

39. Sponghini A, Patrucco F, Giorgione R, Farinelli P, Zottarelli F, Rondonotti D, Savoia P: Complete response to anti-PD-1 nivolumab in massive skin metastasis from melanoma: efficacy and tolerability in an elderly patient. Anti-cancer drugs 2017, 28(7):808-810.

40. Stevenson ML, Wang CQ, Abikhair M, Roudiani N, Felsen D, Krueger JG, Pavlick AC, Carucci JA: Expression of Programmed Cell Death Ligand in Cutaneous Squamous Cell Carcinoma and Treatment of Locally Advanced Disease With Pembrolizumab. JAMA dermatology 2017, 153(4):299-303.

41. Tetzlaff MT, Jazaeri AA, Torres-Cabala CA, Korivi BR, Landon GA, Nagarajan P, Choksi A, Chen L, Uemura M, Aung PP et al: Erythema nodosum-like panniculitis mimicking disease recurrence: A novel toxicity from immune checkpoint blockade therapy-Report of 2 patients. Journal of cutaneous pathology 2017, 44(12):1080-1086.

42. Trinidad C, Nelson KC, Glitza Oliva IC, Torres-Cabala CA, Nagarajan P, Tetzlaff MT, Ivan D, Hwu WJ, Prieto VG, Curry JL et al: Dermatologic toxicity from immune checkpoint blockade therapy with an interstitial granulomatous pattern. Journal of cutaneous pathology 2018.

43. Vandiver JW, Singer Z, Harshberger C: Severe Hyponatremia and Immune Nephritis Following an Initial Infusion of Nivolumab. Targeted oncology 2016, 11(4):553-556.

44. Vivar KL, Deschaine M, Messina J, Divine JM, Rabionet A, Patel N, Harrington MA, Seminario-Vidal L: Epidermal programmed cell death-ligand 1 expression in TEN associated with nivolumab therapy. Journal of cutaneous pathology 2017, 44(4):381-384.

45. Wang VE, Urisman A, Albacker L, Ali S, Miller V, Aggarwal R, Jablons D: Checkpoint inhibitor is active against large cell neuroendocrine carcinoma with high tumor mutation burden. Journal for immunotherapy of cancer 2017, 5(1):75.

46. Watanabe M, Yamamoto H, Hashida S, Soh J, Sugimoto S, Toyooka S, Miyoshi S: Primary pulmonary melanoma: a report of two cases. World journal of surgical oncology 2015, 13:274.

47. Yamauchi I, Sakane Y, Fukuda Y, Fujii T, Taura D, Hirata M, Hirota K, Ueda Y, Kanai Y, Yamashita Y et al: Clinical Features of Nivolumab-Induced Thyroiditis: A Case Series Study. Thyroid : official journal of the American Thyroid Association 2017, 27(7):894-901.

48. Zarbo A, Belum VR, Sibaud V, Oudard S, Postow MA, Hsieh JJ, Motzer RJ, Busam KJ, Lacouture ME: Immune-related alopecia (areata and universalis) in cancer patients receiving immune checkpoint inhibitors. The British journal of dermatology 2017, 176(6):1649-1652.

49. Gulley JL, Rajan A, Spigel DR, Iannotti N, Chandler J, Wong DJL, Leach J, Edenfield WJ, Wang D, Grote HJ et al: Avelumab for patients with previously treated metastatic or recurrent non-small-cell lung cancer (JAVELIN Solid Tumor): dose-expansion cohort of a multicentre, open-label, phase 1b trial. The Lancet Oncology 2017, 18(5):599-610.

50. Heery CR, O'Sullivan-Coyne G, Madan RA, Cordes L, Rajan A, Rauckhorst M, Lamping E, Oyelakin I, Marte JL, Lepone LM et al: Avelumab for metastatic or locally advanced previously treated solid tumours (JAVELIN Solid Tumor): a phase 1a, multicohort, dose-escalation trial. The Lancet Oncology 2017, 18(5):587-598.

51. Brahmer JR, Drake CG, Wollner I, Powderly JD, Picus J, Sharfman WH, Stankevich E, Pons A, Salay TM, McMiller TL et al: Phase I study of single-agent anti-programmed death-1 (MDX-1106) in refractory solid tumors: safety, clinical activity, pharmacodynamics, and immunologic correlates. Journal of clinical oncology : official journal of the American Society of Clinical Oncology 2010, 28(19):3167-3175.

52. Nct: An Exploratory Study to Investigate the Immunomodulatory Activity of Radiation Therapy (RT) in Combination With MK-3475 in Patients With Recurrent/Metastatic Head and Neck Cancer, Renal Cell Cancer, Melanoma and Non-Small Cell Lung Cancer. In: Clinicaltrialsgov [wwwclinicatrialsgov]. 2014.

53. Agrawal S, Feng Y, Roy A, Kollia G, Lestini B: Nivolumab dose selection: Challenges, opportunities and lessons learned for cancer immunotherapy. Journal for ImmunoTherapy of Cancer 2015, 3.

54. Arrondeau J, Boudou-Rouquette P, Goulvestre C, Huillard O, Kramkimel N, Chapron J, Tlemsani C, Jouinot A, Alifano M, Mansuet-Lupo A et al: Evaluation of baseline asymptomatic dysimmunity prevalence in cancer patients receiving monoclonal anti-PDI antibodies. Journal of Clinical Oncology 2016, 34.

55. Boudou-Rouquette P, Arrondeau J, Damotte D, Batteux F, Kramkimel N, Huillard O, Tlemsani C, Jouinot A, Chapron J, Dehghani L et al: A multidisciplinary team dedicated to the management of patients treated with PD1 inhibitors: The Cochin hospital experience. Journal of Clinical Oncology 2016, 34.

56. Brahmer JR, Topalian SL, Powderly J, Wollner I, Picus J, Drake CG, Stankevich E, Korman A, Pardoll D, Lowy I: Phase II experience with MDX-1106 (Ono-4538), an anti-PD-1 monoclonal antibody, in patients with selected refractory or relapsed malignancies. Journal of Clinical Oncology 2009, 27(15):3018.

57. Callahan MK, Bendell JC, Chan E, Morse M, Pillai RN, Bono P, Jaeger D, Jeffry Evans TR, Chau I, Calvo E et al: Phase I/II, open-label study of nivolumab (anti-PD-1; BMS-936558, ONO-4538) as monotherapy or combined with ipilimumab in advanced or metastatic solid tumors. Journal of Clinical Oncology 2014, 32(15).

58. Cappelli LC, Gutierrez AK, Baer AN, Albayda J, Manno RL, Haque U, Lipson EJ, Bleich KB, Shah AA, Naidoo J et al: Inflammatory arthritis and sicca syndrome induced by nivolumab and ipilimumab. Annals of the rheumatic diseases 2016.

59. Cha E, Wallin J, Kowanetz M: PD-L1 Inhibition With MPDL3280A for Solid Tumors. Seminars in Oncology 2015, 42(3):484-487.

60. Gangadhar TC, Hamid O, Smith DC, Bauer TM, Wasser JS, Luke JJ, Balmanoukian AS, Kaufman DR, Zhao Y, Maleski J et al: Preliminary results froma Phase I/II study of epacadostat (incb024360) in combination with pembrolizumab in patients with selected advanced cancers. Journal for ImmunoTherapy of Cancer 2015, 3.

61. Heery CR, Infante JR, Iannotti N, Kelly K, Nikolinakos P, Von Heydebreck A, Chin KM, Gulley JL: Phase I expansion cohort trial to investigate the safety and clinical activity of avelumab (MSB0010718C) in patients with metastatic or locally advanced solid tumors. Journal of Clinical Oncology 2015, 33(15).

62. Herbst RS, Bendell JC, Isambert N, Calvo E, Santana-Davila R, Cassier P, Perez-Gracia JL, Yang J, Rege J, Ferry D et al: A phase 1 study of ramucirumab (R) plus pembrolizumab (P) in patients (pts) with advanced gastric or gastroesophageal junction (G/GEJ) adenocarcinoma, non-small cell lung cancer (NSCLC), or urothelial carcinoma (UC): Phase 1a results. Journal of Clinical Oncology 2016, 34.

63. Herbst RS, Gordon MS, Fine GD, Sosman JA, Soria JC, Hamid O, Powderly JD, Burris HA, Mokatrin A, Kowanetz M et al: A study of MPDL3280A, an engineered PD-L1 antibody in patients with locally advanced or metastatic tumors. Journal of Clinical Oncology 2013, 31(15).

64. Hodi FS, Powles T, Cassier P, Kowanetz M, Herbst RS, Soria JC, Mokatrin A, Stroh M, Chen DS, Tabernero J: MPDL3280A (anti-PDL1): Clinical activity, safety and biomarkers of an engineered PD-L1 antibody in patients with locally advanced or metastatic tumors. European Journal of Cancer 2013, 49:S184.

65. Hodi FS, Topalian SL, Brahmer JR, McDermott DF, Smith DC, Gettinger S, Taube JM, Pardoll DM, Wigginton JM, Sznol M: Survival and long-term safety in patients (pts) with advanced solid tumors receiving nivolumab (anti-PD-1; BMS-936558; ONO-4538). European Journal of Cancer 2013, 49:S185.

66. Iasonos A, O'Quigley J: Clinical trials: Early phase clinical trials-are dose expansion cohorts needed? Nature Reviews Clinical Oncology 2015, 12(11):626-628.

67. Infante JR, Ahlers CM, Hodi FS, Postel-Vinay S, Schellens JHM, Heymach J, Autio KA, Barnette MS, Struemper H, Watmuff M et al: ENGAGE-1: A first in human study of the OX40 agonist GSK3174998 alone and in combination with pembrolizumab in patients with advanced solid tumors. Journal of Clinical Oncology 2016, 34.

68. Kanz BA, Pollack MLH, Eroglu Z, Anne R, Johnpulle N, Horn L, Rapisuwon S, Conry RM, Sosman JA, Puzanov I et al: Anti-PD-1 in patients with advanced malignancies and baseline organ dysfunction. Journal of Clinical Oncology 2016, 34.

69. Kelly K, Heery CR, Patel MR, Infante JR, Iannotti N, Leach JW, Wang D, Chandler JC, Arkenau HT, Taylor MH et al: Avelumab (MSB0010718C; anti-PD-LI) in patients with advanced cancer: Safety data from 1300 patients enrolled in the phase 1b JAVELIN trial. Journal of Clinical Oncology 2016, 34.

70. Kelly K, Patel MR, Infante JR, Iannotti N, Nikolinakos P, Leach J, Wang D, Chandler JC, Jerusalem GHM, Gurtler JS et al: Avelumab (MSB0010718C), an anti-PD-L1 antibody, in patients with metastatic or locally advanced solid tumors: Assessment of safety and tolerability in a phase I, open-label expansion study. Journal of Clinical Oncology 2015, 33(15).

71. Lutzky J, Antonia SJ, Blake-Haskins A, Li X, Robbins PB, Shalabi AM, Vasselli J, Ibrahim RA, Khleif S, Segal NH: A phase 1 study of MEDI4736, an anti-PD-L1 antibody, in patients with advanced solid tumors. Journal of Clinical Oncology 2014, 32(15).

72. Manrique CA, Afonso Afonso FJ, Rivera FV, Quintela ML, Vazquez-Estevez S, Calvo OF, Simo RV, Gonzalez JG, Lopez EP, Mata JG et al: An observational study of the efficacy and safety of nivolumab in pretreated patients with advanced non small cell lung cancer (NSCLC): A galician lung cancer group clinical practice. Journal of Clinical Oncology 2016, 34.

73. Mizugaki H, Yamamoto N, Murakami H, Kenmotsu H, Fujiwara Y, Ishida Y, Kawakami T, Takahashi T: Phase I dose-finding study of monotherapy with atezolizumab, an engineered immunoglobulin monoclonal antibody targeting PD-L1, in Japanese patients with advanced solid tumors. Investigational New Drugs 2016, 34(5):596-603.

74. Murakami H, Yamamoto N, Kenmotsu H, Takahashi T, Fujiwara Y, Mizugaki H, Ishida Y, Kawakami T: Phase I study of anti-PD-L1 antibody MPDL3280A in patients with advanced solid tumors. Annals of Oncology 2015, 26:vii98.

75. Naing A, Papadopoulos KP, Infante JR, Wong DJL, Autio KA, Ott PA, Falchook GS, Patel MR, Pant S, Patnaik A et al: Clinical activity and safety of pegylated human IL-10 (AM0010) in combination with anti-PD1. Journal of Clinical Oncology 2016, 34.

76. Patnaik A, Kang SP, Tolcher AW, Rasco DW, Papadopoulos KP, Beeram M, Drengler R, Chen C, Smith L, Perez C et al: Phase I study of MK-3475 (anti-PD-1 monoclonal antibody) in patients with advanced solid tumors. Journal of Clinical Oncology 2012, 30(15).

77. Ribas A, Chow LQ, Boyd JK, Long GV, Gorczyca M, Davis C, Pavlov D, Thall AD: Avelumab (MSB0010718C; anti-PD-LI) in combination with other cancer immunotherapies in patients with advanced malignancies: The phase 1b/2 JAVELIN medley study. Journal of Clinical Oncology 2016, 34.

78. Rizvi NA, Loo D, Baughman JE, Yun S, Chen F, Moore PA, Bonvini E, Vasselli JR, Wigginton JM, Cohen RB et al: A phase 1 study of enoblituzumab in combination with pembrolizumab in patients with advanced B7-H3-expressing cancers. Journal of Clinical Oncology 2016, 34.

79. Segal NH, Antonia SJ, Brahmer JR, Maio M, Blake-Haskins A, Li X, Vasselli J, Ibrahim RA, Lutzky J, Khleif S: Preliminary data from a multi-arm expansion study of MEDI4736, an anti-PD-L1 antibody. Journal of Clinical Oncology 2014, 32(15).

80. Segal NH, Hodi FS, Sanborn RE, Gajewski T, Wolchok JD, Urba WJ, Fox BA, Topalian SL, Pardoll DM, Kollia G et al: A phase I dose escalation and cohort expansion study of lirilumab (anti-KIR; BMS-986015) in combination with nivolumab (anti-PD-1; BMS-936558, ONO-4538) in advanced solid tumors. Journal of Clinical Oncology 2014, 32(15).

81. Shimizu T, Seto T, Hirai F, Takenoyama M, Nosaki K, Tsurutani J, Kaneda H, Iwasa T, Kawakami H, Noguchi K et al: Phase 1 study of pembrolizumab (MK-3475; anti-PD-1 monoclonal antibody) in Japanese patients with advanced solid tumors. Invest New Drugs 2016, 34(3):347-354.

82. Shitara K, Yamada Y, Yoh K, Naito Y, Iwasa S, Yamamoto N, Von Heydebreck A, Achiwa H, Doi T: Phase I, open-label, multi-ascending dose trial of avelumab (MSB0010718C), an anti-PD-L1 monoclonal antibody, in Japanese patients with advanced solid tumors. Journal of Clinical Oncology 2015, 33(15).

83. Singh H, Kim G, Maher VE, Beaver JA, Pai-Scherf LH, Balasubramaniam S, Theoret MR, Blumenthal GM, Pazdur R: FDA subset analysis of the safety of nivolumab in elderly patients with advanced cancers. Journal of Clinical Oncology 2016, 34.

84. Sznol M, Powderly JD, Smith DC, Brahmer JR, Drake CG, McDermott DF, Lawrence DP, Wolchok JD, Topalian SL, Lowy I: Safety and antitumor activity of biweekly MDX-1106 (Anti-PD-1, BMS-936558/ONO-4538) in patients with advanced refractory malignancies. Journal of Clinical Oncology 2010, 28(15).

85. Tolcher AW, Hong DS, Sullivan RJ, Mier JW, Shapiro G, Pearlberg J, Brail LH, Kharidia J, Han L, Ullmann CD et al: IPI-549-01-A phase 1/lb first in human study of IPI-549, a PI3Kγ inhibitor, as monotherapy and in combination with pembrolizumab in subjects with advanced solid tumors. Journal of Clinical Oncology 2016, 34.

86. Tolcher AW, Sznol M, Hu-Lieskovan S, Papadopoulos KP, Patnaik A, Rasco DW, Di Gravio D, Huang B, Gambhire D, Chen Y et al: Phase Ib study of PF-05082566 in combination with pembrolizumab in patients with advanced solid tumors. Journal of Clinical Oncology 2016, 34.

87. Topalian SL, Brahmer JR, Hodi FS, McDermott DF, Smith DC, Gettinger S, Taube JM, Gupta A, Wigginton JM, Sznol M: Anti-programmed death-1 (PD-1) (BMS-936558/ MDX-1106/ONO-4538) in patients (PTS) with advanced solid tumors: Clinical activity, safety, and molecular markers. Annals of Oncology 2012, 23:ix157.

88. Topalian SL, Brahmer JR, Hodi FS, McDermott DF, Smith DC, Gettinger SN, Taube JM, Drake CG, Pardoll DM, Antonia S et al: Anti-PD-1 (BMS-936558, MDX-1106) in patients with advanced solid tumors: Clinical activity, safety, and a potential biomarker for response. Journal of Clinical Oncology 2012, 30(18).

89. Topalian SL, Sznol M, Brahmer JR, McDermott DF, Smith DC, Gettinger SN, Taube JM, Drake CG, Pardoll DM, Powderly JD et al: Nivolumab (anti-PD-1; BMS-936558; ONO-4538) in patients with advanced solid tumors: Survival and long-term safety in a phase I trial. Journal of Clinical Oncology 2013, 31(15).

90. Tykodi SS, Brahmer JR, Hwu WJ, Chow LQ, Topalian SL, Hwu P, Odunsi K, Camacho LH, S Kauh J, Pitot HC et al: PD-1/PD-L1 pathway as a target for cancer immunotherapy: Safety and clinical activity of BMS-936559, an anti-PD-L1 antibody, in patients with solid tumors. Journal of Clinical Oncology 2012, 30(15).

91. Urba W, Chmielowski B, Loo D, Baughman J, Chen F, Moore P, Bonvini E, Vasselli J, Wigginton J, Rizvi N: A Phase I, open-label, dose escalation study of MGA271 in combination with ipilimumab in patients with B7-H3-expressing melanoma, squamous cell cancer of the head and neck or non-small cell lung cancer. Journal for ImmunoTherapy of Cancer 2015, 3.

92. Weiss GJ, Pierog M, Blaydorn L, Sangal A, Niu J, Farley JH, Khemka V: Phase Ib/II study of pembrolizumab plus chemotherapy: Initial results of metastatic cancer patients. Molecular Cancer Therapeutics 2015, 14(12).

93. Yamamoto N, Nokihara H, Yamada Y, Asahina H, Shibata T, Tamura Y, Seki Y, Honda K, Tanabe Y, Wakui H et al: Phase i study of anti PD-1 antibody ONO-4538 in Japanese patients with advanced solid tumors. Annals of Oncology 2012, 23:xi26.

94. Freites-Martinez A, Kwong BY, Rieger KE, Coit DG, Colevas AD, Lacouture ME: Eruptive Keratoacanthomas Associated With Pembrolizumab Therapy. JAMA dermatology 2017, 153(7):694-697.

95. Chang SS: Re: MPDL3280A (Anti-PD-L1) treatment leads to clinical activity in metastatic bladder cancer. Journal of Urology 2015, 194(4):956.

**Basic research: 9**

1. Spahn J, Peng J, Lorenzana E, Kan D, Hunsaker T, Segal E, Mautino M, Brincks E, Pirzkall A, Kelley S et al: Improved anti-tumor immunity and efficacy upon combination of the IDO1 inhibitor GDC-0919 with anti-PD-l1 blockade versus anti-PD-l1 alone in preclinical tumor models. Journal for immunotherapy of cancer 2015, 3.

2. Ingram J, Dougan M, Rashidian M, Linnebacher A, Ploegh H: Increasing antitumor efficacy of checkpoint blockade by targeting to the tumor microenvironment. European Journal of Immunology 2016, 46:35.

3. Kohrt H, Kowanetz M, Gettinger S, Powderly J, Koeppen H, Sosman JA, Cruz C, Xiao Y, Mokatrin A, Fine G et al: Intratumoral characteristics of tumor and immune cells at baseline and on-treatment correlated with clinical responses to MPDL3280A, an engineered antibody against PD-L1. Journal for immunotherapy of cancer 2013, 1.

4. Ahamadi M, Li C, Freshwater T, Van Vugt M, Mangin E, De Greef R, Stone J, Kondic A: Pooled population pharmacokinetic and immunogenicity analysis of pembrolizumab using data from KEYNOTE-001, KEYNOTE-002 and KEYNOTE-006. Journal of Pharmacokinetics and Pharmacodynamics 2015, 42(1):S34.

5. Bennati C, Leonardi G, Sidoni A, Gili A, Ricciuti B, Minotti V, Chiari R, Metro G, Ludovini V, Colabrese D et al: Programmed cell death ligand 1(PD-L1), Programmed death 1+(PD-1) lymphocytes and Tumor infiltrating lymphocytes (TILs): Are they playing a role in predicting response to anti- PD-1 therapies? Journal of Clinical Oncology 2016, 34.

6. Desbois M, Beal C, Coutzac C, Terme M, Teppaz G, Morisseau S, Bechard D, Mortier E, Chaput N: RLI, a sushi-IL-15Rα/IL-15 fusion protein, is a potent immunomodulatory agent on NK and CD8+ T cells and synergizes with anti-PD1 treatment in preclinical mouse tumor models. Cancer Research 2014, 74(19).

7. Greisen SR, Rasmussen TK, Stengaard-Pedersen K, Hetland ML, Hørslev-Petersen K, Hvid M, Deleuran B: Increased soluble programmed death-1 (sPD-1) is associated with disease activity and radiographic progression in early rheumatoid arthritis. In: Scandinavian journal of rheumatology. vol. 43; 2014: 101-108.

8. Agrawal S, Statkevich P, Bajaj G, Feng Y, Saeger S, Desai DD, Park JS, Waxman IM, Roy A, Gupta M: Evaluation of Immunogenicity of Nivolumab Monotherapy and its Clinical Relevance in Patients With Metastatic Solid Tumors. Journal of clinical pharmacology 2016.

9. Huang AC, Postow MA, Orlowski RJ, Mick R, Bengsch B, Manne S, Xu W, Harmon S, Giles JR, Wenz B et al: T-cell invigoration to tumour burden ratio associated with anti-PD-1 response. Nature 2017, 545(7652):60-65.

**Used in combination with other drugs: 31**

1. Clinical trials of PD-1 and PD-L1 inhibitors in NSCLC. In: Clinical advances in hematology & oncology. vol. 12; 2014: 14-16.

2. Antonia S, Goldberg SB, Balmanoukian A, Chaft JE, Sanborn RE, Gupta A, Narwal R, Steele K, Gu Y, Karakunnel JJ et al: Safety and antitumour activity of durvalumab plus tremelimumab in non-small cell lung cancer: a multicentre, phase 1b study. The Lancet Oncology 2016, 17(3):299-308.

3. Antonia SJ, Gettinger S, Chow LQ, Juergens R, Borghaei H, Shen Y, Harbison C, Chen AC, Ready NE, Rizvi NA: Nivolumab and ipilimumab in first-line non-small-cell lung carcinoma (NSCLC): Interim phase i results. Asia-Pacific Journal of Clinical Oncology 2014, 10:155.

4. Antonia SJ, Gettinger S, Goldman J, Chow LQ, Juergens R, Borghaei H, Brahmer JR, Shen Y, Harbison C, Chen AC et al: Safety and efficacy of first-line nivolumab (anti-PD-1; BMS-936558, ONO-4538) and ipilimumab in non-small cell lung cancer (NSCLC). International Journal of Radiation Oncology Biology Physics 2014, 90(5):S32-S33.

5. Antonia SJ, Gettinger SN, Chow LQM, Juergens RA, Borghaei H, Shen Y, Harbison C, Chen AC, Ready N, Rizvi NA: Nivolumab (anti-PD-1; BMS-936558, ONO-4538) and ipilimumab in first-line NSCLC: Interim phase I results. Journal of Clinical Oncology 2014, 32(15).

6. Antonia SJ, Rizvi NA, Chow LQ, Borghaei H, Brahmer JR, Juergens R, Shepherd FA, Laurie SA, Gerber DE, Goldman J et al: Nivolumab (anti-PD-1; BMS-936558, ONO-4538) in combination with platinum-based doublet chemotherapy (PT-DC) or erlotinib in advanced nonsmall cell lung cancer (NSCLC). Journal of Thoracic Oncology 2014, 9(9):S153.

7. Camidge R, Liu SV, Powderly J, Ready N, Hodi S, Gettinger SN, Giaccone G, Liu B, Wallin J, Funke R et al: Atezolizumab (MPDL3280A) combined with platinum-based chemotherapy in non-small cell lung cancer (NSCLC): A phase Ib safety and efficacy update. Journal of Thoracic Oncology 2015, 10(9):S176-S177.

8. Gadgeel S, Gandhi L, Borghaei H, Socinski MA, Gubens MA, Stevenson J, Sequist LV, Yang JCH, Papadimitrakopoulou V, Bourque J et al: Pembrolizumab plus chemotherapy vs chemotherapy alone as first-line therapy for NSCLC. Journal of Thoracic Oncology 2015, 10(9):S428.

9. Gadgeel SM, Stevenson J, Langer CJ, Gandhi L, Borghaei H, Patnaik A, Villaruz LC, Gubens MA, Hauke RJ, Yang JCH et al: Pembrolizumab (pembro) plus chemotherapy as front-line therapy for advanced NSCLC: KEYNOTE-021 cohorts A-C. Journal of Clinical Oncology 2016, 34.

10. Gettinger S, Chow LQ, Borghaei H, Shen Y, Harbison C, Chen AC, Rizvi NA: Safety and response with nivolumab (anti-PD-1; BMS-936558, ONO-4538) plus erlotinib in patients (PTS) with epidermal growth factor receptor mutant (EGFR MT) advanced non-small cell lung cancer (NSCLC). International Journal of Radiation Oncology Biology Physics 2014, 90(5):S34-S35.

11. Gettinger SN, Rizvi NA, Shepherd FA, Chow LQ, Laurie SA, Spigel DR, Sbar E, Shen Y, Brahmer JR: A phase I study of BMS-936558 in combination with gemcitabine/cisplatin, pemetrexed/cisplatin, or carboplatin/paclitaxel in patients with treatment-naive, stage IIIB/IV non-small-cell lung cancer. Journal of Clinical Oncology 2012, 30(15).

12. Goldberg SB, Balmanoukian A, Chaft J, Rizvi N, Sanborn RE, Rebelatto MC, Narwal R, Robbins PB, Gu Y, Karakunnel JJ et al: A Phase 1b study to evaluate the safety and antitumor activity of MEDI4736 in combination with tremelimumab in patients with advanced NSCLC. European Journal of Cancer 2015, 51:S627.

13. Hellmann MD, Rizvi NA, Goldman JW, Gettinger SN, Borghaei H, Brahmer JR, Ready NE, Gerber DE, Chow LQ, Juergens RA et al: Nivolumab plus ipilimumab as first-line treatment for advanced non-small-cell lung cancer (CheckMate 012): results of an open-label, phase 1, multicohort study. The Lancet Oncology 2017, 18(1):31-41.

14. Johnson ML, Adjei AA, Ramalingam SS, Janne PA, Dominguez G, Gabrilovich D, Deleon L, Hasapidis JL, Diede SJ, Ordentlich P et al: Preliminary results of ENCORE 601, a phase 1b/2, open-label study of entinostat (ENT) in combination with pembrolizumab (PEMBRO) in patients with non-small cell lung cancer (NSCLC). Journal of Clinical Oncology 2016, 34.

15. Liu SV, Powderly JD, Camidge DR, Ready N, Heist RS, Hodi FS, Giaccone G, Liu B, Wallin J, Funke RP et al: Safety and efficacy of MPDL3280A (anti-PD-L1) in combination with platinum-based doublet chemotherapy in patients with advanced non-small cell lung cancer (NSCLC). Journal of Clinical Oncology 2015, 33(15).

16. Morgensztern D, Harb WA, Schalper KA, Price ML, Early B, Schreiber TH: Broadening response rates to PD-1 therapy in advanced lung adenocarcinoma: Viagenpumatucel-l (HS-110) in combination with nivolumab in the ongoing DURGA trial. Journal of Clinical Oncology 2016, 34.

17. Papadimitrakopoulou V, Cappuzzo F, Jotte RM, Reck M, Mok T, Sandler A, Waterkamp D, Coleman S, Sugitani Y, Socinski MA: Phase III clinical trials of atezolizumab combined with chemotherapy in chemotherapy-naive patients with advanced NSCLC. Journal of Clinical Oncology 2016, 34.

18. Papadimitrakopoulou V, Patnaik A, Borghaei H, Stevenson J, Gandhi L, Gubens MA, Yang JCH, Sequist LV, Ge JY, Bourque J et al: Pembrolizumab (pembro; MK-3475) plus platinum doublet chemotherapy (PDC) as frontline therapy for advanced non-small cell lung cancer (NSCLC): KEYNOTE-021 Cohorts A and C. Journal of Clinical Oncology 2015, 33(15).

19. Patnaik A, Socinski MA, Gubens MA, Gandhi L, Stevenson J, Bachman RD, Bourque J, Ge JY, Im E, Gadgeel SM: Phase 1 study of pembrolizumab (pembro; MK-3475) plus ipilimumab (IPI) as second-line therapy for advanced non-small cell lung cancer (NSCLC): KEYNOTE-021 cohort D. Journal of Clinical Oncology 2015, 33(15).

20. Pinder MC, Rizvi NA, Goldberg SB, Balmanoukian AS, Narwal R, Robbins PB, D'Angelo G, Blake-Haskins A, Karakunnel JJ, Antonia SJ: A phase 1b open-label study to evaluate the safety and tolerability of MEDI4736, an anti-PD-L1 antibody, in combination with tremelimumab in subjects with advanced non-small cell lung cancer. Journal of Clinical Oncology 2014, 32(15).

21. Rizvi N, Balmanoukian A, Goldberg SB, Chaft J, Sanborn RE, Rebelatto MC, Narwal R, Robbins PB, Gu Y, Karakunnel JJ et al: Phase 1b study of the safety and antitumour activity of durvalumab (MEDI4736) + tremelimumab in advanced NSCLC. Annals of Oncology 2015, 26:ix126.

22. Rizvi N, Chaft J, Balmanoukian A, Goldberg SB, Sanborn RE, Steele KE, Rebelatto MC, Gu Y, Karakunnel JJ, Antonia S: Tumor response from durvalumab (MEDI4736) + tremelimumab treatment in patients with advanced non-small cell lung cancer (NSCLC) is observed regardless of PD-L1 status. Journal for ImmunoTherapy of Cancer 2015, 3.

23. Rizvi NA, Antonia SJ, Chow LQM, Brahmer JR, Juergens RA, Borghaei H, Shepherd FA, Laurie SA, Gerber DE, Goldman JW et al: A phase I study of nivolumab (anti-PD-1; BMS-936558, ONO-4538) plus platinum-based doublet chemotherapy (PT-doublet) in chemotherapy-naive non-small cell lung cancer (NSCLC) patients (pts). Journal of Clinical Oncology 2013, 31(15).

24. Rizvi NA, Chow LQM, Borghaei H, Shen Y, Harbison C, Alaparthy S, Chen AC, Gettinger SN: Safety and response with nivolumab (anti-PD-1; BMS-936558, ONO-4538) plus erlotinib in patients (pts) with epidermal growth factor receptor mutant (EGFR MT) advanced NSCLC. Journal of Clinical Oncology 2014, 32(15).

25. Rizvi NA, Hellmann MD, Brahmer JR, Juergens RA, Borghaei H, Gettinger S, Chow LQ, Gerber DE, Laurie SA, Goldman JW et al: Nivolumab in Combination With Platinum-Based Doublet Chemotherapy for First-Line Treatment of Advanced Non-Small-Cell Lung Cancer. Journal of clinical oncology : official journal of the American Society of Clinical Oncology 2016, 34(25):2969-2979.

26. Shaw A, Loong H, Tan DSW, Griscti K, Gao H, Finckenstein F, Scott J, Vansteenkiste J: A multicenter phase 1b study of ceritinib plus nivolumab in patients with ALK+ NSCLC. Journal of Thoracic Oncology 2015, 10(9):S430.

27. Tatipalli M, Song X, Pak M, Chavez C, Liang M, Lu H, Schwickart M, Karakunnel JJ, Robbins PB, Jin X et al: Pharmacokinetics and pharmacodynamics of MEDI4736, a fully human anti-programmed death ligand 1 (PD-L1) monoclonal antibody, in combination with tremelimumab in patients with advanced non-small cell lung cancer (NSCLC). Journal of Clinical Oncology 2015, 33(15).

28. Waterhouse D, Derosa W, Duval Fraser C, Gutierrez M, Ko A, Jin Ong T, Pierce D, Stergiopoulos S, Kelly K: First-line nivolumab + nab-paclitaxel + carboplatin (C) in advanced NSCLC. Journal of Thoracic Oncology 2015, 10(9):S427-S428.

29. Weiss GJ, Barndt H, Blaydorn L, Sangal A, Khemka V: Phase IB/II study of pembrolizumab plus chemotherapy in advanced cancer: Results of lung cancer patients receiving ≥ 1 prior line of therapy. Journal of Thoracic Oncology 2015, 10(9):S742-S743.

30. Weiss GJ, Waypa J, Blaydorn L, Coats J, McGahey K, Sangal A, Niu J, Lynch CA, Farley JH, Khemka V: A phase Ib study of pembrolizumab plus chemotherapy in patients with advanced cancer (PembroPlus). British journal of cancer 2017, 117(1):33-40.

31. Yeh S, Bazhenova L, Lee G, Huang L: A phase 1 trial combining plinabulin and nivolumab for metastatic squamous NSCLC. Journal of Thoracic Oncology 2015, 10(9):S551.

Other (off topic, education, medico economic studies) : 214

**Studies excluded reading articles (n= 119)**

**CASE REPORT**

**No irAEs data: 7**

1. Bearz A, Perin T, Cancian L, Berto E, Sartor I, Tirelli U: Immune checkpoint inhibitors and response analysis: a tough challenge. A case report. BMC research notes 2016, 9:349.

2. Higuchi M, Owada Y, Inoue T, Watanabe Y, Yamaura T, Fukuhara M, Hasegawa T, Suzuki H: FDG-PET in the evaluation of response to nivolumab in recurrent non-small-cell lung cancer. World journal of surgical oncology 2016, 14(1):238.

3. Kanazu M, Uenami T, Yano Y, Nakatsubo S, Hosono Y, Ishijima M, Akazawa Y, Yamaguchi T, Urasaki K, Mori M et al: Case series of pleomorphic carcinomas of the lung treated with nivolumab. Thoracic cancer 2017, 8(6):724-728.

4. Nozawa Y, Oka Y, Oosugi J, Takemura S: Immunotherapy for pulmonary squamous cell carcinoma and colon carcinoma with pembrolizumab: A case report. Medicine 2018, 97(19):e0718.

5. Ramos-Levi AM, Rogado J, Sanchez-Torres JM, Colomer R, Marazuela M: Nivolumab-induced thyroid dysfunction in patients with lung cancer. Endocrinologia, diabetes y nutricion 2018.

6. Zapata E, Mennecier B, Leduc C, Chatron E, Quoix E: [Prolonged response with paclitaxel after immunotherapy by pembrolizumab in lung cancer]. Revue de pneumologie clinique 2016, 72(5):300-304.

7. Davar D, Socinski MA, Dacic S, Burns TF: Near complete response after single dose of nivolumab in patient with advanced heavily pre-treated KRAS mutant pulmonary adenocarcinoma. Experimental Hematology and Oncology 2015, 4(1).

**Auto immunity not proved: 1**

1. Abe J, Sato T, Tanaka R, Okazaki T, Takahashi S: Nivolumab-Induced Severe Akathisia in an Advanced Lung Cancer Patient. The American journal of case reports 2016, 17:880-882.

**Used in combination with other drugs: 1**

1. John S, Antonia SJ, Rose TA, Seifert RP, Centeno BA, Wagner AS, Creelan BC: Progressive hypoventilation due to mixed CD8(+) and CD4(+) lymphocytic polymyositis following tremelimumab - durvalumab treatment. Journal for immunotherapy of cancer 2017, 5(1):54.

**CLINICAL TRIALS**

**Experiment plan: 27**

1. Verschraegen C, Goel S, Chen F, R. Spigel D, Iannotti N, Bajars M, Von Heydebreck A, Kelly K: Avelumab (MSB0010718C), an anti-PD-L1 antibody, evaluated in a phase IB trial as a first-line treatment for patients with metastatic NSCLC. Journal of Thoracic Oncology 2015, 10(9):S666.

2. Park K, Vansteenkiste J, Bajars M, Helwig C, Barlesi F: Avelumab (MSB0010718C), an anti-Pd-L1 antibody, evaluated in a phase iii trial versus docetaxel in patients with relapsing NSCLC. Journal of Thoracic Oncology 2015, 10(9):S664.

3. Reck M, DeGreen HP, Rose AL, Pavlakis N, Derjcke SM, Radic J, Jeppesen N, Thomas M, Losonczy G, Kalinka-Warzocha E et al: Avelumab (MSB0010718C; anti-PD-LI) vs platinum-based doublet as first-line treatment for metastatic or recurrent PDL1-positive non-small-cell lung cancer: The phase 3 JAVELIN Lung 100 trial. Journal of Clinical Oncology 2016, 34.

4. Felip E, Van Meerbeeck J, Wolf J, Ardizzoni A, Li A, Srinivasan S, Popat S: CheckMate 171: A multicenter phase 2 trial of nivolumab (nivo) in patients (pts) with stage IIIB/IV squamous cell (SQ) NSCLC who have received ≥1 prior systemic treatment. Journal of Thoracic Oncology 2016, 11(4):S141.

5. Rizvi NA, Chow LQM, Dirix LY, Gettinger SN, Gordon MS, Kabbinavar FF, Von Pawel J, Soria JC, Chappey C, Mokatrin A et al: Clinical trials of MPDL3280A (anti-PDL1) in patients (pts) with non-small cell lung cancer (NSCLC). Journal of Clinical Oncology 2014, 32(15).

6. O'Brien MER, Hasan B, Dafni U, Menis J, Peters S, De Waele M, Stahel RA, Van Schil P, Coukos G, Lantuejoul S et al: EORTC-ETOP randomized, phase 3 trial with anti-PD-1 monoclonal antibody pembrolizumab versus placebo for patients with early stage non-small cell lung cancer (NSCLC) after resection and standard adjuvant chemotherapy: PEARLS (NCT02504372). Journal of Clinical Oncology 2016, 34.

7. Brahmer JR, Kim ES, Zhang J, Smith MM, Rangwala RA, O'Brien MER: KEYNOTE-024: Phase III trial of pembrolizumab (MK-3475) vs platinum-based chemotherapy as first-line therapy for patients with metastatic non-small cell lung cancer (NSCLC) that expresses programmed cell death ligand 1 (PD-L1). Journal of Clinical Oncology 2015, 33(15).

8. Lopes G, Watson PA, Zhang J, Rangwala R, Mok T: Keynote-042: Phase 3 study of pembrolizumab (MK-3475) versus platinum doublet chemotherapy as first-line therapy for PD-l1-positive nonsmall cell lung cancer. In: Journal of thoracic oncology. vol. 9; 2014: S192.

9. Hellmann M, Ramalingam S, Reck M, O'Byrne K, Paz-Ares L, Harbison CT, Bhagavatheeswaran P, Nathan F, Brahmer J: An open label randomized Phase III trial of nivolumab or nivolumab plus ipilimumab vs platinum doublet chemotherapy (PT-DC) in patients with chemotherapy-naïve stage IV or recurrent non-small cell lung cancer (NSCLC) (CheckMate 227). Journal for ImmunoTherapy of Cancer 2015, 3.

10. Mok T, Wu YL, Sadowski S, Zhang J, Rangwala R, De Lima Lopes G: Pembrolizumab (MK-3475) versus platinum-based chemotherapy for PD-L1+ non-small cell lung cancer (NSCLC): Randomized, open-label, phase 3 KEYNOTE-042 study. Annals of Oncology 2015, 26:ix147.

11. Mok T, Wu YL, Sadowski S, Zhang J, Rangwala R, Kush D, De Lima Lopes G: Pembrolizumab (MK-3475) versus platinum-based chemotherapy for PD-L1+ NSCLC in a phase 3, randomized, open-label study: KEYNOTE-042. Journal of Thoracic Oncology 2016, 11(4):S142.

12. Brahmer JR, Gottfried M, Li X, Smith M, Rangwala RA, O'Brien ME: Pembrolizumab vs platinum-based chemotherapy for PD-l1-strong-positive NSCLC. Journal of Thoracic Oncology 2015, 10(9):S428.

13. Garassino MC, Barlesi F, Chaft J, Shi K, Ibrahim R, Stockman P, Ballas M, Rizvi NA: Phase 2 study of medi4736 in patients with PD-l1+ locally advanced or metastatic stage IIIb-IV NSCLC treated with ≥ 2 prior regimens (atlantic). Journal of Thoracic Oncology 2015, 10(9):S429.

14. Rizvi N, Ballas M, Jayawardene D, Stockman PK, Powderly JD: A phase 2, non-comparative, open-label, international study of MEDI4736 in patients with locally advanced or metastatic PD-l1-positive NSCLC (stage IIIb-IV) who have received ≥ 2 prior systemic treatment regimens (ATLANTIC). Annals of Oncology 2014, 25:vi6.

15. Gray JE, Garassino MC, Vansteenkiste JF, Ballas M, Jayawardene D, Stockman PK, Powderly JD, Rizvi N: Phase 2, open-label, international, non-comparative study of MEDI4736 in patients with locally advanced or metastatic, PD-l1 + , stage 3B-4 NSCLC who have received ≥2 prior systemic treatment regimens (ATLANTIC). Annals of Oncology 2015, 26:i43.

16. Mok T, Wu YL, Watson PA, Zhang J, Rangwala RA, Lopes G: Phase 3 KEYNOTE-042 trial of pembrolizumab (MK-3475) versus platinum doublet chemotherapy in treatment-naive patients (pts) with PD-L1-positive advanced non-small cell lung cancer (NSCLC). In: Journal of clinical oncology. vol. 33; 2015.

17. Mok T, Wu YL, Sadowski S, Zhang J, Rangwala R, De Lima Lopes G: Phase 3 study of pembrolizumab vs platinum-based chemotherapy for PD-l1+ NSCLC. Journal of Thoracic Oncology 2015, 10(9):S554.

18. Hall RD, Gadgeel SM, Garon EB, Bria E, Reck M, Vida J, Zhou H, Raftopoulos H, Gandhi L: Phase 3 study of platinum-based chemotherapy with or without pembrolizumab for first-line metastatic, nonsquamous non-small cell lung carcinoma (NSCLC): KEYNOTE-189. Journal of Clinical Oncology 2016, 34.

19. Cho BC, Kim JH, Villegas A, Frusch N, Murakami S, Shi K, Ibrahim R, Ballas M, Antonia SJ: Phase 3, double-blind, placebo-controlled study of medi4736 after chemoradiation in stage iii, locally advanced, unresectable NSCLC (pacific). Journal of Thoracic Oncology 2015, 10(9):S668.

20. Levy BP, Giaccone G, Besse B, Begic D, Wu X, Fandi A, Paz-Ares L: A phase II multicenter, randomized, placebo-controlled, double-blind study of CC-486 plus pembrolizumab (pembro) vs pembro plus placebo (PBO) in previously treated patients (pts) with locally advanced/metastatic non-small cell lung cancer (NSCLC). Journal of Clinical Oncology 2016, 34.

21. Herbst RS, De Marinis F, Jassem J, Spigel DR, Shankar G, Mocci S, Sandler A, Lopez-Chavez A, Li S, Giaccone G: Phase III clinical trials of atezolizumab compared with standard chemotherapy in PD-L1-selected chemotherapy-naïve patients with advanced NSCLC. Annals of Oncology 2015, 26:ix105-ix106.

22. Mok TSK, Cappuzzo F, Jotte RM, Reck M, Papadimitrakopoulou V, Sandler A, Waterkamp D, Verret W, Shen Y, Socinski MA: Phase III clinical trials of atezolizumab in combination with chemotherapy in chemotherapy-naive patients with advanced NSCLC. Annals of Oncology 2015, 26:ix106.

23. Planchard D, Yokoi T, McCleod MJ, Fischer JR, Kim YC, Ballas M, Shi K, Soria JC: A Phase III Study of Durvalumab (MEDI4736) With or Without Tremelimumab for Previously Treated Patients With Advanced NSCLC: Rationale and Protocol Design of the ARCTIC Study. Clinical lung cancer 2016, 17(3):232-236.e231.

24. Planchard D, Shtivelband M, Levy BP, Hussein M, Shi K, Ibrahim R, Ballas M, Soria JC: A phase III study of medi4736 (M) an anti-pd-l1 antibody ± tremelimumab (T), vs standard of care (SOC), in patients with advanced NSCLC (arctic). Journal of Thoracic Oncology 2015, 10(9):S429.

25. Planchard D, Shtivelband M, Shi K, Ibrahim R, Ballas M, Soria JC: A phase III study of MEDI4736 (M), an anti-PD-L1 antibody, in monotherapy or in combination with Tremelimumab (T), versus standard of care (SOC) in patients (pts) with advanced non-small cell lung cancer (NSCLC) who have received at least two prior systemic treatment regimens (ARCTIC). Journal of Clinical Oncology 2015, 33(15).

26. Carbone DP, Socinski MA, Chen AC, Bhagavatheeswaran P, Reck M, Paz-Ares L: A phase III, randomized, open-label trial of nivolumab (anti-PD-1; BMS-936558, ONO-4538) versus investigator's choice chemotherapy (ICC) as first-line therapy for stage IV or recurrent PD-L1+ non-small cell lung cancer (NSCLC). In: Journal of clinical oncology. vol. 32; 2014.

27. Gerber DE, Urbanic JJ, Langer C, Hu C, Chang IF, Lu B, Movsas B, Jeraj R, Curran WJ, Bradley JD: Treatment Design and Rationale for a Randomized Trial of Cisplatin and Etoposide Plus Thoracic Radiotherapy Followed by Nivolumab or Placebo for Locally Advanced Non-Small-Cell Lung Cancer (RTOG 3505). Clinical lung cancer 2017, 18(3):333-339.

**No irAEs data: 19**

1. Sadjadian P, Jäger D, Keilholz U, Von Pawel J, Grimm MO, Bögemann M: Updated safety and clinical activity of durvalumab monotherapy in previously treated patients with stage IIIB/IV NSCLC. Oncology Research and Treatment 2018, 41:107.

2. Balmanoukian AS, Rizvi NA, Garon EB, Patnaik A, Gandhi L, Leighl NB, Goldman JW, Eder JP, Johnson EA, Blumenschein GR et al: Safety and clinical activity of MK-3475 as initial therapy in patients with advanced non-small cell lung cancer (NSCLC). In: International Journal of Radiation Oncology Biology Physics. vol. 90; 2014: S1-s2.

3. Brahmer JR, Horn L, Antonia S, Spigel DR, Gandhi L, Sequist LV, Wigginton J, McDonald D, Kollia G, Gupta AK et al: Clinical activity and safety of anti-PD1 (BMS-936558, MDX-1106) in patients with advanced non-small-cell lung cancer (NSCLC). Journal of Clinical Oncology 2012, 30(15).

4. Crino L, Bidoli P, Delmonte A, Grossi F, De Marinis F, Sperandi F, Vitiello F, Vitali M, Soto Parra HJ, Scagnoli S et al: Italian cohort of nivolumab Expanded Access Programme (EAP): Preliminary data from a real-world population. Journal of Clinical Oncology 2016, 34.

5. Garon EB, Balmanoukian A, Hamid O, Hui R, Gandhi L, Leighl N, Gubens M, Goldman JW, Lubiniecki GM, Emancipator K et al: MK-3475 monotherapy for previously treated non-small cell lung cancer (NSCLC): Preliminary safety and clinical activity. Clinical Cancer Research 2014, 20(2).

6. Goldberg SB, Gettinger SN, Mahajan A, Chiang AC, Herbst RS, Sznol M, Tsiouris AJ, Cohen J, Vortmeyer A, Jilaveanu L et al: Pembrolizumab for patients with melanoma or non-small-cell lung cancer and untreated brain metastases: early analysis of a non-randomised, open-label, phase 2 trial. The Lancet Oncology 2016, 17(7):976-983.

7. Horn L, Spigel DR, Gettinger SN, Antonia SJ, Gordon MS, Herbst RS, Sequist LV, Chappey C, Kowanetz M, Sandler A et al: Clinical activity, safety and predictive biomarkers of the engineered antibody MPDL3280A (anti-PDL1) in non-small cell lung cancer (NSCLC): Update from a phase Ia study. Journal of Clinical Oncology 2015, 33(15).

8. Nishio M, Hida T, Nakagawa K, Sakai H, Nogami N, Atagi S, Takahashi T, Nokihara H, Saka H, Takenoyama M et al: Phase II studies of nivolumab (anti-PD-1, BMS-936558, ONO-4538) in patients with advanced squamous (sq) or nonsquamous (non-sq) non-small cell lung cancer (NSCLC). Journal of Clinical Oncology 2015, 33(15).

9. Rizvi NA, Antonia SJ, Shepherd FA, Chow LQ, Goldman J, Shen Y, Chen AC, Gettinger S: Nivolumab (anti-PD-1; BMS-936558, ONO-4538) maintenance as monotherapy or in combination with bevacizumab (BEV) for non-small cell lung cancer (NSCLC) previously treated with chemotherapy. In: International Journal of Radiation Oncology Biology Physics. vol. 90; 2014: S32.

10. Spigel DR, Chaft JE, Gettinger SN, Chao BH, Dirix LY, Schmid P, Man Chow LQ, Chappey C, Kowanetz M, Sandler A et al: Clinical activity and safety from a phase II study (FIR) of MPDL3280A (antiPDL1) in PD-L1-selected patients with non-small cell lung cancer (NSCLC). Journal of Clinical Oncology 2015, 33(15).

11. Besse B, Johnson M, Jänne PA, Garassino M, Eberhardt WEE, Peters S, Toh CK, Kurata T, Li Z, Kowanetz M et al: Phase II, single-arm trial (BIRCH) of atezolizumab as first-line or subsequent therapy for locally advanced or metastatic PD-L1-selected non-small cell lung cancer (NSCLC). European Journal of Cancer 2015, 51:S717-S718.

12. Brustugun OT, Sprauten M, Helland A: C-reactive protein (CRP) as a predictive marker for immunotherapy in lung cancer. Journal of Clinical Oncology 2016, 34.

13. Corny J, Maritaz C, Renet S, Jouveshomme S, Fallet V, Gazaniol C, Monnet CM, Tredaniel J, Jardin M: Real-life efficacy and safety of nivolumab for non-small cell lung cancer: A 10-months French cohort. Journal of Clinical Oncology 2016, 34.

14. Dudnik E, Moskovitz M, Wollner M, Zer A, Bar J, Agbarya A, Idan T, Shechtman Y, Abu Amna M, Peled N: Anti-PD-1 antibodies in non-small cell lung cancer (NSCLC): The real-life setting experience. Journal of Thoracic Oncology 2016, 11(4):S136.

15. Forde PM, Smith K, Chaft JE, Hellmann MD, Merghoub T, Wolchok JD, Yang SC, Battafarano RJ, Gabrielson E, Georgiades C et al: Neoadjuvant anti-PDI, nivolumab, in early stage resectable non-small-cell lung cancer. Journal of Clinical Oncology 2016, 34.

16. Horn L, Herbst RS, Spigel D, Gettinger SN, Gordon MS, Hollebecque A, Gandhi L, Felip E, Heist R, Mokatrin A et al: An analysis of the relationship of clinical activity to baseline EGFR status, PDL1 expression and prior treatment history in patients with non-small cell lung cancer (NSCLC) following PD-l1 blockade with MPDL3280A (Anti-PDL1). Journal of Thoracic Oncology 2013, 8:S364.

17. Hussein M, McCleod M, Chandler J, Blumenschein Jr G, Schwartzberg L, Burris H, Waterhouse D, Jotte R, Bauer T, Thompson D et al: Safety and efficacy of nivolumab in an ongoing trial of a PD-l1+/-patient population with metastatic nonsmall cell lung cancer. Asia-Pacific Journal of Clinical Oncology 2015, 11:124-125.

18. Spigel DR, Gettinger SN, Horn L, Herbst RS, Gandhi L, Gordon MS, Cruz C, Conkling P, Cassier PA, Antonia SJ et al: Clinical activity, safety, and biomarkers of MPDL3280A, an engineered PD-L1 antibody in patients with locally advanced or metastatic non-small cell lung cancer (NSCLC). Journal of Clinical Oncology 2013, 31(15).

19. Yam AO, Mersiadis A, Gao B, Hui R, Nagrial A: Incidence and grade of pneumonitis in advanced non-small cell lung cancer (NSCLC) patients treated with anti-PD1 antibodies. Asia-Pacific Journal of Clinical Oncology 2016, 12:71-72.

**Used in combination with other drugs: 6**

1. Durm GA, Kio EA, Fisher WB, Titzer ML, Jabbour S, Breen TIM, Liu Z, Hanna NH: Phase II trial of consolidation Pembrolizumab following concurrent chemoradiation in patients (pts) with unresectable or inoperable stage III non-small cell lung cancer (NSCLC): Initial safety data from HCRN LUN 14-179. Journal of Clinical Oncology 2016, 34.

2. Giaccone G, Camidge DR, Liu SV, Powderly J, Hodi FS, Gettinger SN, Heist RS, Liu B, Wallin J, Funke R et al: Safety, activity and biomarkers of atezolizumab (MPDL3280A) with platinum-based chemotherapy (chemo) in non-small cell lung cancer (NSCLC): A Phase Ib study. European Journal of Cancer 2015, 51:S107-S108.

3. Hellmann MD, Gettinger SN, Goldman JW, Brahmer JR, Borghaei H, Chow LQ, Ready N, Gerber DE, Juergens RA, Shepherd FA et al: CheckMate 012: Safety and efficacy of first-line (1L) nivolumab (nivo; N) and ipilimumab (ipi; I) in advanced (adv) NSCLC. Journal of Clinical Oncology 2016, 34.

4. Hellmann MD, Rizvi N, Gettinger SN, Goldman J, Chow LQ, Juergens R, Borghaei H, Brahmer J, Shen Y, Harbison CT et al: Safety and efficacy of first-line nivolumab (NIVO) and ipilimumab (IPI) in non-small cell lung cancer (NSCLC). European Journal of Cancer 2015, 51:S632-S633.

5. Kanda S, Tanaka A, Utsumi H, Sunami K, Mizugaki H, Horinouchi H, Fujiwara Y, Nokihara H, Yamamoto N, Tamura T: Phase i study of anti-PD-1 antibody ONO-4538 and chemotherapy in patients with advanced non-small-cell lung cancer. Annals of Oncology 2014, 25:v56.

6. Rizvi NA, Gettinger SN, Goldman JW, Hellmann MD, Chow LQ, Juergens R, Borghaei H, Brahmer JR, Shen Y, Harbison C et al: Safety and efficacy of first-line nivolumab (nivo; anti-programmed death-1 [PD-1]) and ipilimumab in non-small cell lung cancer (NSCLC). Journal of Thoracic Oncology 2015, 10(9):S176.

**Duplicate population: 58**

1. Goldberg SB, Gettinger SN, Mahajan A, Herbst RS, Chiang AC, Tsiouris AJ, Vortmeyer A, Jilaveanu L, Speaker S, Madura M et al: Activity and safety of pembrolizumab in patients with metastatic non-small cell lung cancer with untreated brain metastases. Journal of Clinical Oncology 2015, 33(15).

2. Vansteenkiste J, Fehrenbacher L, Spira AI, Mazieres J, Park K, Smith D, Artal-Cortes A, Lewanski C, Braiteh F, Yi J et al: Atezolizumab monotherapy vs docetaxel in 2L/3L non-small cell lung cancer: Primary analyses for efficacy, safety and predictive biomarkers from a randomized phase II study (POPLAR). In: European Journal of Cancer ( varpagings). vol. 51; 2015: S716-s717.

3. Gogia A, Kakar A: Atezolizumab versus docetaxel for patients with previously treated non-small-cell lung cancer. Current Medicine Research and Practice 2016, 6(2):96-97.

4. Gulley JL, Spigel D, Kelly K, Chandler JC, Rajan A, Hassan R, Lee Wong DJ, Leach J, Edenfield WJ, Wang D et al: Avelumab (MSB0010718C), an anti-PD-L1 antibody, in advanced NSCLC patients: A phase 1b, open-label expansion trial in patients progressing after platinum-based chemotherapy. Journal of Clinical Oncology 2015, 33(15).

5. Gulley JL, Rajan A, Spigel DR, Iannotti N, Chandler J, Wong DJL, Leach JL, Edenfield WJ, Wang D, Bajars M et al: Avelumab (MSB0010718C), an anti-PD-L1 antibody, in patients with metastatic or recurrent non-small-cell lung cancer progressing after platinum-based chemotherapy: A phase Ib trial. European Journal of Cancer 2015, 51:S629.

6. Kazandjian D, Khozin S, Blumenthal G, Zhang L, Tang S, Libeg M, Kluetz P, Sridhara R, Keegan P, Pazdur R: Benefit-Risk Summary of Nivolumab for Patients With Metastatic Squamous Cell Lung Cancer After Platinum-Based Chemotherapy: A Report From the US Food and Drug Administration. JAMA oncology 2016, 2(1):118-122.

7. Brahmer JR, Rizvi NA, Lutzky J, Khleif S, Blake-Haskins A, Li X, Robbins PB, Vasselli J, Ibrahim RA, Antonia SJ: Clinical activity and biomarkers of MEDI4736, an anti-PD-L1 antibody, in patients with NSCLC. Journal of Clinical Oncology 2014, 32(15).

8. Gettinger S, Horn L, Antonia SJ, Spigel D, Gandhi L, Sequist LV, Wigginton JM, Kollia G, Gupta A, Brahmer JR: Clinical activity and safety of anti-programmed death-1 (PD-1) (BMS-936558/MDX-1106/ONO-4538) in patients (PTS) with advanced non-small cell lung cancer (NSCLC). Annals of Oncology 2012, 23:ix405-ix406.

9. Soria JC, Cruz C, Bahleda R, Delord JP, Horn L, Herbst RS, Spigel D, Mokatrin A, Fine G, Gettinger S: Clinical activity, safety and biomarkers of PD-L1 blockade in non-small cell lung cancer (NSCLC): Additional analyses from a clinical study of the engineered antibody MPDL3280A (anti-PDL1). European Journal of Cancer 2013, 49:S798.

10. Brahmer JR, Horn L, Antonia SJ, Spigel DR, Sequist LV, Ahlers CM, Sankar V, Kollia G, Gettinger S: Clinical activity, safety and subpopulation response analysis of nivolumab (anti-PD-1; BMS-936558; ONO-4538) in patients (PT) with non-small cell lung cancer (NSCLC). Journal of Thoracic Oncology 2014, 9(4):S39.

11. Soria JC, Fløtten Ø, Horn L, Felip E, Gandhi L, Hui R, Hellmann M, Leighl N, Zhang J, Kondic A et al: Efficacy and Safety of Pembrolizumab (Pembro; MK-3475) for Patients (Pts) With Previously Treated Advanced Non-Small Cell Lung Cancer (NSCLC) Enrolled in KEYNOTE-001. European Journal of Cancer 2015, 51:S726-S727.

12. Hellmann MD, Garon EB, Gandhi L, Hui R, Zhang J, Rangwala R, Lubiniecki G, Rizvi NA: Efficacy of pembrolizumab in key subgroups of patients with advanced NSCLC. Journal of Thoracic Oncology 2015, 10(9):S270.

13. Spira AI, Park K, Mazières J, Vansteenkiste JF, Rittmeyer A, Ballinger M, Waterkamp D, Kowanetz M, Mokatrin A, Fehrenbacher L: Efficacy, safety and predictive biomarker results from a randomized phase II study comparing MPDL3280A vs docetaxel in 2L/3L NSCLC (POPLAR). Journal of Clinical Oncology 2015, 33(15).

14. Venkatachalam M, Stenehjem DD, Pietri G, Penrod JR, Korytowsky B: Estimated costs of managing treatment-related adverse events (TRAEs) of nivolumab (nivo) and docetaxel (doc) in the CheckMate 017 and CheckMate 057 phase III non-small cell lung cancer (NSCLC) trials. Journal of Clinical Oncology 2016, 34.

15. Gralla RJ, Coon C, Taylor F, Penrod JR, DeRosa M, Dastani H, Orsini L, Reck M: Evaluation of disease-related symptoms in patients (pts) with advanced squamous (SQ) non-small cell lung cancer (NSCLC) treated with nivolumab (NIVO) or docetaxel (DOC). In: Oncology Research and Treatment ( varpagings). vol. 38; 2015: 14-16.

16. Gralla RJ, Coon C, Taylor F, Penrod JR, Derosa M, Dastani H, Orsini L, Reck M: Evaluation of disease-related symptoms in patients with advanced squamous non-small cell lung cancer treated with nivolumab or docetaxel. Journal of Thoracic Oncology 2015, 10(9):S233-S234.

17. Reck M, Coon C, Taylor F, DeRosa M, Penrod JR, Dastani H, Orsini L, Gralla RJ: Evaluation of overall health status in patients with advanced squamous non-small cell lung cancer treated with nivolumab or docetaxel in CheckMate 017. European Journal of Cancer 2015, 51:S599-S600.

18. Kazandjian D, Suzman DL, Blumenthal G, Mushti S, He K, Libeg M, Keegan P, Pazdur R: FDA approval summary: Nivolumab for the treatment of metastatic non-small cell lung cancer with progression on or after platinum-based chemotherapy. In: Oncologist. vol. 21; 2016: 634-642.

19. Sul J, Blumenthal GM, Jiang X, He K, Keegan P, Pazdur R: FDA approval summary: Pembrolizumab for the treatment of patients with metastatic non-small cell lung cancer whose tumors express programmed death-ligand 1. Oncologist 2016, 21(5):643-650.

20. Rizvi NA, Shepherd FA, Antonia SJ, Brahmer JR, Chow LQ, Goldman J, Juergens R, Borghaei H, Ready NE, Gerber DE et al: First-line monotherapy with nivolumab (anti-PD-1; BMS-936558, ONO-4538) in advanced non-small cell lung cancer (NSCLC): Safety, efficacy, and correlation of outcomes with PD-l1 status. International Journal of Radiation Oncology Biology Physics 2014, 90(5):S31.

21. Gettinger SN, Hellmann MD, Shepherd FA, Antonia SJ, Brahmer J, Chow LQ, Goldman J, Juergens R, Borghaei H, Ready NE et al: First-line monotherapy with nivolumab (NIVO) in advanced non-small cell lung cancer (NSCLC): Safety, efficacy, and biomarker analyses. European Journal of Cancer 2015, 51:S632.

22. Gettinger SN, Hellmann MD, Shepherd FA, Antonia SJ, Brahmer JR, Chow LQM, Goldman JW, Juergens RA, Borghaei H, Ready N et al: First-line monotherapy with nivolumab (NIVO; anti-programmed death-1 [PD-1]) in advanced non-small cell lung cancer (NSCLC): Safety, efficacy and correlation of outcomes with PD-1 ligand (PD-L1) expression. Journal of Clinical Oncology 2015, 33(15).

23. Gettinger SN, Shepherd FA, Antonia SJ, Brahmer JR, Chow LQM, Juergens RA, Borghaei H, Shen Y, Harbison C, Alaparthy S et al: First-line nivolumab (anti-PD-1; BMS-936558, ONO-4538) monotherapy in advanced NSCLC: Safety, efficacy, and correlation of outcomes with PD-L1 status. Journal of Clinical Oncology 2014, 32(15).

24. Brahmer JR, Rodriguez-Abreu D, Robinson AG, Hui R, Csoszi T, Fulop A, Gottfried M, Peled N, Tafreshi A, Cuffe S et al: Health-related quality-of-life results for pembrolizumab versus chemotherapy in advanced, PD-L1-positive NSCLC (KEYNOTE-024): a multicentre, international, randomised, open-label phase 3 trial. The Lancet Oncology 2017, 18(12):1600-1609.

25. Brahmer JR, Rodriguez-Abreu D, Robinson AG, Hui R, Csoszi T, Fulop A, Gottfried M, Peled N, Tafreshi A, Cuffe S et al: Health-related quality-of-life results for pembrolizumab versus chemotherapy in advanced, PD-L1-positive NSCLC (KEYNOTE-024): a multicentre, international, randomised, open-label phase 3 trial. The Lancet Oncology 2017, 18(12):1600-1609.

26. Herbst RS, Kim DW, Felip E, Perez-Gracia JL, Garon EB, Han JY, Molina J, Kim JH, Gervais R, Ahn MJ et al: Lat-breaking abstracts KEYNOTE-010: Phase 2/3 study of pembrolizumab (MK-3475) vs docetaxel for PD-L1-positive NSCLC after platinum-based therapy. Annals of Oncology 2015, 26:ix162.

27. Horn L, Rizvi NA, Mazières J, Planchard D, Stinchcombe TE, Dy GK, Antonia SJ, Léna H, Minenza E, Mennecier B et al: Longer-term follow-up of a phase 2 study (checkmate 063) of nivolumab in patients with advanced, refractory squamous non-small cell lung cancer. Journal of Thoracic Oncology 2015, 10(9):S175-S176.

28. Gettinger SN, Horn L, Gandhi L, Spigel DR, Antonia SJ, Rizvi NA, Powderly JD, Heist RS, Carvajal RD, Jackman DM et al: Long-term survival, clinical activity, and safety of nivolumab (anti-PD-1; BMS-936558, ONO-4538) in patients (PTS) with advanced non-small cell lung cancer (NSCLC). International Journal of Radiation Oncology Biology Physics 2014, 90(5):S34.

29. Rizvi NA, Gettinger SN, Horn L, Gandhi L, Spigel DR, Powderly JD, Heist RS, Carvajal RD, Jackman DM, Sequist LV et al: Nivolumab (anti-PD-1; BMS-936558, ONO-4538) in patients with advanced non-small cell lung cancer (NSCLC): Survival and clinical activity by subgroup analysis. Journal of Thoracic Oncology 2014, 9(9):S152.

30. Brahmer JR, Horn L, Antonia SJ, Spigel D, Gandhi L, Sequist LV, Sankar V, Ahlers CM, Wigginton JM, Kollia G et al: Nivolumab (anti-PD-1; BMS-936558; ONO- 4538) in patients with non-small cell lung cancer (NSCLC): Overall survival and long-term safety in a phase 1 trial. Journal of Thoracic Oncology 2013, 8:S365-S366.

31. Goldman JW, Crino L, Vokes EE, Holgado E, Reckamp KL, Pluzanski A, Spigel DR, Kohlhaeufl M, Garassino MC, Chow LQM et al: Nivolumab (nivo) in patients (pts) with advanced (adv) NSCLC and central nervous system (CNS) metastases (mets). Journal of Clinical Oncology 2016, 34.

32. Gettinger SN, Horn L, Ramalingam SS, Spigel DR, Paz-Ares L, Paik P, Reck M, Reckamp K, Mazières J, Stinchcombe T et al: Nivolumab (NIVO) safety profile: Summary of findings from trials in patients (PTS) with advanced squamous (SQ) non-small cell lung cancer (NSCLC). European Journal of Cancer 2015, 51:S631.

33. Borghaei H, Brahmer JR, Horn L, Ready N, Steins M, Felip E, PazAres LG, Arrieta O, Barlesi F, Antonia SJ et al: Nivolumab (nivo) vs docetaxel (doc) in patients (pts) with advanced NSCLC: CheckMate 017/057 2-y update and exploratory cytokine profile analyses. Journal of Clinical Oncology 2016, 34.

34. Ulmeanu R, Antohe I, Anisie E, Antoniu S: Nivolumab for advanced non-small cell lung cancer: an evaluation of a phase III study. Expert review of anticancer therapy 2016, 16(2):165-167.

35. Lena H, Rizvi NA, Wolf J, Cappuzzo F, Zalcman G, Baas P, Mazieres J, Farsaci B, Blackwood-Chirchir MA, Ramalingam S: Nivolumab in patients (pts) with advanced refractory squamous (SQ) non-small cell lung cancer (NSCLC): 2-year follow-up from CheckMate 063 and exploratory cytokine profiling analyses. Journal of Thoracic Oncology 2016, 11(4):S115-S116.

36. Brahmer JR, Horn L, Gandhi L, Spigel DR, Antonia SJ, Rizvi NA, Powderly JD, Heist RS, Carvajal RD, Jackman DM et al: Nivolumab in patients with advanced non-small-cell lung cancer (NSCLC): Survival and clinical activity by subgroup analysis. Asia-Pacific Journal of Clinical Oncology 2014, 10:157.

37. Brahmer J, Reckamp KL, Baas P, Crino L, Eberhardt WE, Poddubskaya E, Antonia S, Pluzanski A, Vokes EE, Holgado E et al: Nivolumab versus Docetaxel in Advanced Squamous-Cell Non-Small-Cell Lung Cancer. The New England journal of medicine 2015, 373(2):123-135.

38. Bauer TM, McCleod M, Chandler JC, Blumenschein GR, Schwartzberg LS, Burris H, Waterhouse D, Jotte RM, Hussein M, Spigel DR et al: An ongoing phase IIIb/IV safety trial of nivolumab (NIVO) in patients (pts) with advanced or metastatic non-smallcell lung cancer (NSCLC) who progressed after receiving 1 or more prior systemic regimens. Journal of Clinical Oncology 2015, 33(15).

39. Rizvi NA, Garon EB, Leighl N, Hellmann MD, Patnaik A, Gandhi L, Eder JP, Rangwala RA, Lubiniecki G, Zhang J et al: Optimizing PDL1 as a biomarker of response with pembrolizumab (pembro; MK-3475) as first-line therapy for PDL1-positive metastatic non-small cell lung cancer (NSCLC): Updated data from KEYNOTE-001. Journal of Clinical Oncology 2015, 33(15).

40. Lee JS, Lee DH, Piperdi B, Zhang J, Lubiniecki GM, Ahn MJ: Patients (Pts) with advanced NSCLC from Korea treated with pembrolizumab (Pembro) in KEYNOTE-001. Annals of Oncology 2015, 26:ix141.

41. Fløtten Ø, Garon EB, Arkenau HT, Hui R, Gandhi L, Felip E, Lena H, Cappuzzo F, Horn L, Gubens M et al: Pembrolizumab 2 mg/kg Q3W for previously treated, PD-L1-positive advanced nsclc. Journal of Thoracic Oncology 2015, 10(9):S270.

42. Leighl N, Gandhi L, Hellmann MD, Horn L, Ahn MJ, Garon EB, Hui R, Ramalingam SS, Zhang J, Lubiniecki G et al: Pembrolizumab for NSCLC: Immune-mediated adverse events and corticosteroid use. Journal of Thoracic Oncology 2015, 10(9):S233.

43. Lim SH, Sun JM, Lee SH, Ahn JS, Park K, Ahn MJ: Pembrolizumab for the treatment of non-small cell lung cancer. Expert Opinion on Biological Therapy 2016, 16(3):397-406.

44. Zalcman G, Rizvi NA, Lena H, Wolf J, Mazieres J, Antonia SJ, Minenza E, Planchard D, Lestini BJ, Ramalingam SS: Phase 2 study of nivolumab (anti-programmed death-1 [PD-1]) in patients (PTS) with advanced, refractory squamous (SQ) non-small cell lung cancer (NSCLC). Annals of Oncology 2015, 26:i32-i33.

45. Rechamp K, Spigel DR, Rizvi N, Poddubskaya E, West H, Eberhardt W, Baas P, Antonia SJ, Pluzanski A, Vokes EE et al: Phase 3, randomized trial (checkmate 017) of nivolumab (NIVO) vs docetaxel in advanced squamous (SQ) cell non-small cell lung cancer (NSCLC). In: Asia-Pacific Journal of Clinical Oncology ( varpagings). vol. 11; 2015: 130.

46. Horn L, Brahmer J, Reck M, Borghaei H, Spigel DR, Steins M, Ready NE, Chow LQ, Vokes EE, Felip E et al: Phase 3, randomized trial (CheckMate 057) of nivolumab (NIVO) vs docetaxel (DOC) in advanced non-squamous (non-SQ) non-small cell lung cancer (NSCLC): Subgroup analyses and patient reported outcomes (PROs). In: European Journal of Cancer ( varpagings). vol. 51; 2015: S599.

47. Sakai H, Nishio M, Hida T, Nakagawa K, Nogami N, Atagi S, Takahashi T, Nokihara H, Saka H, Takenoyama M et al: Phase II studies of nivolumab in patients with advanced squamous (SQ) or non-squamous (NSQ) non-small cell lung cancer (NSCLC). European Journal of Cancer 2015, 51:S110-S111.

48. Ramalingam SS, Mazières J, Planchard D, Stinchcombe TE, Dy GK, Antonia SJ, Horn L, Lena H, Minenza E, Mennecier B et al: Phase II study of nivolumab (anti-PD-1, BMS-936558, ONO-4538) in Patients with advanced, refractory squamous non-small cell lung cancer: Metastatic non-small cell lung cancer. International Journal of Radiation Oncology Biology Physics 2014, 90(5):1266-1267.

49. Durm G, Hanna N: A phase II trial of concurrent chemoradiation with consolidation pembrolizumab in unresectable stage III non-small cell lung cancer. Journal of Thoracic Oncology 2015, 10(9):S569-S570.

50. Goldberg SB, Gettinger SN, Mahajan A, Herbst R, Chiang A, Tsiouris AJ, Vortmeyer A, Jilaveanu L, Speaker S, Madura M et al: A phase II trial of pembrolizumab for untreated brain metastases from non-small cell lung cancer. Journal of Thoracic Oncology 2015, 10(9):S234-S235.

51. Spigel DR, Reckamp KL, Rizvi NA, Poddubskaya E, West HJ, Eberhardt WEE, Baas P, Antonia SJ, Pluzanski A, Vokes EE et al: A phase III study (CheckMate 017) of nivolumab (NIVO; antiprogrammed death-1 [PD-1]) vs docetaxel (DOC) in previously treated advanced or metastatic squamous (SQ) cell non-small cell lung cancer (NSCLC). In: Journal of clinical oncology. vol. 33; 2015.

52. Paz-Ares L, Horn L, Borghaei H, Spigel DR, Steins M, Ready N, Chow LQM, Vokes EE, Felip E, Holgado E et al: Phase III, randomized trial (CheckMate 057) of nivolumab (NIVO) versus docetaxel (DOC) in advanced non-squamous cell (non-SQ) non-small cell lung cancer (NSCLC). Journal of Clinical Oncology 2015, 33(18).

53. Garon EB, Balmanoukian A, Hamid O, Hui R, Gandhi L, Leighl N, Gubens MA, Goldman J, Lubiniecki GM, Lunceford J et al: Preliminary clinical safety and activity of MK-3475 monotherapy for the treatment of previously treated patients with non-small cell lung cancer (NSCLC). Journal of Thoracic Oncology 2013, 8:S364-S365.

54. Garon EB, Leighl NB, Rizvi NA, Blumenschein GR, Balmanoukian AS, Eder JP, Goldman JW, Hui R, Soria JC, Gangadhar TC et al: Safety and clinical activity of MK-3475 in previously treated patients (pts) with non-small cell lung cancer (NSCLC). Journal of Clinical Oncology 2014, 32(15).

55. Waterhouse DM, Horn L, Reynolds CH, Spigel DR, Chandler JC, Mekhail T, Mohamed MK, Creelan BC, Blankstein KB, Nikolinakos P et al: Safety profile of nivolumab administered as 30-minute (min) infusion: Analysis of data from CheckMate 153. Journal of Clinical Oncology 2016, 34.

56. Brahmer JR, Horn L, Antonia SJ, Spigel DR, Gandhi L, Sequist LV, Sankar V, Ahlers CM, Wigginton JM, Kollia G et al: Survival and long-term follow-up of the phase I trial of nivolumab (Anti-PD-1; BMS-936558; ONO-4538) in patients (pts) with previously treated advanced non-small cell lung cancer (NSCLC). Journal of Clinical Oncology 2013, 31(15).

57. Chatterjee M, Turner DC, Felip E, Lena H, Cappuzzo F, Horn L, Garon EB, Hui R, Arkenau HT, Gubens MA et al: Systematic evaluation of pembrolizumab dosing in patients with advanced non-small-cell lung cancer. Annals of oncology : official journal of the European Society for Medical Oncology / ESMO 2016, 27(7):1291-1298.

58. Smith DA, Vansteenkiste JF, Fehrenbacher L, Park K, Mazieres J, Rittmeyer A, Artal-Cortes A, Lewanski CR, Braiteh FS, Yi J et al: Updated survival and biomarker analyses of a randomized phase II study of atezolizumab vs docetaxel in 2L/3L NSCLC (POPLAR). Journal of Clinical Oncology 2016, 34.
