# Supplementary material for: Immune-related adverse events associated with programmed cell death protein-1 and programmed cell death ligand 1 inhibitors for non-small cell lung cancer: a PRISMA systematic review and meta-analysis
Source: BMC Cancer. 2019 Jun 10;19:558. doi: 10.1186/s12885-019-5701-6 (PMC6558759; doi:10.1186/s12885-019-5701-6)
Supplement: Supplementary file 3 — Figure S1. to Figure S2. Global irAEs (all grade and severe grade) associated with anti-PD-1 and anti-PD-L1 drugs. Figure S3. to Figure S4. Global irAEs (all grade and severe grade) associated with pembrolizumab and durvalumab. Figure S5 to Figure S43. Organ-specific (i.e., skin, endocrine, gastrointestinal, hepatic, and renal diseases) irAEs (all grade and high grade) associated with anti-PD-1 and anti-PD-L1, anti-PD-1, nivolumab, pembrolizumab, anti-PD-L1, atezolizumab, and durvalumab at all dosages. Figure S44. Death related to irAEs. (DOCX 9781 kb) [file 12885_2019_5701_MOESM3_ESM.docx]

**Results**

**Meta-analysis**

**1-** All immune related adverse events


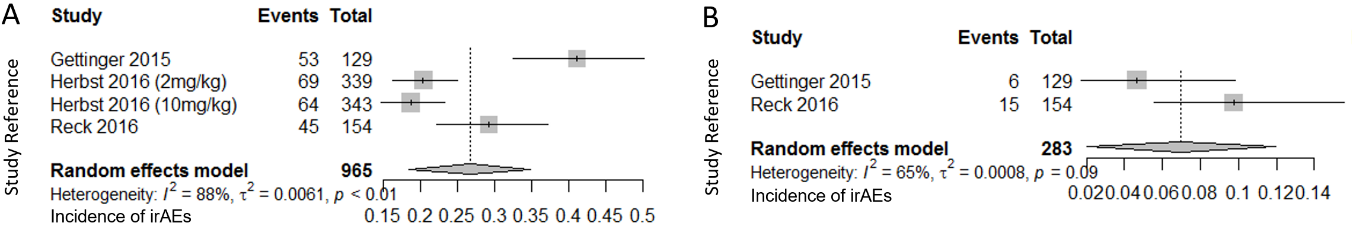


**Supplementary fig. 1** - Incidence of global irAEs with anti-PD-1 all dosage, all-grade (A) and severe grade (B).


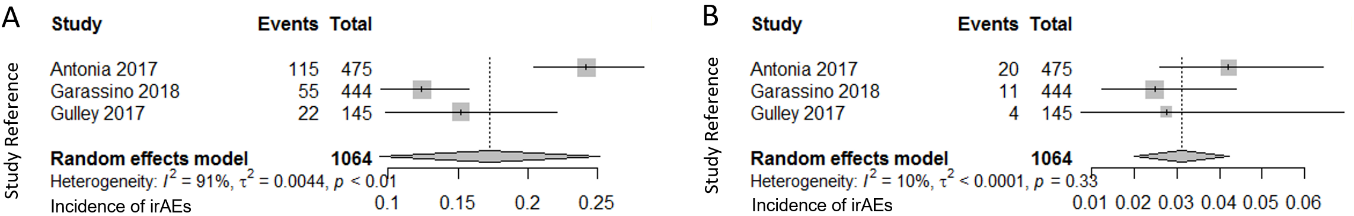


**Supplementary fig. 2** - Incidence of global irAEs with anti-PD-L1 all dosage, all-grade (**A**) and severe grade (**B**).


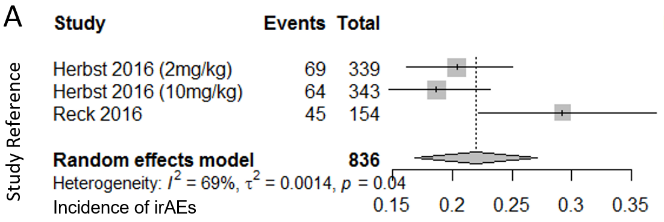


**Supplementary fig. 3** - Incidence of global irAEs with pembrolizumab all dosage, all-grade (**A**).


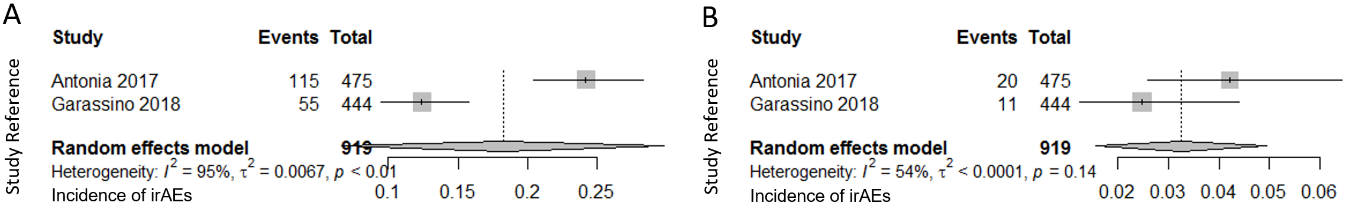


**Supplementary fig. 4** - Incidence of global irAEs with durvalumab all dosage, all-grade (**A**) and severe grade (**B**).

**
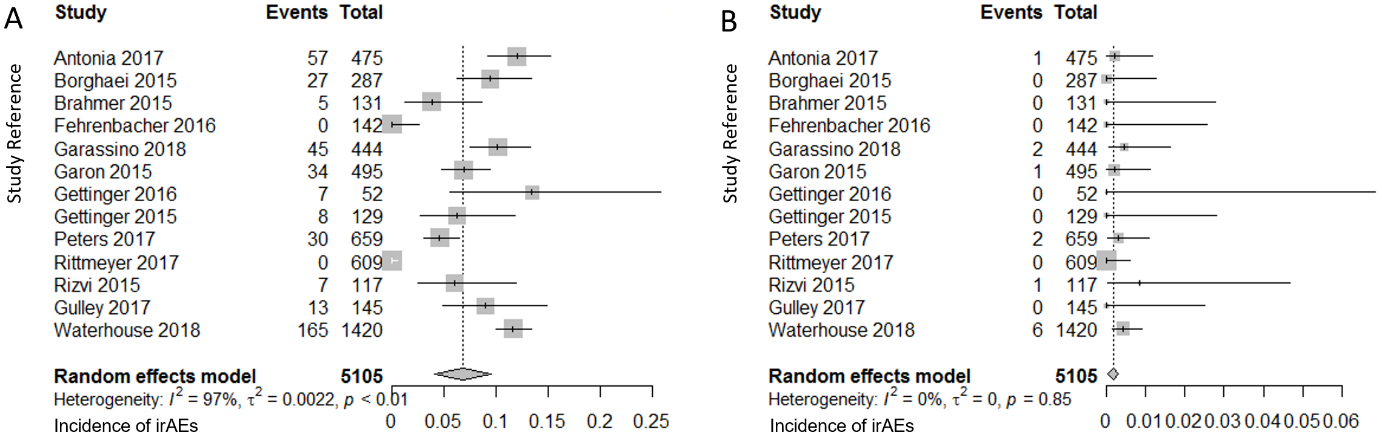
**

**Supplementary fig. 5 -** Incidence of endocrinologic irAEs with anti-PD-1 and anti-PD-L1, all-grade (A) and severe grade (B).

**
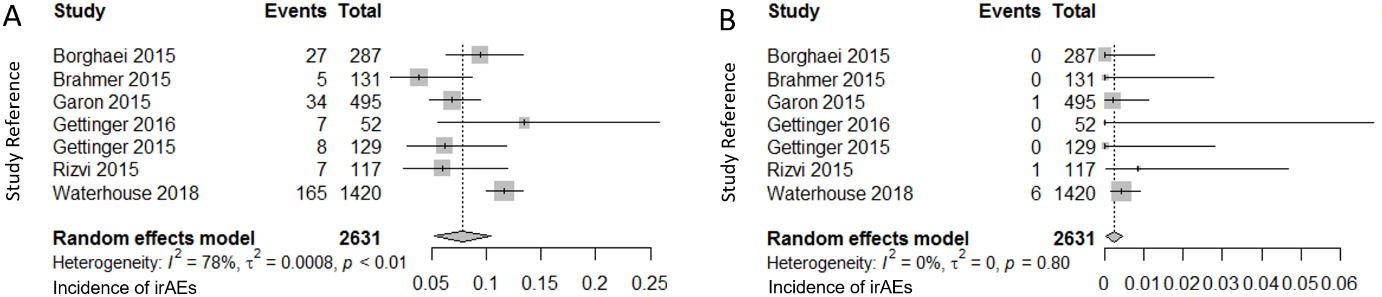
**

**Supplementary fig. 6 -** Incidence of endocrinologic irAEs with anti-PD-1, all-grade (A) and severe grade (B).

**
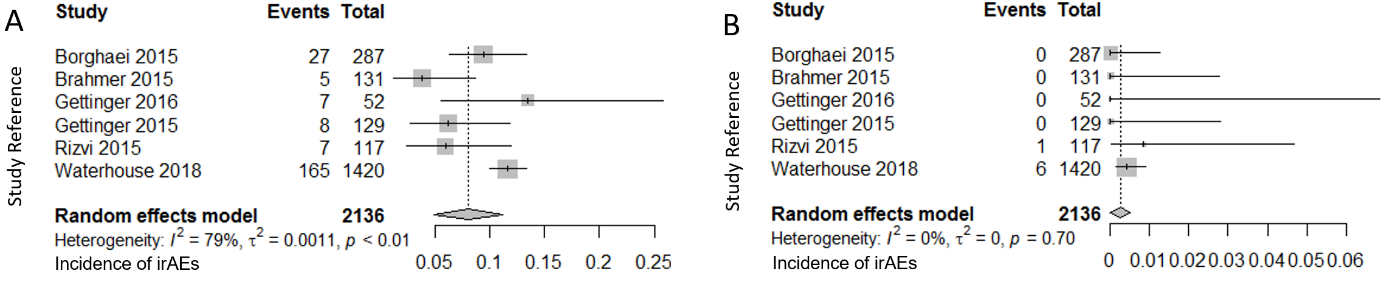
**

**Supplementary fig. 7 -** Incidence of endocrinologic irAEs with nivolumab all dosage, all-grade (A) and severe grade (B).

**
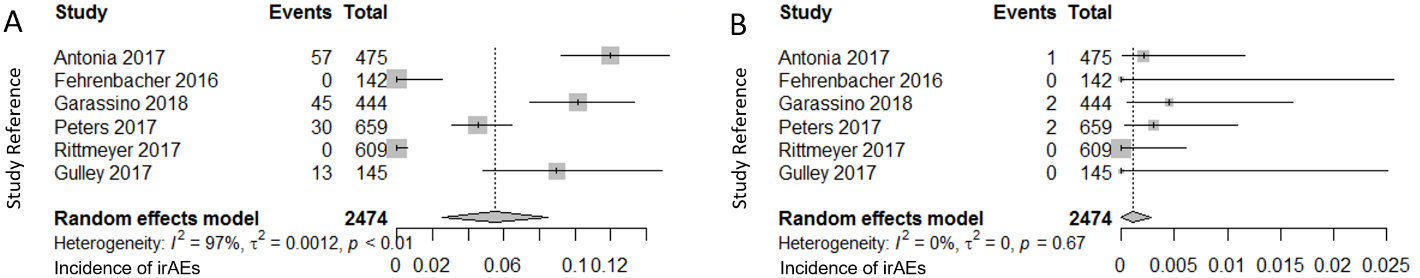
**

**Supplementary fig. 8 -** Incidence of endocrinologic irAEs with anti-PD-L1, all-grade (A) and severe grade (B).

**
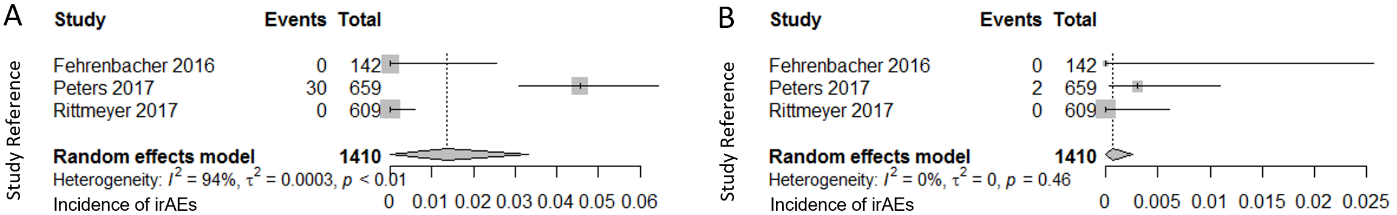
**

**Supplementary fig. 9 -** Incidence of endocrinologic irAEs with atezolizumab all dosage, all-grade (A) and severe grade (B).

**
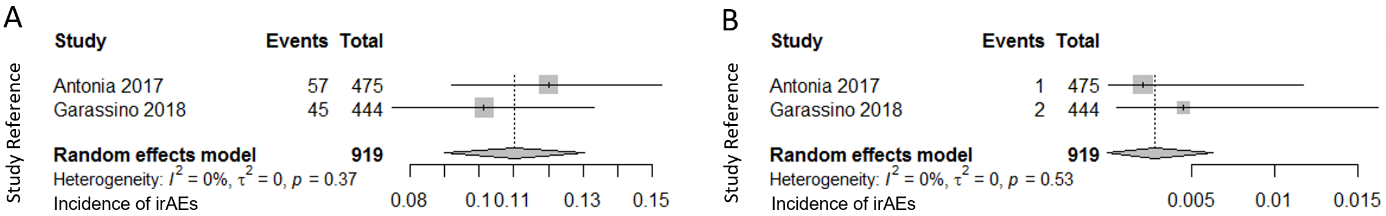
**

**Supplementary fig. 10 -** Incidence of endocrinologic irAEs with durvalumab all dosage, all-grade (A) and severe grade (B).

**
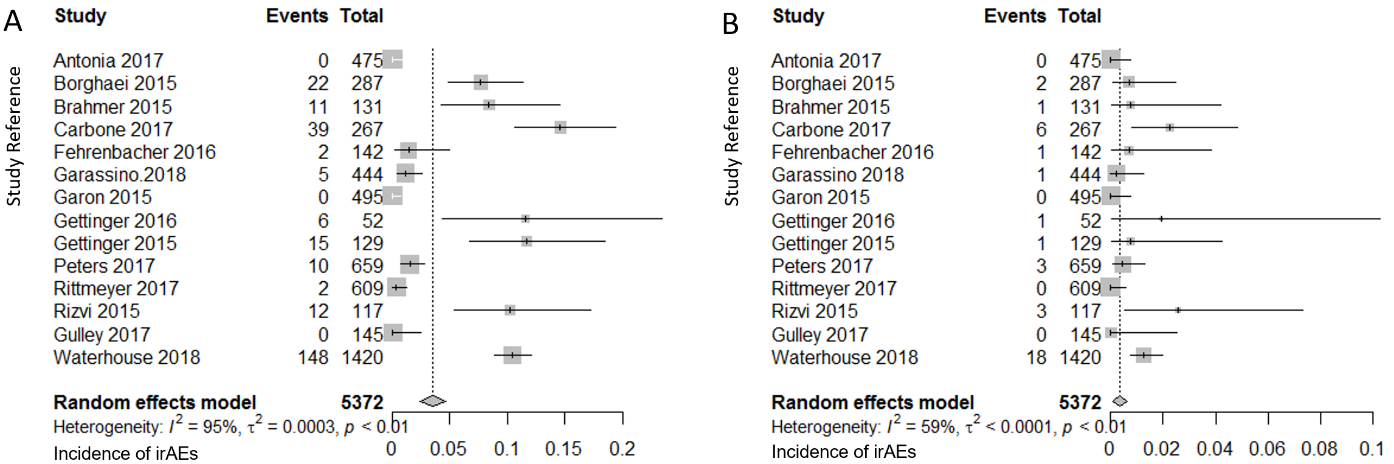
**

**Supplementary fig. 11 -** Incidence of gastro intestinal irAEs with anti-PD-1 and anti-PD-L1, all-grade (A) and severe grade (B).

**
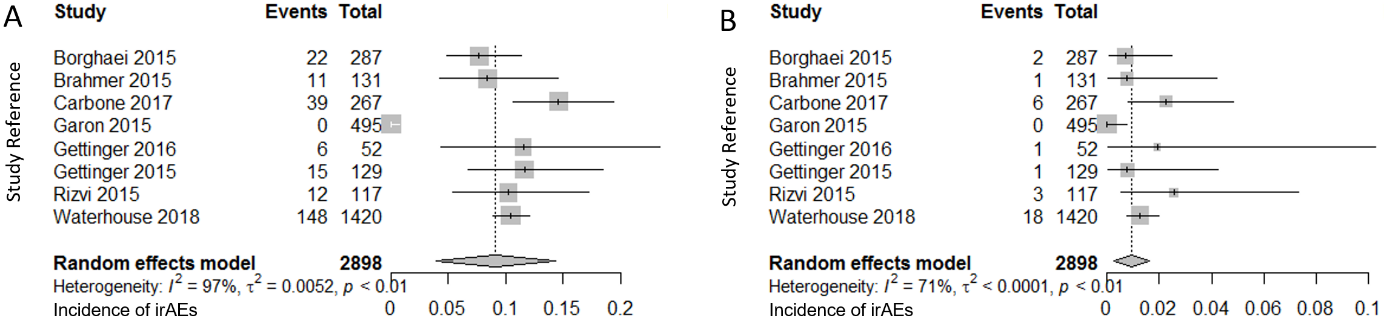
**

**Supplementary fig. 12 -** Incidence of gastro intestinal irAEs with anti-PD-1, all-grade (A) and severe grade (B).

**
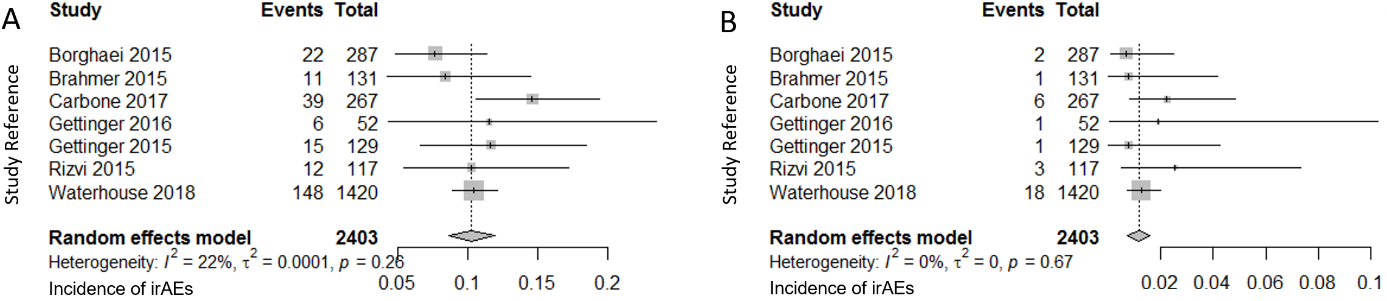
**

**Supplementary fig. 13 -** Incidence of gastro intestinal irAEs with nivolumab, all-grade (A) and severe grade (B).

**
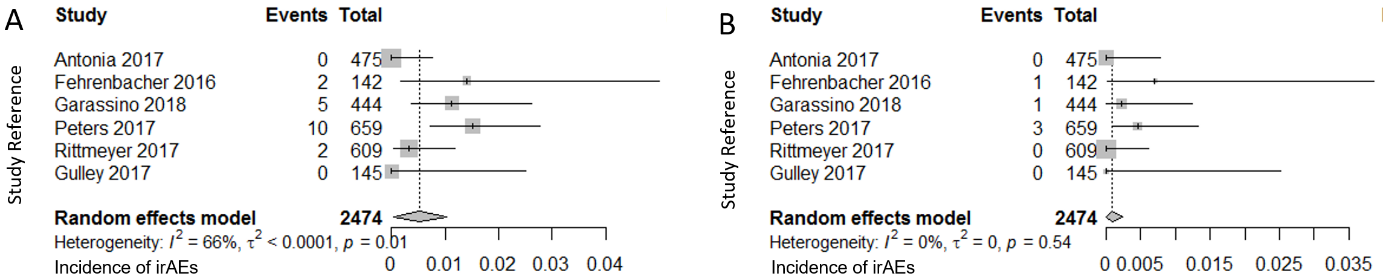
**

**Supplementary fig. 14 -** Incidence of gastro intestinal irAEs with anti-PD-L1, all-grade (A) and severe grade (B).

**
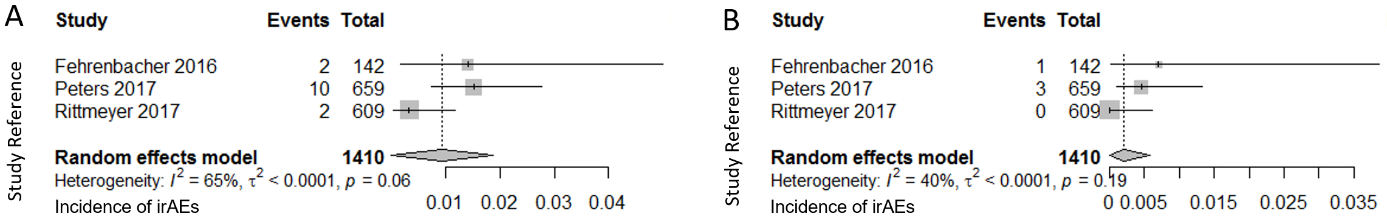
**

**Supplementary fig. 15 -** Incidence of gastro intestinal irAEs with atezolizumab, all-grade (A) and severe grade (B).

**
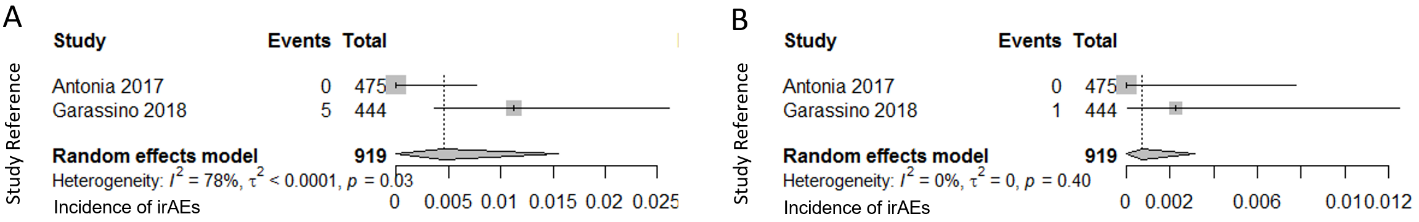
**

**Supplementary fig. 16 -** Incidence of gastro intestinal irAEs with durvalumab, all-grade (A) and severe grade (B).

**
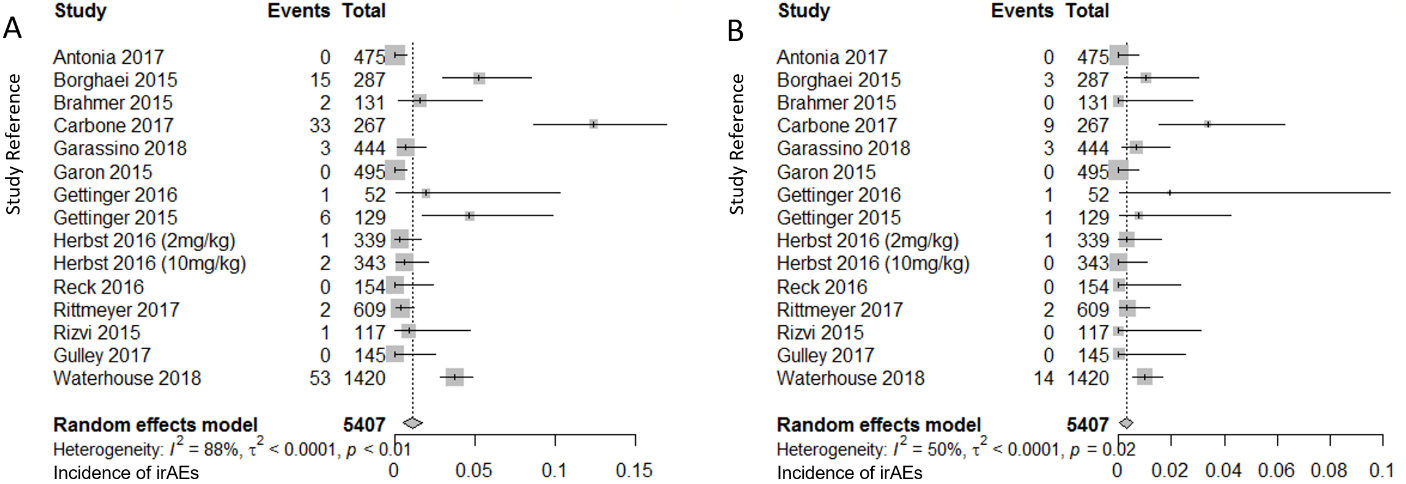
**

**Supplementary fig. 17 -** Incidence of hepatic irAEs with anti-PD-1 and anti-PD-L1, all-grade (A) and severe grade (B).

**
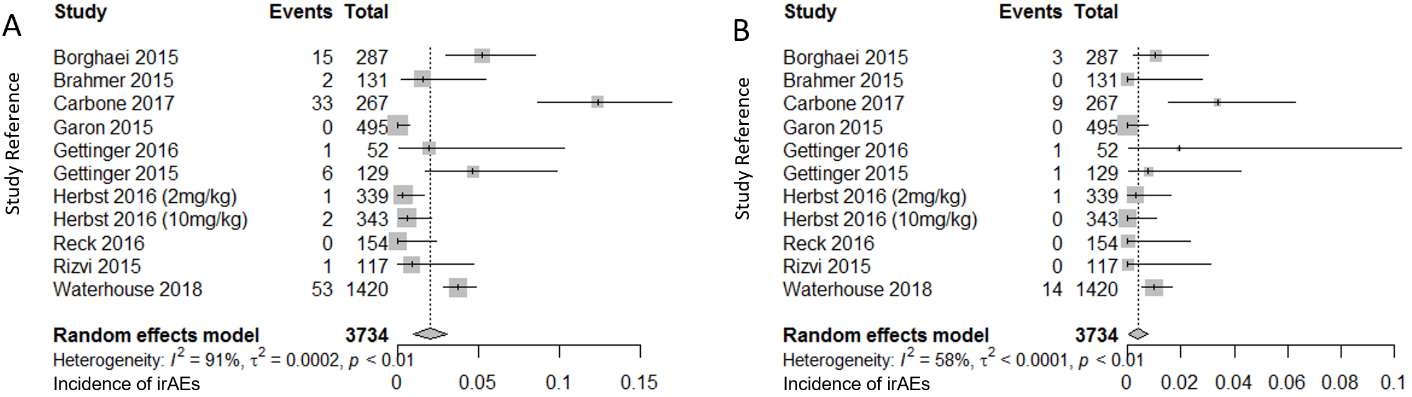
**

**Supplementary fig. 18 -** Incidence of hepatic irAEs with anti-PD-1, all-grade (A) and severe grade (B).

**
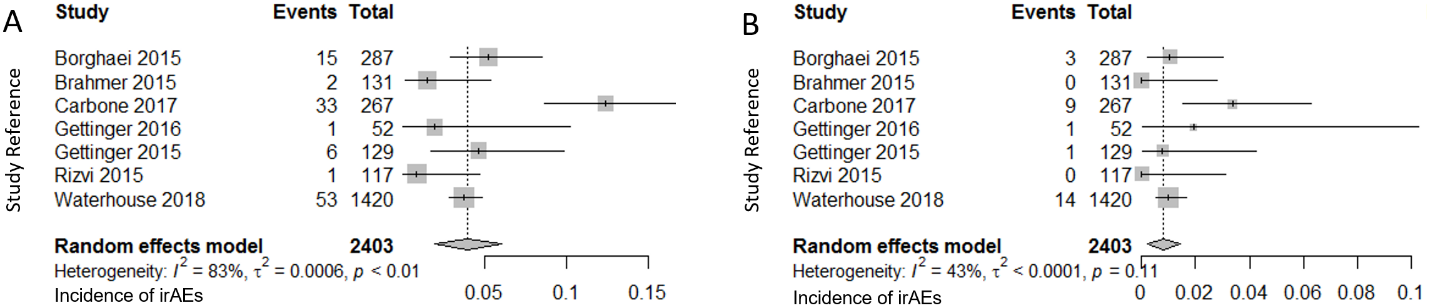
**

**Supplementary fig. 19 -** Incidence of hepatic irAEs with nivolumab, all-grade (A) and severe grade (B).

**
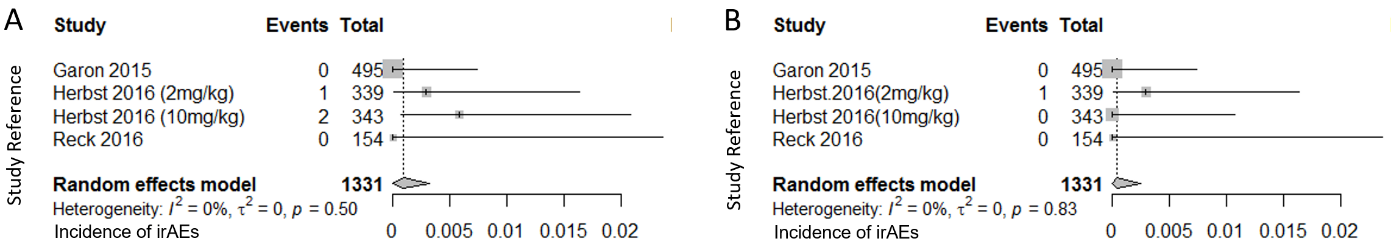
**

**Supplementary fig. 20 -** Incidence of hepatic irAEs with pembrolizumab, all-grade (A) and severe grade (B).

**
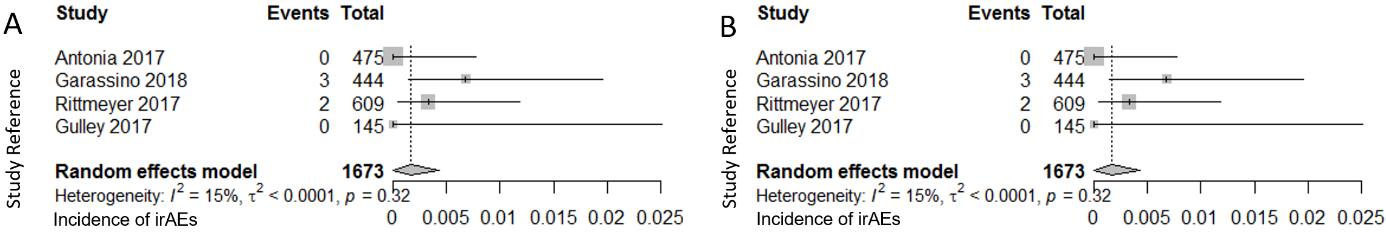
**

**Supplementary fig. 21 -** Incidence of hepatic irAEs with anti-PD-L1, all-grade (A) and severe grade (B).

**
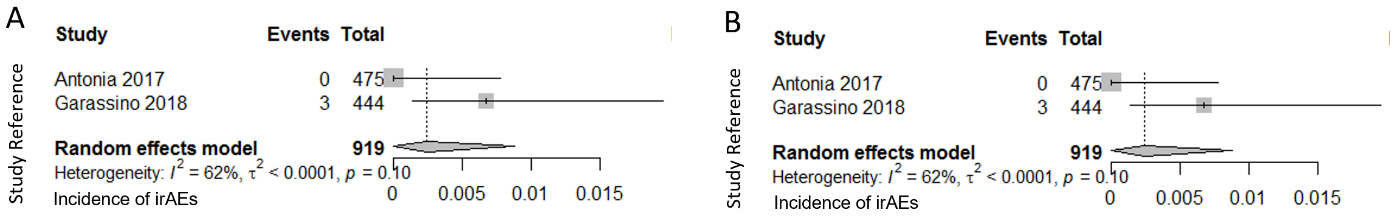
**

**Supplementary fig. 22 -** Incidence of hepatic irAEs with durvalumab, all-grade (A) and severe grade (B).

**
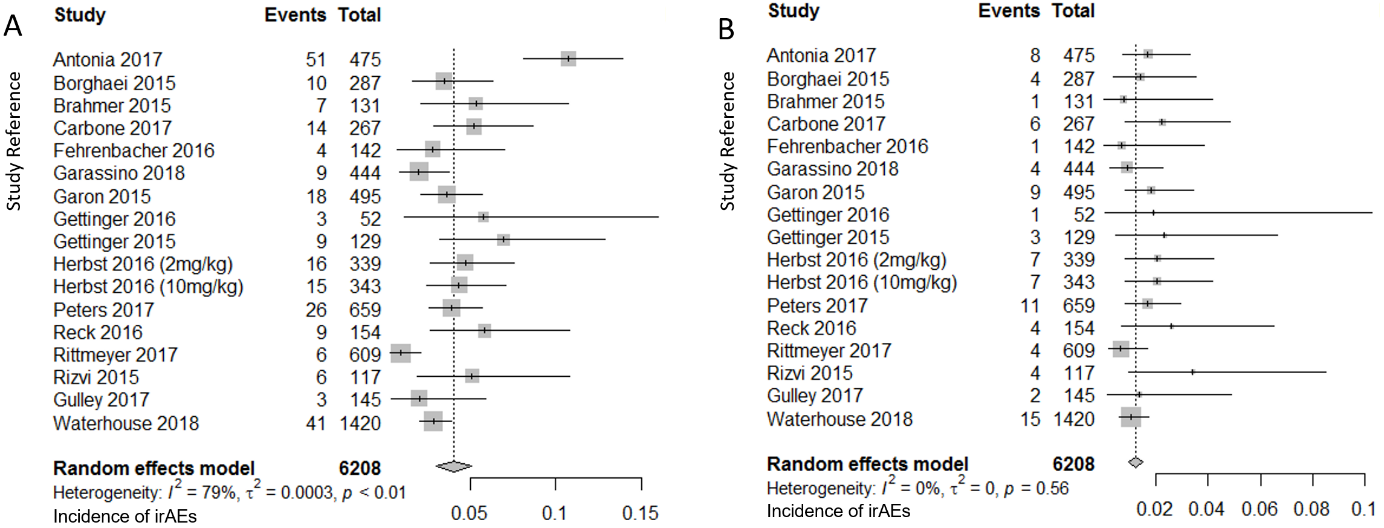
**

**Supplementary fig. 23 -** Incidence of pulmonary irAEs with anti-PD-1 and anti-PD-L1, all-grade (A) and severe grade (B).

**
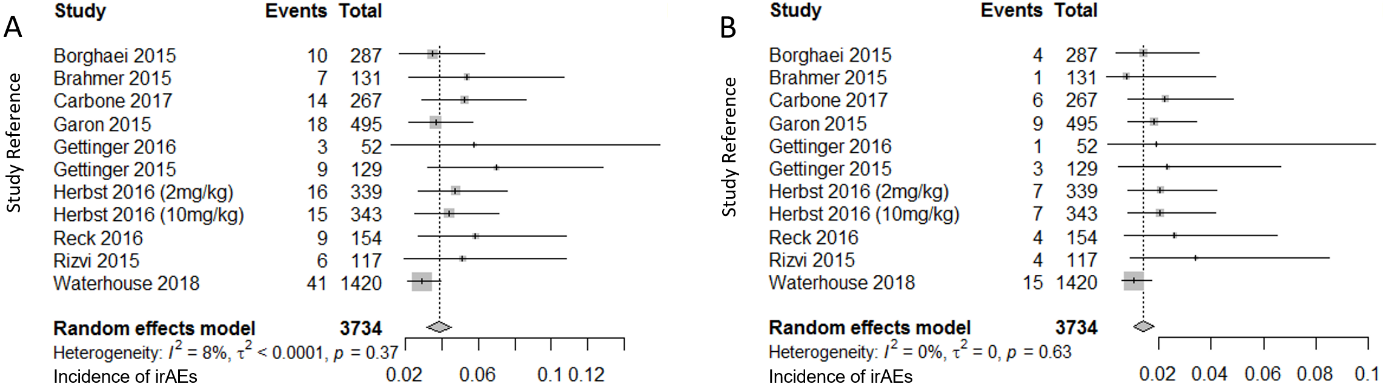
**

**Supplementary fig. 24 -** Incidence of pulmonary irAEs with anti-PD-1, all-grade (A) and severe grade (B).

**
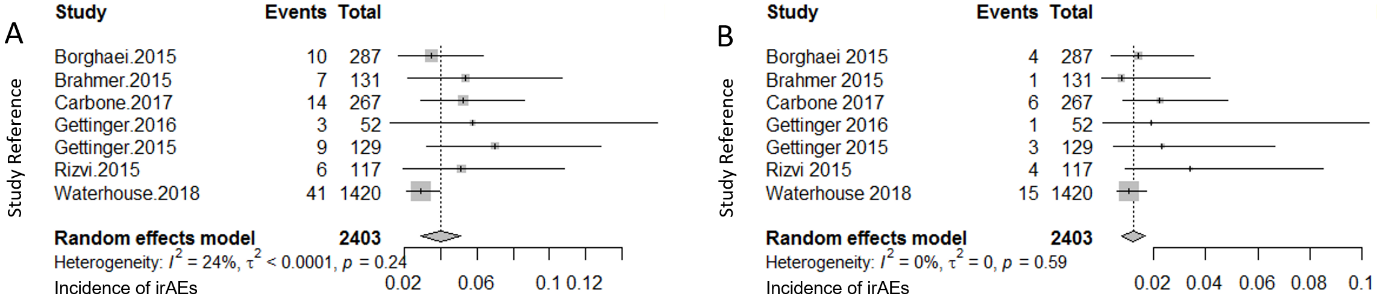
**

**Supplementary fig. 25 -** Incidence of pulmonary irAEs with nivolumab, all-grade (A) and severe grade (B).

**
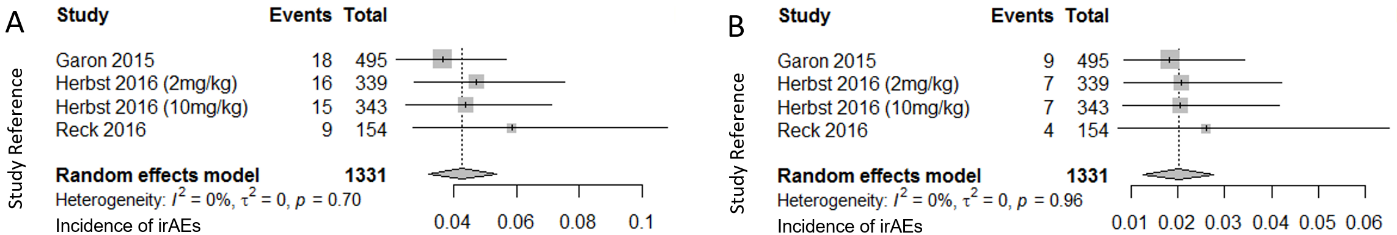
**

**Supplementary fig. 26 -** Incidence of pulmonary irAEs with pembrolizumab, all-grade (A) and severe grade (B).

**
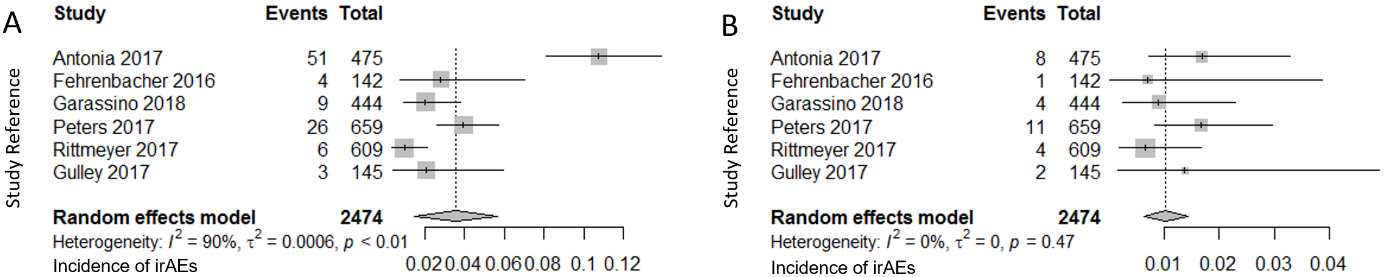
**

**Supplementary fig. 27 -** Incidence of pulmonary irAEs with anti-PD-L1, all-grade (A) and severe grade (B).

**
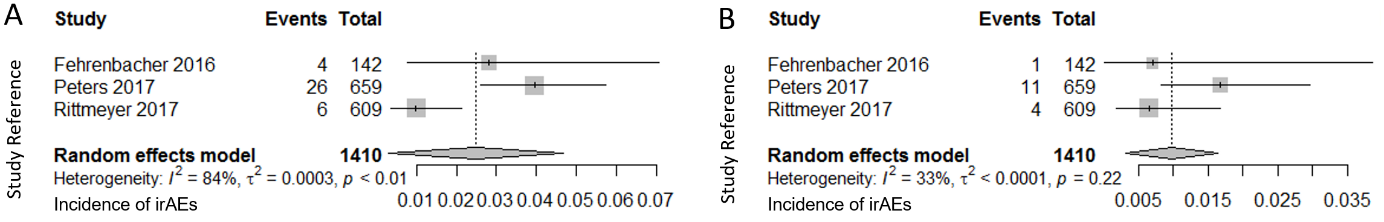
**

**Supplementary fig. 28 -** Incidence of pulmonary irAEs with atezolizumab, all-grade (A) and severe grade (B).

**
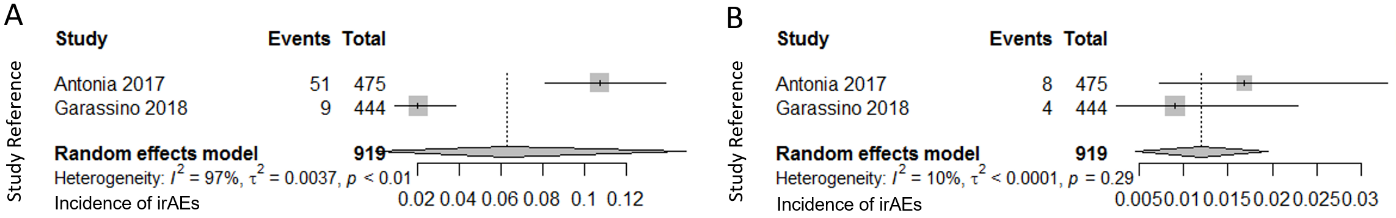
**

**Supplementary fig. 29 -** Incidence of pulmonary irAEs with durvalumab, all-grade (A) and severe grade (B).

**
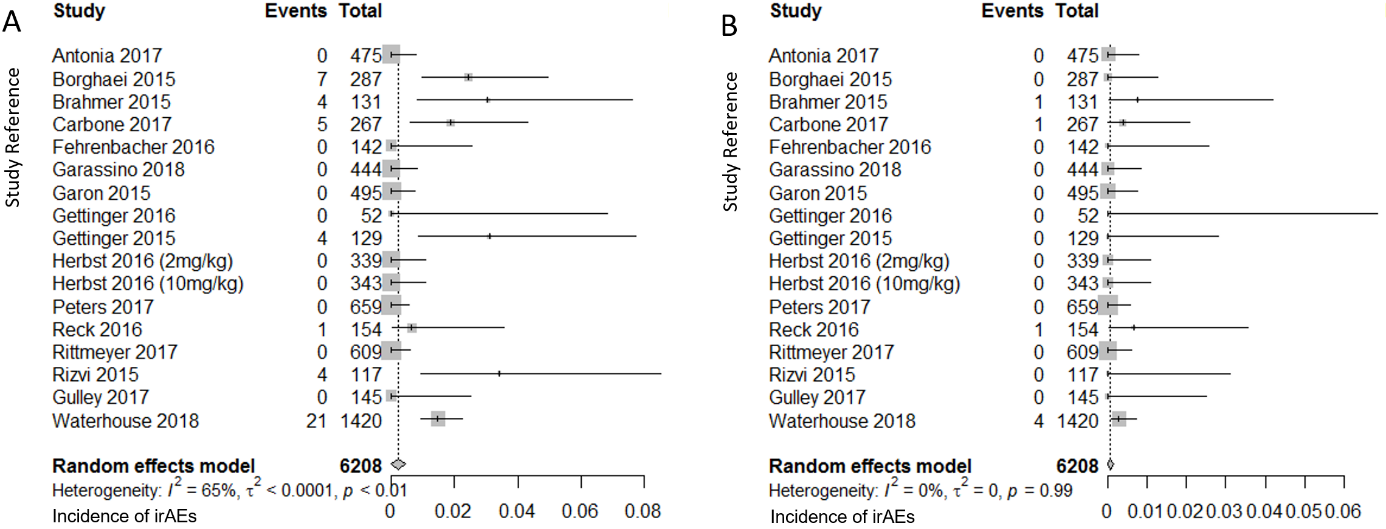
**

**Supplementary fig. 30 -** Incidence of renal irAEs with anti-PD-1 and anti-PD-L1, all-grade (A) and severe grade (B).

**
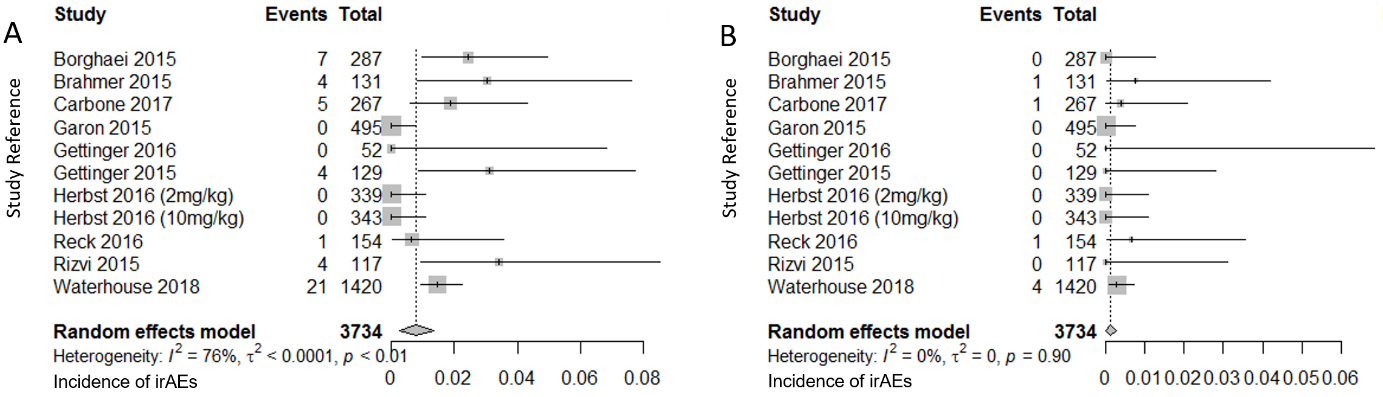
**

**Supplementary fig. 31 -** Incidence of renal irAEs with anti-PD-1, all-grade (A) and severe grade (B).

**
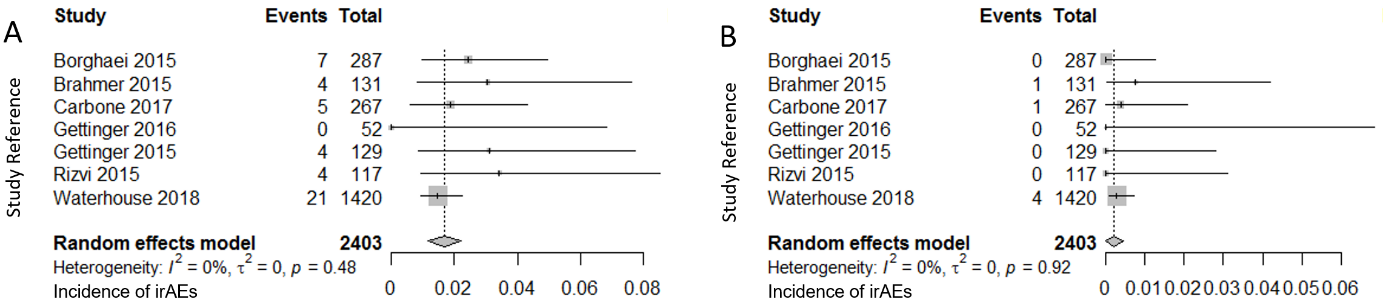
**

**
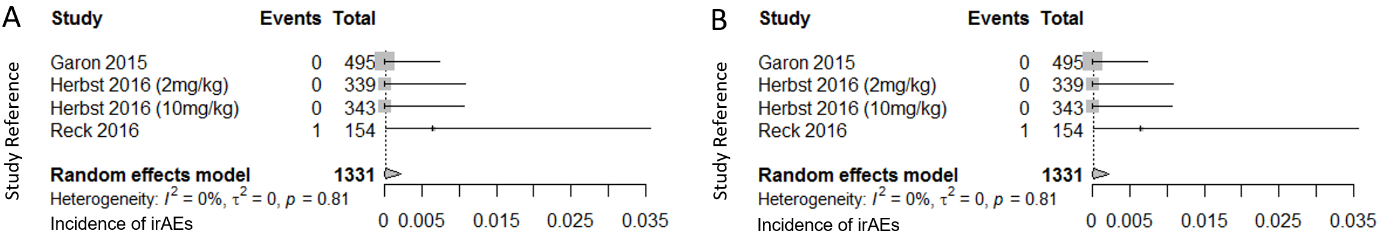
 Supplementary fig. 32 -** Incidence of renal irAEs with nivolumab, all-grade (A) and severe grade (B).

**Supplementary fig. 33 -** Incidence of renal irAEs with pembrolizumab, all-grade (A) and severe grade (B).

**
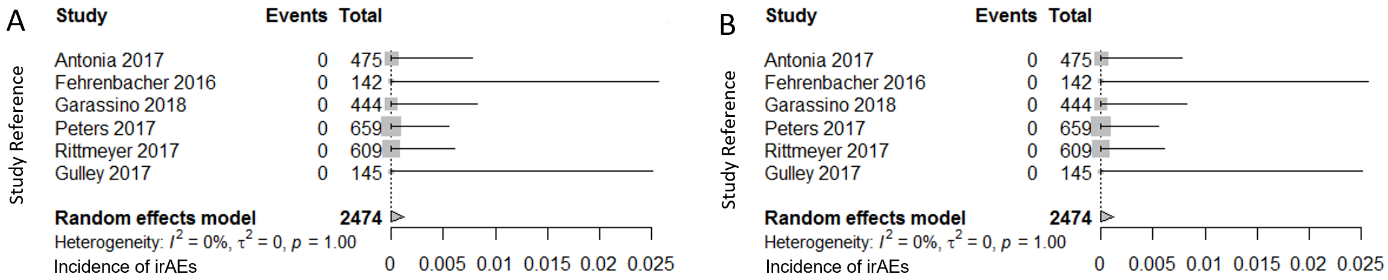
**

**Supplementary fig. 34 -** Incidence of renal irAEs with anti-PD-L1, all-grade (A) and severe grade (B).

**
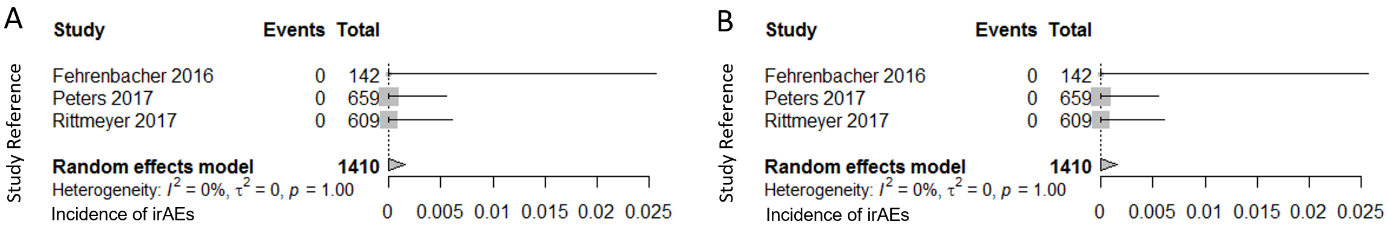
**

**Supplementary fig. 35 -** Incidence of renal irAEs with atezolizumab, all-grade (A) and severe grade (B).


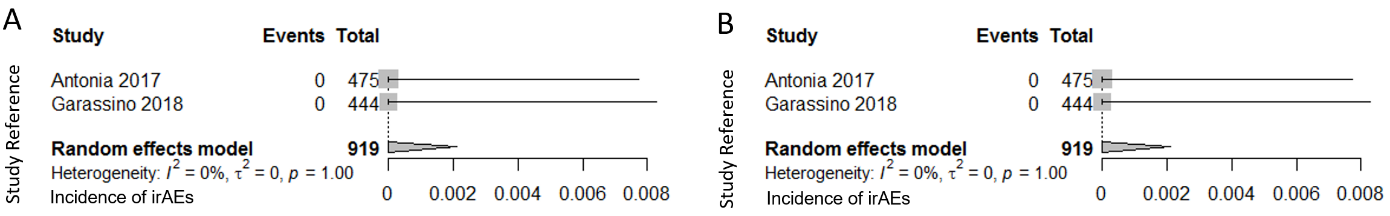


**Supplementary fig. 36 -** Incidence of renal irAEs with durvalumab, all-grade (A) and severe grade (B).

**
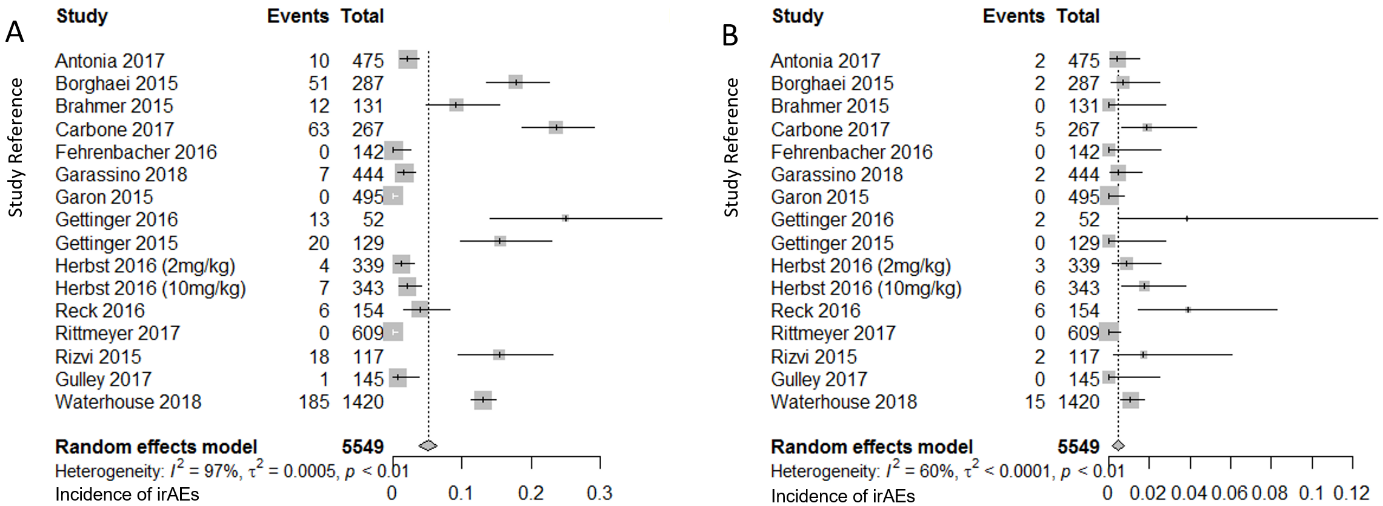
**

**Supplementary fig. 37 -** Incidence of cutaneous irAEs with anti-PD-1 and anti-PD-L1, all-grade (A) and severe grade (B).

**
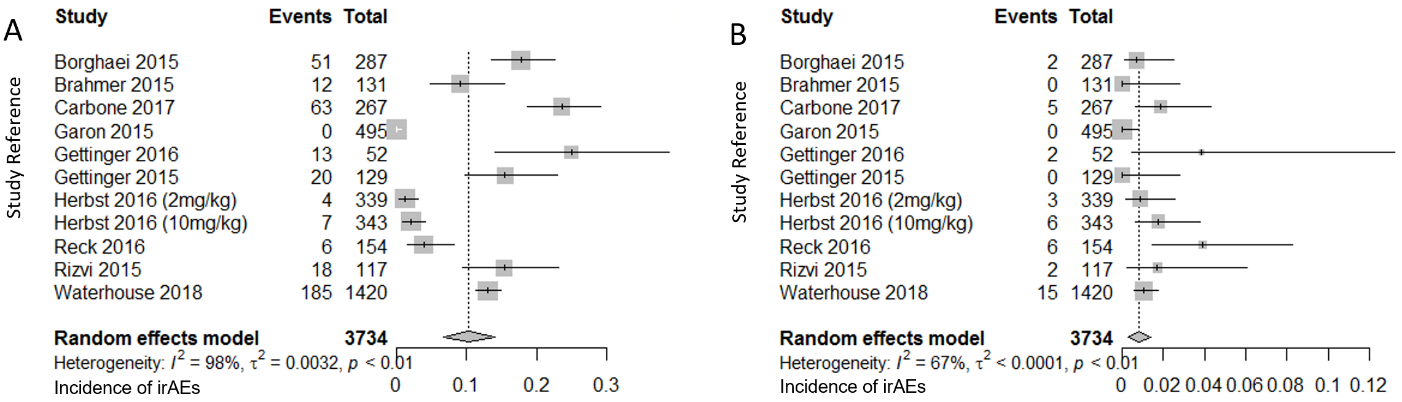
**

**Supplementary fig. 38 -** Incidence of cutaneous irAEs with anti-PD-1, all-grade (A) and severe grade (B).

**
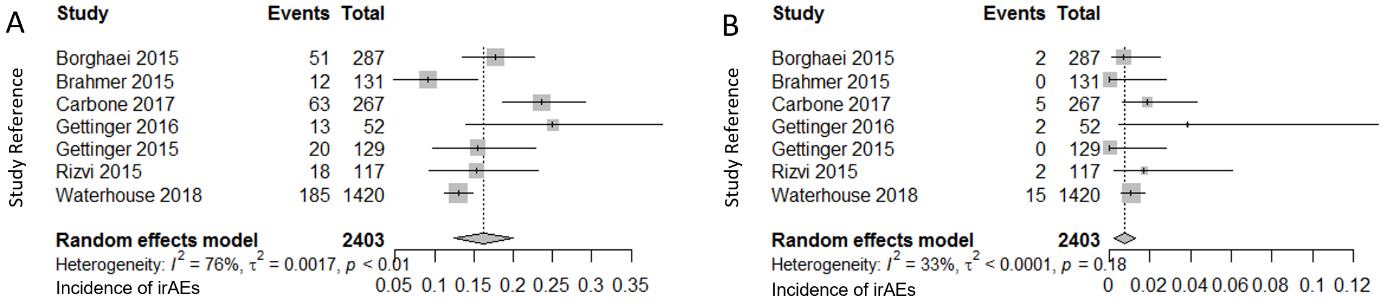
**

**Supplementary fig. 39 -** Incidence of cutaneous irAEs with nivolumab, all-grade (A) and severe grade (B).

**
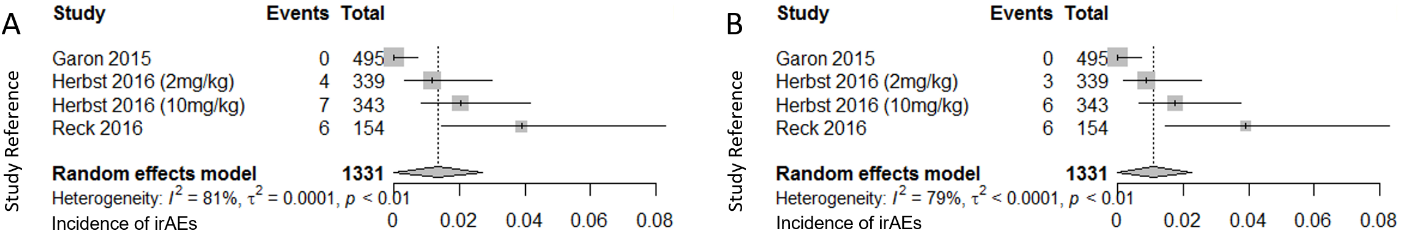
**

**Supplementary fig. 40 -** Incidence of cutaneous irAEs with pembrolizumab, all-grade (A) and severe grade (B).

**
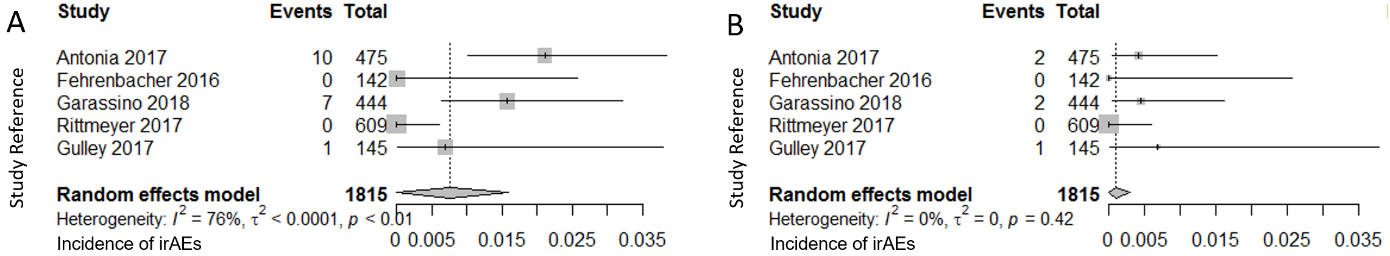
**

**Supplementary fig. 41 -** Incidence of cutaneous irAEs with anti-PD-L1, all-grade (A) and severe grade (B).

**
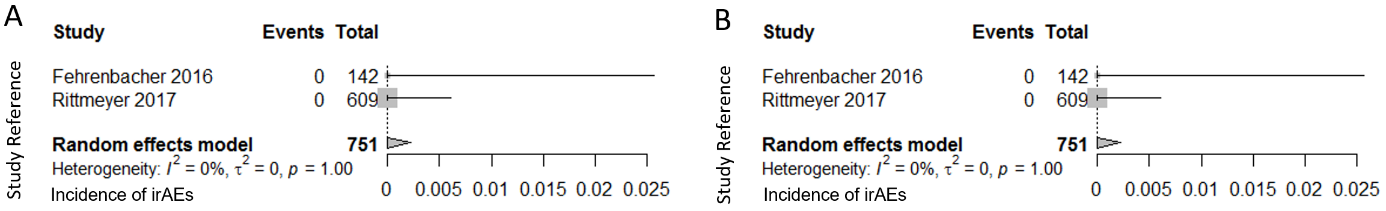
**

**Supplementary fig. 42 -** Incidence of cutaneous irAEs with atezolizumab, all-grade (A) and severe grade (B).

**
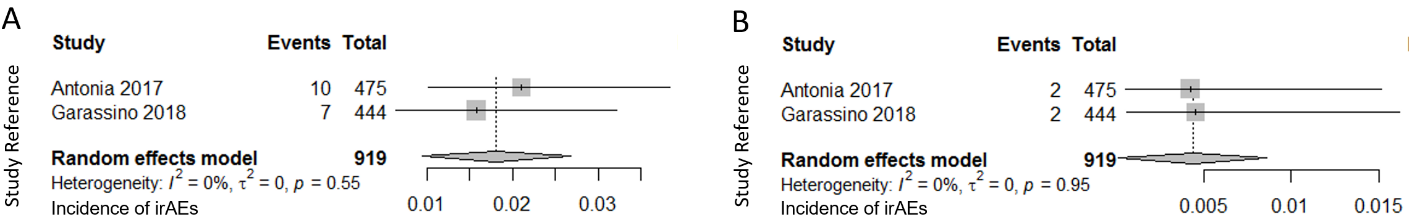
**

**Supplementary fig. 43 -** Incidence of cutaneous irAEs with durvalumab, all-grade (A) and severe grade (B).

**
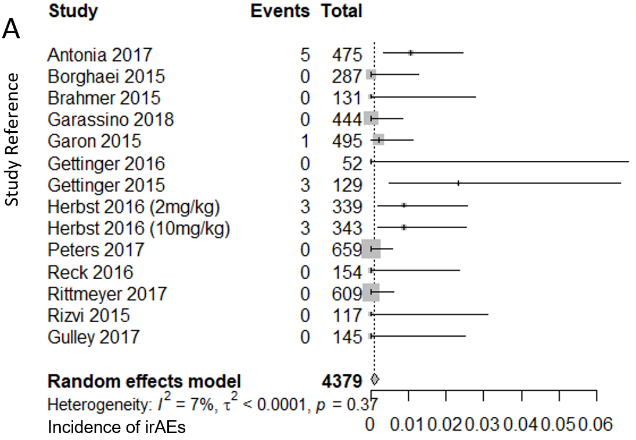
**

**Supplementary fig. 44 -** Incidence of death related to irAEs (A).
