# Supplementary material for: Immune-related adverse events associated with programmed cell death protein-1 and programmed cell death ligand 1 inhibitors for non-small cell lung cancer: a PRISMA systematic review and meta-analysis
Source: BMC Cancer. 2019 Jun 10;19:558. doi: 10.1186/s12885-019-5701-6 (PMC6558759; doi:10.1186/s12885-019-5701-6)
Supplement: Supplementary file 4 — Table S2. General characteristics of patients receiving anti-PD-1/anti-PD-L1 antibodies, as described in case reports (n = 35). Table S3. Organ-specific immune-related adverse events. (DOCX 32 kb) [file 12885_2019_5701_MOESM4_ESM.docx]

**Supplementary table 2** General characteristics of patients receiving anti-PD-1/anti-PD-L1 antibodies, as described in case reports (n=35)

| Age (y), mean±SD | 65.5±11.9 |
| --- | --- |
| Male sex, n (%) | 21 (60) |
| Personal history of AI disease, n (%) | 3 (9) |
| **Oncologic treatment, n (%)** |  |
| History of chemotherapy | 19 (54) |
| Unknown | 14 (40) |
| PD-1 treatment | 34 (97) |
| Nivolumab treatment | 26 (74) |
| Pembrolizumab treatment | 4 (11) |
| Unknown | 4 (11) |
| PD-L1 treatment | 1 (3) |
| Unknown | 1 (3) |
| **IRAE** |  |
| Endocrinopathy | 11 (31) |
| Gastroenterology and hepatology | 1 (3) |
| Neurology | 4 (11) |
| Hematology | 3 (9) |
| Nephrology | 5 (14) |
| Dermatology | 3 (9) |
| Cardiology | 5 (14) |
| Pulmonology | 6 (17) |
| No. of cycles, median (interquartile range) | 6 (2.75–11.25) |
| Onset of irAE (wk), median (interquartile range) | 10 (6–19.5) |
| Continued treatment (anti-PD-1/anti-PD-L1) | 10 (29) |
| **Evolution, n (%)** |  |
| Cancer |  |
| Progressive disease | 7 (20) |
| Oncologic response | 14 (40) |
| irAE |  |
| Healing | 25 (71) |
| Persistence | 4 (11) |
| Death | 2 (6) |
| Unknown | 4 (11) |

AI, auto-immune; irAE, immune-related adverse event; PD-1, programmed cell death protein-1; PD-L1, programmed cell death ligand 1; SD, standard deviation

**Supplementary table 3** Organ-specific immune-related adverse events

Endocrinopathy (n=11)

| Hypothyroidism | 6 (55) |
| --- | --- |
| Hyperthyroidism | 1 (9) |
| Thyroiditis | 1 (9) |
| Adrenal crisis | 1 (9) |
| Diabetes | 1 (9) |
| Fulminant type 1 diabetes | 1 (9) |
| No. of cycles, median (interquartile range) | 6 (4–6.5) |
| Weeks to irAE onset, median (interquartile range) | 12 (6–12) |
| **Treatment** |  |
| Steroids | 2 (18) |
| **Evolution** |  |
| Healing of irAE | 6 (55) |
| Persistence of irAE | 2 (18) |
| Unknown | 3 (27) |

Neurology (n=4)

| Cerebral vasculitis/encephalitis | 1 (25) |
| --- | --- |
| Myasthenia gravis | 3 (75) |
| No. of cycles, median (interquartile range) | 3 (3–3.5) |
| Weeks to irAE onset, median (interquartile range) | 7 (6.5–7.5) |
| **Treatment** |  |
| Steroids | 3 (75) |
| Plasmapheresis | 1 (25) |
| **Evolution** |  |
| Healing of irAE | 2 (50) |
| Persistence of irAE | 1 (25) |
| Death | 1 (25) |

Gastroenterology and Hepatology (n=1)

| Pancreatitis | | 1 (100) |
| --- | --- | --- |
| No. of cycles | | 2 |
| Weeks to irAE onset | 2 |  |
| **Treatment** | |  |
| Steroids | | 1 (100) |
| **Evolution** | |  |
| Healing of irAE | | 1 (100) |

Hematology (n=3)

| Agranulocytosis | | 1 (33) |
| --- | --- | --- |
| Warm-autoimmune hemolytic anemia | | 1 (33) |
| Immune thrombocytopenia | | 1 (33) |
| No. of cycles, median (interquartile range) | | 11 (7–15) |
| Weeks to irAE onset, median (interquartile range) | | 17 (7–23) |
| **Treatment** | |  |
| Steroids | 3 (100) |  |
| **Evolution** | |  |
| Healing of irAE | | 3 (100) |

Nephrology (n=5)

| Autoimmune nephritis | 1 (20) |
| --- | --- |
| Acute interstitial nephritis | 4 (80) |
| No. of cycles, median (interquartile range) | 22 (18–32) |
| Weeks to irAE onset, median (interquartile range) | 40 (24–54) |
| **Treatment** |  |
| Steroids | 4 (80) |
| **Evolution** |  |
| Healing of irAE | 4 (80) |
| Persistence of irAE | 1 (20) |

Dermatology (n=3)

| Rash | 1 (33) |
| --- | --- |
| Psoriasis | 1 (33) |
| Bullous pemphigoid | 1 (33) |
| No. of cycles, median (interquartile range) | 6 (3.75–7.25) |
| Weeks to irAE onset, median (interquartile range) | 8 (7–23) |
| **Treatment** |  |
| Steroids | 3 (100) |
| **Evolution** |  |
| Healing of irAE | 3 (100) |

Cardiology (n=5)

| Myocarditis | 2 (40) |
| --- | --- |
| Pericardial effusion | 2 (40) |
| Acute coronary syndrome | 1 (20) |
| No. of cycles, median (interquartile range) | 7 (4.25–9.5) |
| Weeks to irAE onset, median (interquartile range) | 14 (9–18.75) |
| **Treatment** |  |
| Steroids | 3 (60) |
| **Evolution** |  |
| Healing of irAE | 3 (60) |
| Death | 1 (20) |

Pulmonology (n=6)

| Pleural effusion | 1 (17) |
| --- | --- |
| Pneumonitis | 4 (67) |
| Interstitial pneumonia | 1 (17) |
| No. of cycles, median (interquartile range) | 6.5 (3.75–10) |
| Weeks to irAE onset, median (interquartile range) | 10 (5–18.75) |
| **Treatment** |  |
| Steroids | 6 (100) |
| **Evolution** |  |
| Healing of irAE | 5 (83) |
| Persistence of irAE | 1 (17) |

AI, auto-immune; irAE, immune-related adverse event; SD, standard deviation. All values are n (%) unless otherwise noted.
