# Supplementary material for: Immune-related adverse events associated with programmed cell death protein-1 and programmed cell death ligand 1 inhibitors for non-small cell lung cancer: a PRISMA systematic review and meta-analysis
Source: BMC Cancer. 2019 Jun 10;19:558. doi: 10.1186/s12885-019-5701-6 (PMC6558759; doi:10.1186/s12885-019-5701-6)
Supplement: Supplementary file 5 — Table S4. The Cochrane Collaboration’s tool for assessing risk of bias of RCTs. Table S5. Newcastle–Ottawa Scale (NOS) for quality assessment of non-RCTs. (DOCX 43 kb) [file 12885_2019_5701_MOESM5_ESM.docx]

**Supplementary Table 4** The Cochrane Collaboration’s tool for assessing risk of bias of RCT.

|  | Random sequence generation | Allocation concealment | Blinding of participants and personnel | Blinding of outcome assessment | Incomplete outcome data | Selective outcome reporting | Other source of bias |
| --- | --- | --- | --- | --- | --- | --- | --- |
| Antonia et al. (2017)^4^ | ? | n/a | ? | + | + | + | ? |
| Borghaei et al. (2015)^5^ | ? | n/a | - | - | + | + | ? |
| Brahmer et al. (2015)^6^ | ? | n/a | - | - | + | + | ? |
| Carbone et al. (2017)^7^ | ? | - | - | + | + | + | ? |
| Fehrenbacher et al. (2016)^8^ | + | - | - | - | + | + | ? |
| Herbst et al. (2016)^13^ | + | + | - | - | + | + | ? |
| [Reck et al. (2016)](https://www-ncbi-nlm-nih-gov-u.vtrus.net/pubmed/?term=Reck%20M%5BAuthor%5D&cauthor=true&cauthor_uid=27718847)^15^ | ? | - | - | + | + | + | ? |
| Rittmeyer et al. (2017)^16^ | + | - | - | - | + | + | ? |

The dark green cells (+) indicate a low risk of bias. The red cells (-) indicate a high risk of bias. The yellow cells (?) indicate an uncertain risk of bias. n/a, not applicable

|  | **Selection** | **Comparability** | **Exposure/outcome** | **Overall star rating** |
| --- | --- | --- | --- | --- |
| Garassino et al. (2018)^9^ | +++ | + | ++ | 6 |
| Garon et al. (2015)^10^ | +++ |  | ++ | 5 |
| Gettinger et al. (2016)^11^ | +++ |  | ++ | 5 |
| Gettinger et al. (2015)^12^ | +++ |  | ++ | 5 |
| Peters et al. (2017)^14^ | +++ | + | +++ | 7 |
| Rizvi et al. (2015)^17^ | +++ | ++ | ++ | 7 |
| Gulley et al. (2017)^18^ | +++ |  | ++ | 5 |
| Waterhouse et al. (2018)^19^ | +++ | ++ | ++ | 7 |

**Supplementary Table 5** Newcastle-Ottawa Scale (NOS) for quality assessment of non-RCT.

A star system was used for allow a semi quantitative assessment of study quality. A study was awarded a maximum of one star for each numbered item within the selection and exposure categories. A maximum of two stars were awarded for comparability. The NOS ranges from zero to nine stars. We considered high-quality studies as those that achieved seven or more stars, medium-quality studies those with four to six stars, and poor-quality studies those with fewer than four stars.
