# Supplementary material for: Immune-related adverse events associated with programmed cell death protein-1 and programmed cell death ligand 1 inhibitors for non-small cell lung cancer: a PRISMA systematic review and meta-analysis
Source: BMC Cancer. 2019 Jun 10;19:558. doi: 10.1186/s12885-019-5701-6 (PMC6558759; doi:10.1186/s12885-019-5701-6)
Supplement: Supplementary file 6 — Figure S45. Qualitative and quantitative assessment of small-study effects on incidence of global irAEs with anti-PD-1 and anti-PD-L1. (DOCX 93 kb) [file 12885_2019_5701_MOESM6_ESM.docx]

**Additional File 6 –** Qualitative and quantitative assessment for small-study effects of incidence of global irAEs with anti-PD-1 and anti-PD-L1.

B

A


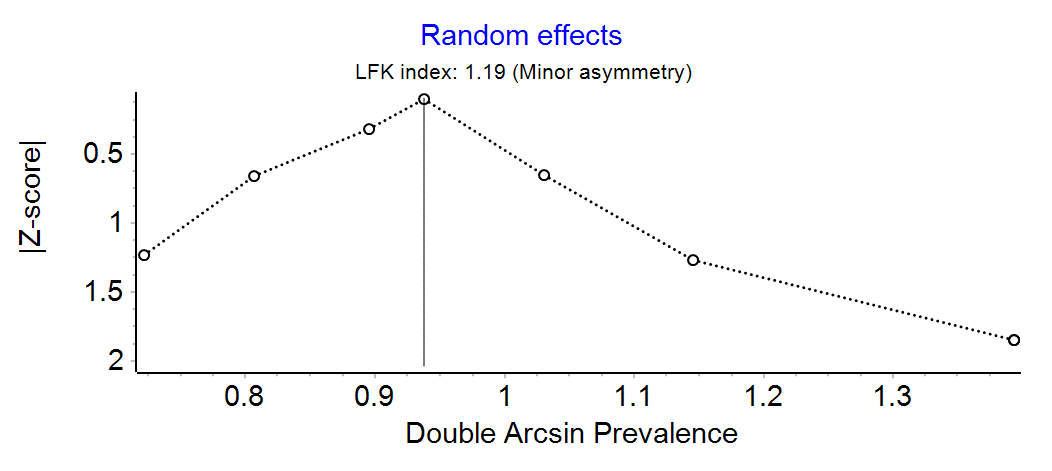

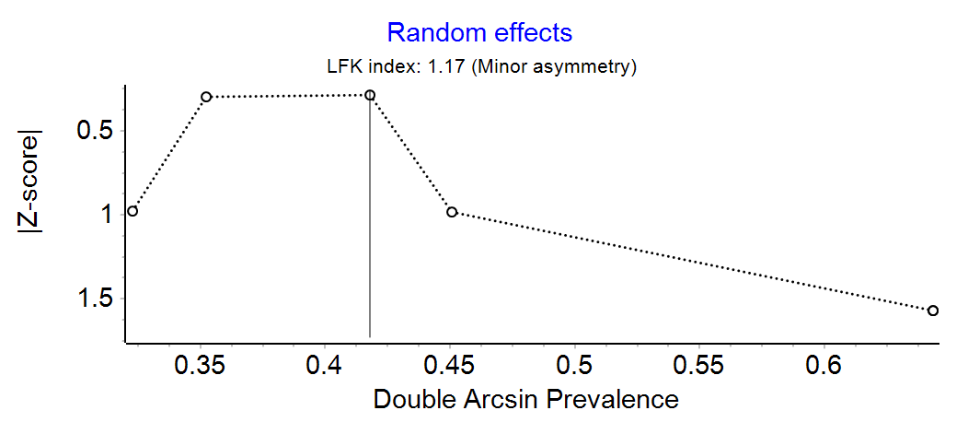


**Supplementary fig. 45** - Qualitative and quantitative assessment for small-study effects of incidence of global irAEs with anti-PD-1 and anti-PD-L1, all-grade (A) and severe grade (B).
